# Integrated molecular and multiparametric MRI mapping of high-grade glioma identifies regional biologic signatures

**DOI:** 10.1038/s41467-023-41559-1

**Published:** 2023-09-28

**Authors:** Leland S. Hu, Fulvio D’Angelo, Taylor M. Weiskittel, Francesca P. Caruso, Shannon P. Fortin Ensign, Mylan R. Blomquist, Matthew J. Flick, Lujia Wang, Christopher P. Sereduk, Kevin Meng-Lin, Gustavo De Leon, Ashley Nespodzany, Javier C. Urcuyo, Ashlyn C Gonzales, Lee Curtin, Erika M. Lewis, Kyle W. Singleton, Timothy Dondlinger, Aliya Anil, Natenael B. Semmineh, Teresa Noviello, Reyna A. Patel, Panwen Wang, Junwen Wang, Jennifer M. Eschbacher, Andrea Hawkins-Daarud, Pamela R. Jackson, Itamar S. Grunfeld, Christian Elrod, Gina L. Mazza, Sam C. McGee, Lisa Paulson, Kamala Clark-Swanson, Yvette Lassiter-Morris, Kris A. Smith, Peter Nakaji, Bernard R. Bendok, Richard S. Zimmerman, Chandan Krishna, Devi P. Patra, Naresh P. Patel, Mark Lyons, Matthew Neal, Kliment Donev, Maciej M. Mrugala, Alyx B. Porter, Scott C. Beeman, Todd R. Jensen, Kathleen M. Schmainda, Yuxiang Zhou, Leslie C. Baxter, Christopher L. Plaisier, Jing Li, Hu Li, Anna Lasorella, C. Chad Quarles, Kristin R. Swanson, Michele Ceccarelli, Antonio Iavarone, Nhan L. Tran

**Affiliations:** 1 Department of Radiology, Mayo Clinic Arizona, Phoenix, AZ USA; 2 Department of Cancer Biology, Mayo Clinic Arizona, Scottsdale, AZ USA; 3 Department of Neurological Surgery, Mayo Clinic Arizona, Scottsdale, AZ USA; 4 Department of Neurological Surgery, Sylvester Comprehensive Cancer Center, Miller School of Medicine, University of Miami, Miami, FL USA; 5 Mayo Clinic Alix School of Medicine Minnesota, Rochester, MN USA; 6 Department of Molecular Pharmacology and Experimental Therapeutics, Mayo Clinic, Rochester, MN USA; 7 Department of Electrical Engineering and Information Technologies, University of Naples, “Federico II”, I-80128 Naples, Italy; 8 BIOGEM Institute of Molecular Biology and Genetics, I-83031 Ariano Irpino, Italy; 9 Department of Hematology and Oncology, Mayo Clinic Arizona, Phoenix, AZ USA; 10 Mayo Clinic Alix School of Medicine Arizona, Scottsdale, AZ USA; 11 H. Milton Stewart School of Industrial and Systems Engineering, Georgia Institute of Technology, Atlanta, GA USA; 12 Department of Neuroimaging Research, Barrow Neurological Institute, Dignity Health, Phoenix, AZ USA; 13 School of Biological and Health Systems Engineering, Arizona State University, Tempe, AZ USA; 14 Imaging Biometrics, LLC, Elm Grove, Milwaukee, USA; 15 Department of Cancer Systems Imaging, University of Texas MD Anderson Cancer Center, Houston, TX USA; 16 Quantitative Health Sciences, Mayo Clinic Arizona, Scottsdale, AZ USA; 17 Division of Applied Oral Sciences & Community Dental Care, The University of Hong Kong, Hong Kong SAR, China; 18 Department of Neuropathology, Barrow Neurological Institute, Dignity Health, Phoenix, AZ USA; 19 Department of Psychology, Hunter College, The City University of New York, New York, NY USA; 20 Department of Psychology, The Graduate Center, The City University of New York, New York, NY USA; 21 Avinger Incorporated, Redwood City, CA USA; 22 Department of Speech and Hearing Science, Arizona State University, Tempe, AZ USA; 23 Department of Neurosurgery, Barrow Neurological Institute, Dignity Health, Phoenix, AZ USA; 24 Department of Neurosurgery, Banner University Medical Center, University of Arizona, Phoenix, AZ USA; 25 Department of Pathology, Mayo Clinic Arizona, Phoenix, AZ USA; 26 Department of Neurology, Mayo Clinic Arizona, Phoenix, AZ USA; 27 Jensen Informatics LLC, Shorewood, WI USA; 28 Departments of Biophysics and Radiology, Medical College of Wisconsin, Milwaukee, WI USA; 29 Departments of Psychiatry and Psychology, Mayo Clinic, AZ USA; 30 Department of Biochemistry and Molecular Biology, Sylvester Comprehensive Cancer Center, Miller School of Medicine, University of Miami, Miami, FL USA; 31 Department of Public Health Sciences, Sylvester Comprehensive Cancer Center, Miller School of Medicine, University of Miami, Miami, FL USA

**Keywords:** Cancer genomics, Cancer imaging

## Abstract

Sampling restrictions have hindered the comprehensive study of invasive non-enhancing (NE) high-grade glioma (HGG) cell populations driving tumor progression. Here, we present an integrated multi-omic analysis of spatially matched molecular and multi-parametric magnetic resonance imaging (MRI) profiling across 313 multi-regional tumor biopsies, including 111 from the NE, across 68 HGG patients. Whole exome and RNA sequencing uncover unique genomic alterations to unresectable invasive NE tumor, including subclonal events, which inform genomic models predictive of geographic evolution. Infiltrative NE tumor is alternatively enriched with tumor cells exhibiting neuronal or glycolytic/plurimetabolic cellular states, two principal transcriptomic pathway-based glioma subtypes, which respectively demonstrate abundant private mutations or enrichment in immune cell signatures. These NE phenotypes are non-invasively identified through normalized K2 imaging signatures, which discern cell size heterogeneity on dynamic susceptibility contrast (DSC)-MRI. NE tumor populations predicted to display increased cellular proliferation by mean diffusivity (MD) MRI metrics are uniquely associated with *EGFR* amplification and *CDKN2A* homozygous deletion. The biophysical mapping of infiltrative HGG potentially enables the clinical recognition of tumor subpopulations with aggressive molecular signatures driving tumor progression, thereby informing precision medicine targeting.

## Abstract (web summary)

Glioma tumours are known to be heterogenous in mutation and gene expression patterns, but sampling limitations can lead to inaccurate detection of evolutionary events. Here, the authors carry out multi-omics analysis of multi-regional biopsies from 68 patients and show differential mutations in non-enhancing regions.

## Introduction

High-grade gliomas (HGG) are aggressive primary brain malignancies that confer a universally fatal outcome due to the inability to therapeutically control diffuse cell invasion throughout the brain parencyhma^1–5^. Invasive tumor, as defined by the residual populations left behind after standard gross total surgical resection of T1-weighted magnetic resonance imaging (MRI) contrast-enhancing (CE) tumor, contributes universally to disease progression and recurrence. The molecular landscape and therapeutic susceptibilities of these tumor segments remain largely unaddressed. Past studies have inferred the genomic evolution of the invasive tumor fraction by comparing non-localized bulk samples^6,7^, without a uniform, clinically relevant definition of invasive tumor populations. Here, we use a stereotactic MRI-guided sampling approach to profile invasive tumor, as defined by the non-enhancing (NE) fraction that resides beyond gadolinium uptake on T1W MRI. This represents a reproducible approach that leverages a standardized clinical metric for the identification of invasive tumor subpopulations.

The molecular trajectories of brain tumor cells during invasion and disease progression remain to be charted. It also remains unclear to which extent the geographic evolution of brain tumors impacts tumor cell programs, regional therapeutic sensitivities, and the interaction with the tumor microenvironment. There is shared consensus that any effort aimed at dissecting the intratumor heterogeneity that fuels brain tumor evolution requires the characterization of high-resolution multi-regional samples at different molecular levels^8^. Recent characterization of HGG subclassification combines multiple dimensions of data to define a pathway-based deconvolution of the most active biological functions and confers clinical implications. These classifications include cellular states distributed along a metabolic axis (glycolytic/plurimetabolic (GPM) and mitochondrial (MTC)) and a neurodevelopmental axis (proliferative/progenitor (PPR), and neuronal (NEU)) with high prognostic significance characterized by subtype-specific vulnerabilities^9^. More recently, the four subtypes were orthogonally validated across distinct multi-omics platforms, confirming the ability to predict clinical outcome and therapeutic vulnerabilities when applied to tumor types beyond brain tumors, thus underscoring the general relevance of the biological hallmarks associated with each of the pathway-based subtypes^10^.

Due to the surgical inaccessibility of invasive NE tumor, the incorporation of MRI-imaging features can be used to enhance the molecular profiling of this tumor segment. Advanced multi-parametric MRI techniques, including dynamic susceptibility contrast (DSC)-MRI and diffusion tensor imaging (DTI), provide innovative imaging tools that can phenotypically characterize the invasive NE tumor to improve clinical diagnosis, presurgical planning, and prognostication compared to conventional MRI alone. DSC-MRI measures of relative cerebral blood volume (rCBV) have been utilized to predict tumor grade^11–13^, prognosis^14,15^, and to differentiate recurrent tumor from post-treatment effects^16–18^ based on regional differences in microvessel volume. DTI measures of bulk water diffusion (mean diffusivity, MD) and directionally dependent water diffusion (fractional anisotropy, FA) are commonly used in neuro-oncology to identify cellular packing^19^ and to observe tumor infiltration of white matter tracts, respectively^20^. Other advanced measurements from DSC-MRI (e.g., K2, nK2, EPI + C) remain understudied. Despite the ubiquity of MRI in the clinical setting, limited studies have attempted to spatially define the underlying molecular associations between multi-parametric MRI phenotypic features and invasive NE subpopulations in HGG.

Here, we seek to integrate advanced multi-parametric MRI features with spatially matched multi-regional genomic and pathway-based deconvolution across 313 MRI-localized biopsies from 68 HGG patients to provide a comprehensive study of invasive NE tumor biology. In the invasive NE region, we observe an increased proportion of private mutations as well as intratumoral mosaicism of key driver alterations, including *EGFR* amplification and *NF1* inactivation. Multiregional molecular profiles of a subset of IDH wild-type tumors predict distinct trajectories of the molecular evolution. The NE regions are enriched in a mutually exclusive fashion with the NEU and GPM pathway-based subtypes that can now be non-invasively distinguished using the DSC-MRI metric nK2^9^. Together, these findings provide insight into the molecular landscape of NE HGG populations and delineate an expanded role for imaging metrics to identify biologically aggressive tumor regions that better inform future targeted therapy.

## Results

### Multiregional cohort of IDH wild-type and IDH-mutant high-grade gliomas

**Fig. 1:**
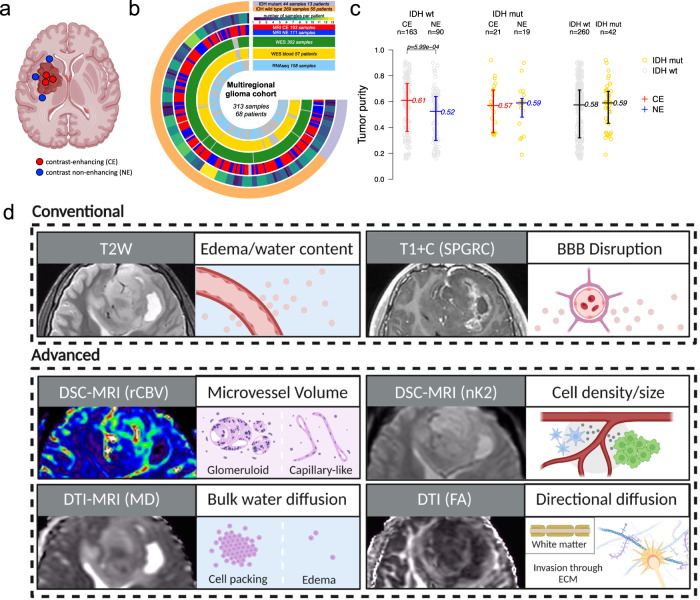
Multiregional biopsy and MRI-based tumor sampling from a cohort of glioma patients. **a** MRI contrast enhancement-based sampling of glioma specimens. **b** Circos plot indicating the molecular assay and MRI annotation of multiregional samples. **c** Tumor purity has been inferred from WES (available for 302 samples) and compared between contrast-enhancing (CE) and contrast non-enhancing (NE) samples within IDH wild-type group (left; MRI annotated IDH wild-type samples = 253), within IDH-mutant group (middle; MRI annotated IDH-mutant samples = 40), and between IDH wild-type and IDH-mutant samples (right). The middle line corresponds to the median; the lower and upper lines show the first and third quartiles. Difference of the purity mean among groups was assessed using the two-sided Mann–Whitney–Wilcox test. Source data are provided as a Source data file. **d** Schematic of imaging features extracted for this study and their phenotypic correlates. Features are separated into conventional and advanced MRI features.

We used intraoperative neuronavigation assistance to sample patient tumors in spatially distinct locations in CE and NE regions (median = 4 samples per tumor, range = 1–13 samples per tumor; median = 3 CE samples per tumor, range = 1–8 CE samples per tumor; median = 2 NE samples per tumor, range = 1–9 NE samples per tumor) (Fig. 1a). Tumor samples (including 193 CE and 111 NE annotated samples) were filtered for quality assessment and profiled by whole exome (WES, *n* = 302) and RNA sequencing (RNAseq, *n* = 158). Matched patient whole blood was available for WES for 57 patients (Fig. 1b; Supplementary Data 1). The patient cohort included 269 IDH wild-type and 44 IDH-mutant samples. In IDH wild-type tumors, NE samples demonstrated lower tumor purity (median 0.52), as inferred from WES copy number profiles, compared to a median of 0.61 in the CE region (*p* = 5.99e−04). Lower tumor purity indicates the increased presence of non-malignant cells in the NE (Fig. 1c). In contrast, IDH-mutant tumors did not differ in tumor purity when comparing CE and NE regions, and no difference in tumor purity was detected when stratifying by IDH mutation status (Fig. 1c). For each biopsy location, we extracted spatially matched imaging phenotypes (Fig. 1d) from multiparametric advanced MRI performed at the time of pre-operative scanning for surgical planning.

### IDH-mutant gliomas harbor intratumoral heterogeneity with aggressive molecular features

**Fig. 2:**
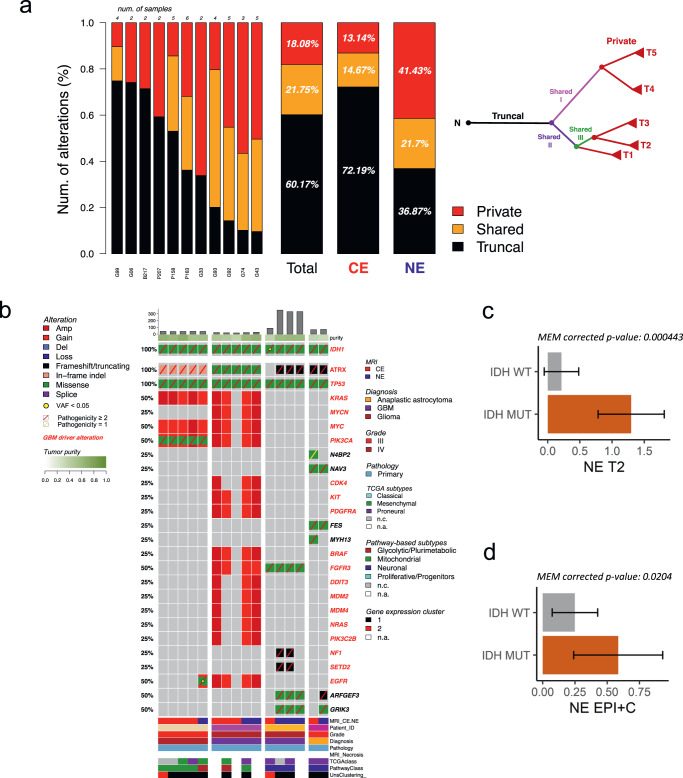
Somatic genetic mutations, copy number alterations, and correlates of imaging features in IDH-mutant glioma. **a** For each IDH-mutant tumor (listed on horizontal axis), genetic variants have been annotated as private (exclusively occurring in one sample), shared (occurring in two or more samples, but not in all samples) and truncal (occurring in all samples) and reported as a percentage of the total number of somatic variations. The proportion of mutation types was significantly different between CE and NE (two-sided Fisher’s exact test *p* = 3.43e−67). A schematic example of multiregional tumor evolution is represented as a tree in which truncal, shared, and private branches are distinguished. **b** Overview of somatic alterations in IDH-mutant samples grouped by patient. Mutation load and tumor purity are reported in the top barplot and heatmap track, respectively. Clinical annotation and gene expression classification are indicated in the bottom tracks. Gene alteration frequency in the patient cohort is indicated as percentage on the left, known with driver mutations highlighted in red. **c** MEM model derived estimated marginal mean of T2W in IDH-mutant vs. IDH wild-type samples in the NE. Error bars show 95% confidence interval. Two-sided t-test with Tukey correction (*n* = 86). **d** MEM model derived estimated marginal mean of EPI + C in IDH-mutant vs. IDH wild-type samples in the NE. Error bars show 95% confidence interval. Two-sided t-test with Tukey correction (n = 89). **c**, **d** Data are presented as mean values +/−SD. **a**, **c**, **d** Source data are provided as a Source data file.

Within gliomas, IDH mutation confers a longer survival than IDH wild-type. However, there is a subset of IDH-mutant glioma that exhibits more aggressive biology and shorter overall survival^21–23^. The evolution of genomic alterations associated with rapid clinical progression in IDH-mutant glioma remains poorly defined. We profiled 40 multiregional samples within 11 IDH-mutant tumors to determine shared early versus regional acquired alterations (Fig. 2a). On average, 60.17% of mutations were truncal (shared across all the biopsies from a particular tumor), 21.75% shared (shared across some but not all biopsies from any tumor), and 18.80% private (present in a single biopsy). IDH-mutant tumors demonstrated an increased burden of private mutations in the NE (41.43%) compared to the CE (13.14%) (Fig. 2a).

Alterations in the receptor tyrosine kinase *EGFR* occur frequently in IDH wild-type glioma but are rarely reported in IDH-mutant tumors^24,25^. Upon examination of four IDH-mutant tumors with NE and CE samples available (Fig. 2b), we identified two IDH-mutant tumors exhibiting heterogeneous *EGFR* alteration, including the presence of a low allele frequency (2%) *EGFR* A289V gain-of-function mutation unique to the NE region in one patient tumor (Fig. 2b). *EGFR* A289D/T/V is associated with shorter patient survival in IDH wild- type glioblastoma (GBM) but remains poorly characterized in IDH-mutant tumors^26^. We also observed regional heterogeneity in genes associated with poor clinical prognosis in IDH-mutant glioma, including *CDKN2A/B, CDK4, MYC, MYCN*, and *PDGFRA*^23^ (Fig. 2b, Supplementary Fig. 1; Supplementary Data 1).

Next, we evaluated multi-parametric MRI features (Fig. 2c, d, Supplementary Fig. 2) across IDH status. IDH status explained significant proportions of the variance for several features in the overall and NE-specific cohorts (Supplementary Fig. 3a). IDH-mutant tumors displayed significantly higher T2W signal compared to IDH wild-type tumors in the NE (Fig. 2c & Supplementary Fig. 3b–e), corroborating findings from other cohorts^27–29^. EPI + C signal was also significantly higher in IDH-mutant tumors, but the amount of T2W contribution driving this phenomenon is unclear as the biophysical underpinnings of EPI + C, particularly in tissue disrupted by tumor, are poorly understood (Fig. 2d).

### The phylogeny of molecular alterations in IDH wild-type glioma is distinct across regions of MRI-based contrast enhancement

**Fig. 3:**
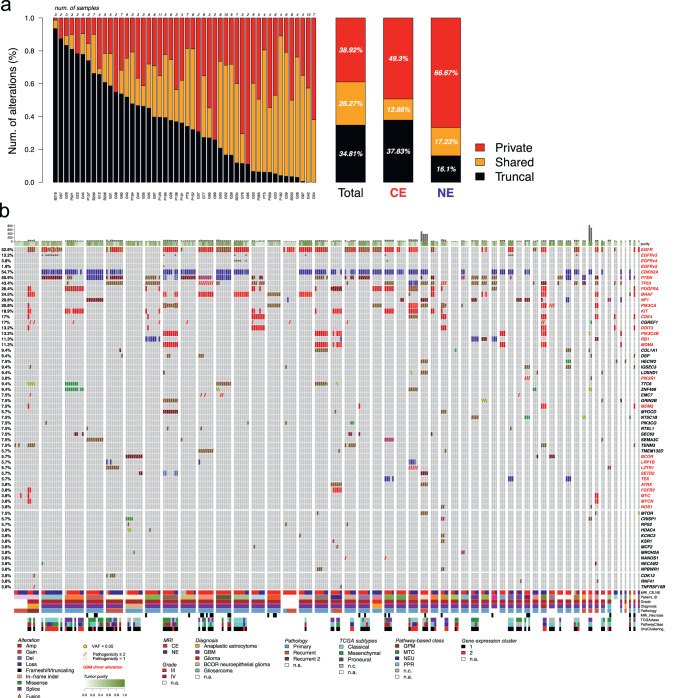
Molecular alteration landscape of IDH wild-type glioma. **a** The spectrum of somatic genetic alterations occurring in multiregional samples (*n* = 255) from IDH wild-type glioma patients (*n* = 48) indicated the frequency of truncal, shared, and private events within each single patient (left bars), within all patients (right bar, Total), and within contrast-enhancing and contrast non-enhancing samples (right bars, CE, and NE, respectively). The proportion of mutation types was significantly different between CE and NE (two-sided Fisher’s exact test *p* = 4.85e−43). Source data are provided as a Source data file. **b** Somatic genetic mutations and copy number alterations occurring in IDH wild-type glioma (260 samples from 53 patients). Samples are grouped by patients. Mutation load and tumor purity are reported in the top barplot and heatmap track, respectively. Clinical annotation and gene expression classification are indicated in the bottom tracks. Gene alteration frequency in patient cohort is indicated as percentage on the left.

We analyzed the genetic evolution of 255 intratumor multiregional samples from 48 IDH wild-type (IDHwt) glioma patients using PhyC^30^ on the total, CE, and NE cohorts (Fig. 3a). On average, 34.81% of total alterations were truncal, 26.27% shared and 38.92% private (Fig. 3a). The proportion of private mutations ranged from >50% to <10%. Compared to IDH-mutant glioma, IDHwt tumors harbored a significantly higher proportion of private alterations in the NE (66.7%) compared to CE (49.3%), with only 16.1% of alterations occurring as truncal events (Fig. 3a). This suggests that glioma cells on the periphery of the tumor exhibit early evolutionary divergence relative to their central tumor counterparts, consistent with other studies^31^. The frequency of driver alterations in our dataset recapitulates trends seen in other large genomic studies of GBM and corroborates previous reports of intratumoral heterogeneity of driver alterations including in *EGFR, CDKN2A, PTEN, TP53, PDGFRA, BRAF, NF1, PIK3CA*, and *KIT*, among others (Fig. 3b; Supplementary Data 1)^32,33^.

### *EGFR* and *NF1* mosaicism in IDH wild-type glioma

**Fig. 4:**
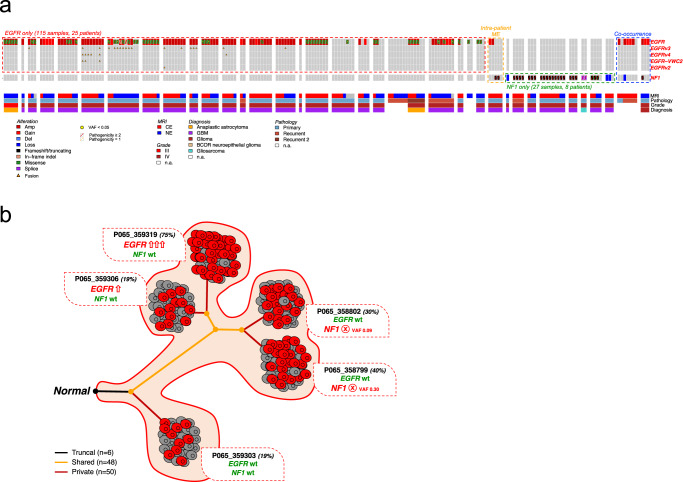
Mutual genetic alteration profiles of EGFR and NF1 in IDH wild-type glioma. **a** *EGFR* and *NF1* somatic genetic alterations occurring in multiregional samples from IDH wild-type glioma. Samples are grouped by patient and clinical annotations are indicated in the bottom tracks. *EGFR* (red box) and *NF1* (green box) alterations are mutually exclusive in 98.7% samples (152 out of 154, two-sided Fisher’s Exact test *p* = 1.72e−07). The mutual exclusivity between *EGFR* and *NF1* alterations was also observed within the same patient (orange box). Co-occurring genetic alterations have been identified only in 2 samples from 2 patients (blue box). **b** Evolutionary model of glioblastoma from patient P065 that included 5 contrast-enhancing samples harboring mutual exclusive EGFR and NF1 alterations. NF1 truncating mutation specifically occurred in two samples with wild-type EGFR locus; conversely, EGFR was amplified in the other two NF1 wild-type samples. Tumor purity is indicated for each sample (percentages displayed). VAF, variant allele frequency. Number of truncal, shared, and private alterations are indicated.

Intratumoral mosaicism of driver alterations in GBM is well established^33–35^, with specific alterations occurring in a mutually exclusive fashion^36^. Here we explored the alteration profiles of *EGFR* and *NF1* in our IDH wild-type cohort (Fig. 4a). We determined *EGFR* and *NF1* alterations to be mutually exclusive between individual patient tumors in 98.7% (152/154) of mutated samples from 30 treatment-naive primary tumors and 5 recurrences. While most tumors exhibited mutual exclusivity of *EGFR* and *NF1* alterations across all samples, we resolved one patient tumor, P065 (Fig. 4a, orange box) which displayed intratumoral mutual exclusivity across distinct multiregional samples, with an *NF1* truncating mutation specifically occurring in two *EGFR* wild type samples, and conversely, *EGFR* amplification in the other two *NF1* wild type samples. The evolutionary model of P065 tumor was predicted by comparing the genomic alteration profiles among five multiregional samples, showing that the molecular evolution diverged into two main branches with the acquisition of *EGFR* amplification on one branch, and the occurrence of *NF1* mutation on the other branch (Fig. 4b). The mutual exclusivity of *EGFR* and *NF1* alterations was also validated in three independent HGG datasets: 378 samples from TCGA-GBM (95.6%), 292 samples from the GLASS consortium (92.1%)^6^ and 94 samples from Wang et al. (85.9%)^37^ (Supplementary Fig. 4). In the GLASS consortium longitudinal HGG cohort, mutual exclusivity was significantly more common than co-occurrence in primary tumors (*p* = 6.8e−06; Supplementary Fig. 4). In the recurrent samples, while mutual exclusivity was still more common than co-occurrence, the total number of co-occurrences increased (*p* = 0.027; Supplementary Fig. 4). This trend also persisted in the primary and recurrent samples of the Wang et al. cohort. We also observed co-occurrence of *EGFR* and *NF1* alterations in two samples from two patients (Fig. 4a, blue box). Notably, the cases of co-occurrence arise in a recurrent sample (M78J in blue box, Fig. 4a) and in the NE of a primary sample (G95I in blue box, Fig. 4a). *NF1* and *EGFR* co-altered samples thus may be phenotypically significant in tumor recurrence but rarely detected from the analysis of single biopsies or those originating from the resected portion of the CE tumor.

### Spatial and molecular heterogeneity of *EGFR* in GBM

While mosaic amplification of HGG drivers is postulated to underlie treatment resistance^33^, HGG is further complicated by the heterogeneity of alterations found within individual drivers. *EGFR* is altered in the majority of GBM cases, and functional receptor activation occurs through multiple possible mechanisms, including amplification, mutation, rearrangement, and/or altered splicing^38^. In our cohort, *EGFR* harbored somatic genetic alterations in 26 out of 50 multiregional IDH wt cases, with 92% of mutant cases (23 out of 26) showing molecular intratumor heterogeneity in the *EGFR* locus across the multiregional samples (two-sided Fisher’s exact test *p* = 2.43e−11).

**Fig. 5:**
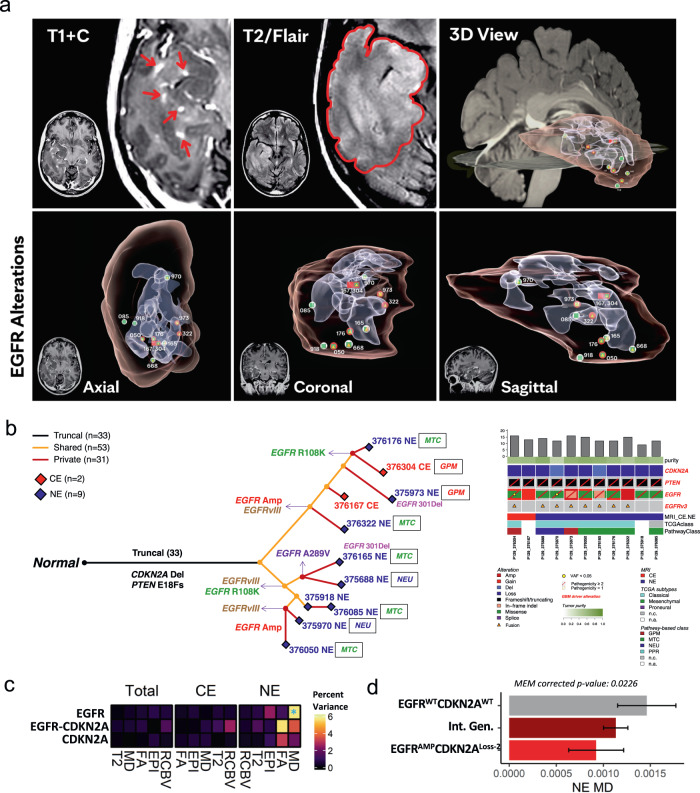
Spatial heterogeneity of EGFR alteration and EGFR-associated imaging phenotypes. **a** Three-dimensional visualization of EGFR alterations and their associated MRI features. **b** The molecular evolution of glioblastoma from patient P129 inferred from the occurrence of genetic alterations as truncal, shared, and private events across the multiregional specimens (*n* = 11, 2 contrast-enhancing and 9 contrast non-enhancing samples). The length of branches in the evolutionary tree (left panel) is proportional to the number of occurring alterations. Truncal driver alterations (CDKN2A deletion and PTEN frameshift mutation), and non-truncal multiple EGFR alterations have been reported along the evolutionary tree and in the oncoprint (right panel). Mutation load, tumor purity, MRI contrast enhancement annotation, and gene expression classification have been reported. **c** The percent variance attributed to each fixed term (y-axis) in MEMs for each imaging variable (x-axis) separated by region. *p-value < 0.05. EGFR*CDKN2A indicates the interaction of EGFR and CDKN2A (*n* = 221 biopsy samples). Statistical test: ANOVA. **d** MEM model derived estimated marginal mean of MD for EGFR and CDKN2A genotypes in the NE. Intermediate genotypes (Int. Gen.) denote genotypes that are not double wild type or EGFR amplified and CDKN2A homozygous loss. Error bars show 95% confidence interval (*n* = 74 biopsy samples). Statistical test: two-sided t-test with Tukey correction. Data are presented as mean values +/−SD. **c**, **d** Source data are provided as a Source data file.

To examine the spatial and molecular heterogeneity at the *EGFR* locus, we selected one treatment-naive tumor (P129) with the highest number of multi-regional biopsies from CE and NE regions for focused analysis. In P129, 3D reconstruction of the CE and NE tumor segments, using T1 + C and T2W/FLAIR, respectively, showed the distribution of MRI-localized biopsy samples and exposed the molecular heterogeneity of *EGFR* at different sites (Fig. 5a). Truncal and private genetic alterations, including the complex local genomic instability of *EGFR*, identified in 2 CE and 9 NE samples were used to infer the molecular trajectory of tumor evolution (Fig. 5b). The evolutionary model indicated that *EGFR* alterations were relatively late events, occurring after other truncal driver alterations, including *CDKN2A* and *PTEN* losses. The genomic instability at the *EGFR* locus likely occurred independently of the selective forces of chemotherapy or radiation, with *EGFRvIII* arising independently twice in P129, once prior to *EGFR* amplification. Additionally, two different *EGFR* missense mutations (R108K and A289V) and a small in-frame deletion (301del) occurred as shared or private mutations in the NE region.

### *EGFR* and *CDKN2A* alterations drive variance in advanced imaging features in the NE region

To broadly characterize how genetic driver aberrations influence imaging phenotypes, we constructed a screening mixed effects model (MEM) with the mutation status and copy number of top genetic drivers of GBM (*EGFR, NF1*, *TP53, PTEN*, and *CDKN2A*) modeled as fixed effects with no interacting terms and patient effects specified as random effects. Copy number variations contributed most strongly to the variance in several imaging features (Supplementary Fig. 5a). Thus, moving forward, we only considered the copy number status of genetic drivers when examining imaging effects. *EGFR* copy number variation stood out as a strong effector of several imaging variables particularly in the NE region, where it explained 54.96% and 15.39% of the variance in rCBV and T2W signal, respectively (Supplementary Fig. 5a). On examination of T2W and rCBV in an only *EGFR* CNV MEM, significant differences between these parameters were not appreciated which indicates that other covariates, such as those accounted for in the screening MEM, were masking *EGFR’*s effect (Table 1). From this result we can surmise that *EGFR* is having an effect on the microvessel volume (rCBV) and water content (T2W) that could be elucidated in a larger cohort. In the *EGFR*-specific MEM, *EGFR* amplified tumors demonstrated a significantly lower MD value relative to wild type in the NE region, but this difference was not observed in the CE (Supplementary Fig. 5b, c). Lower MD has been associated with greater cellular packing and higher tumor proliferative indices^19^.

**Table 1:** Fixed effect formulas used for each MEM model and their corresponding figures or data file

Fixed effects	Figures
IDH_mutation	Fig. 2c, d, Supplementary Fig. 3a–e
EGFR_cnv+EGFR_mut+CDKN2A_cnv+NF1_cnv+NF1_mut+TP53_cnv+TP53_mut+PTEN_cnv+PTEN_mut	Supplementary Fig. 5a
EGFR_cnv	Supplementary Fig. 5b, c
NF1_cnv*EGFR_cnv	Supplementary data 2
CDKN2A_cnv*EGFR_cnv	Fig. 5c, d, Supplementary Fig. 5d, e
TP53_cnv*EGFR_cnv	Supplementary data 2
Pathway-based classification	Supplementary data 2

* denotes both terms as single effects and an interacting term (ex. IDH_mut*EGFR_cnv= IDH_mut+ EGFR_cnv + IDH_mut:EGFR_cnv. “Mut” indicates mutation status (mutated versus wild type) and “cnv” indicates copy number variation.

Given the high prevalence of *EGFR* alterations in GBM^39^ and the impact of *EGFR* on percent variance analysis, we hypothesized that imaging effects may be further influenced by combinatorial genotypes. Due to sample size limitations, it was not possible to construct MEM models with more than two interacting genes. Thus, in this analysis, EGFR was individually paired with each gene separately, resulting in a total of three analyses. This approach allowed us to analyze the effects from combinatorial genotypes of EGFR with each partner gene (CDKN2A, TP53, and NF1) on imaging features (Table 1). To test this, mixed effect models were generated examining *EGFR* copy number in combination with *NF1, TP53*, and *CDKN2A* copy number. Each gene and their interactions were modeled as fixed effects with patients as random effects. While *NF1* and *TP53* combinatorial genotypes did not reveal significant relationships, the interaction of *EGFR* CNV and *CDKN2A* CNV explained significant proportions of some imaging features’ variance (Fig. 5c). ANOVA and subsequent pairwise t-tests revealed that *EGFR* amplification and *CDKN2A* homozygous deletion were associated with a significantly lower MD signal in the NE region specifically when compared to double wild-type tumors (Supplementary Fig. 5c, e and Fig. 5d). Tumors with intermediate genotype of *CDKN2A* and *EGFR* (e.g., *CDKN2A* heterozygous deletion / *EGFR* gain or *CDKN2A* homozygous deletion / *EGFR* wild type) displayed values between the two copy number extremes, indicating that successive *EGFR* gains and *CDKN2A* deletions result in a progressively lower MD signature observable in the NE region exclusively (Supplementary Fig. 5d, e, Fig. 5d). Co-occurrence of *EGFR* amplification with deletion of *CDKN2A* has been reported to synergistically increase cellular proliferation^40–42^. Reduction in MD has been shown to be a biomarker of higher tumor cellularity and aggressiveness on histology (Fig. 1d), particularly within the NE region^19^. These results highlight the potential of MD in the NE region to serve as a predictor of regional genomic status, and in particular, the combination of *EGFR* amplification and *CDKN2A* homozygous deletion and the associated aggressive phenotype.

### Inference of molecular tumor evolution in IDH wild-type glioma

**Fig. 6:**
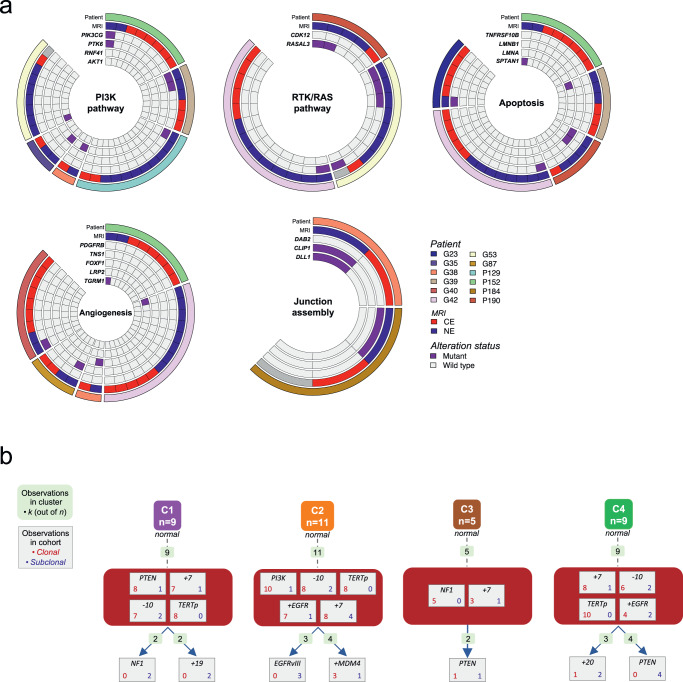
Molecular tumor evolution in IDH wild-type glioma. **a** Alterations exclusive to the NE region are displayed from a subset of IDH wild-type patients. Alterations specific to the NE region were annotated with gene ontology terms and grouped based on ontology similarity. **b** Evolutionary trajectories of genetic alterations have been predicted by comparing the multiregional molecular profiles in a subset of IDH wild-type glioma patients (*n* = 34) with more than one truncal driver event identified. Four evolutionary models of IDH wild-type glioma have been proposed from the supervised clustering of repeated initiating trajectories. The number of trajectories observed within each cluster is reported (light green boxes); the number of clonal (red) and subclonal (blue) events is indicated for each alteration.

The multiregional genetic profiling of IDH wild-type HGGs revealed divergent molecular evolution in the invasive NE tumor (Fig. 3a). To characterize the functional impact of subclonal genomic alterations occurring as private events in the tumor periphery, we explored the individual genes that were exclusively altered in the NE regions, and we identified the pathways and molecular functions associated with these genes. NE alterations independently arose across several patients within several predominant signaling pathways known to contribute to tumor progression including the PI3K pathway, RTK/RAS pathway, apoptosis, angiogenesis, and junction assembly pathways (Fig. 6a). These findings are also consistent with previous results indicating that activation of the PI3K and RTK/RAS pathway is retained in recurrent GBM^43^.

To predict the trajectories of molecular events that activate NE-specific tumor functions we compared the genetic profiles across multiple samples from the same tumors. Specifically, we used REVOLVER to build genomic models of glioma evolution^44^. Using this approach, we utilized 34 IDH wild-type informative patients, defined as those having more than one driver clonal alteration (Supplementary Fig. 6), to infer the mutation clones populating each tumor and characterize them in terms of number of driver alterations and composition of clonal and subclonal events. A supervised hierarchical clustering was applied to compare the tumors and distinguish common patterns of genomic trajectories, suggesting four putative models of genetic evolution (Fig. 6b). Canonical glioma genetic alterations occurred in most tumors as initiating truncal events, including *TERT* promoter mutations, chromosome 7 duplication and chromosome 10 monosomy. The glioma evolution, instead, is predicted to be supported by the acquisition of subclonal alterations in different cancer driver genes. In two inferred evolutionary models, PI3K pathway drives the gliomagenesis as a consequence of *PTEN* truncal inactivation or *PI3K* activating genetic mutations, whereas the tumor progression is sustained by the over-activation of RTK/RAS signaling through the occurrence of subclonal *NF1* and *EGFR* alterations in C1 and C2 models, respectively. Conversely, when the RTK/RAS pathway drives gliomagenesis (by *NF1* inactivation and *EGFR* amplification in C3 and C4 models, respectively), the inferred tumor progression relies on the acquisition of *PTEN* alterations, and subsequent up-regulation of the PI3K pathway. The evolutionary modeling of IDH wild-type HGGs indicated the complementary longitudinal activation of PI3K and RTK/RAS pathways along two main alternative trajectories of molecular evolution.

### Multiregional transcriptomic and microenvironmental landscape of high-grade glioma

**Fig. 7:**
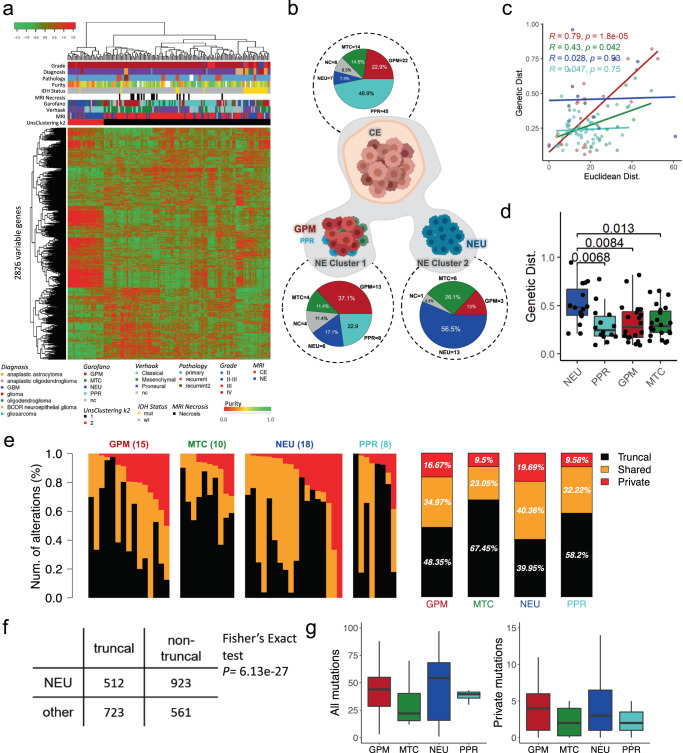
Conventional MRI, transcriptomic, and genotypic characterization of NE region phenotypes. **a** Unsupervised hierarchical clustering of the 158 multiregional glioma samples; rows are the 2826 most variable genes. **b** Pie charts show the frequencies of pathway-based classifications of MRI CE samples (top) and MRI NE samples of unsupervised cluster 1 and 2, respectively (bottom). **c** Correlations between the genotypic and euclidean distance of paired samples with the same pathway-based classification. **d** Genetic distance of CE samples to NE samples of each pathway-based classification (*n* = 94 biopsy samples). Boxplots represent data minimum, 25th percentile, 50th percentile, 75th percentile, and maximum. The *p*-values are indicated above each comparison in the figure. **e** Private, shared, and truncal alterations in individual samples in the NE region classified as each pathway-based subtype (from left to right: glycolytic/plurimetabolic, mitochondrial, neuronal, and proliferative/progenitor), with the average of private, shared, and truncal mutations for each pathway-based classification displayed to the right. **f** The proportion of truncal mutations vs. non-truncal (private and shared) mutations in samples of NEU subtype was significantly different than the proportion of truncal mutations vs. non-truncal mutations in the other subtypes (one-tailed Fisher’s exact test *p* = 6.13e−27). **g** Box and whisker plots show the absolute number of total (left) and private (right) mutations in each pathway-based classification and the distribution of mutational burden across samples (*n* = 51 biopsy samples). Boxplots represent data 25th percentile, 50th percentile, and 75th percentile. The upper whisker extends from the upper hinge to the largest value no further than 1.5X IQR (inter-quartile range, or distance between the first and third quartiles) and the lower whisker extends from the lower hinge to the smallest value at most 1.5X IQR. **c**, **d**, **e**, **g** Source data are provided as a Source data file.

Unsupervised hierarchical clustering on the most variable genes of 158 multiregional glioma samples revealed two distinct transcriptional groups (Fig. 7a). We observed a significant association (*p* = 1.5e−7, Chi-squared test) between unsupervised clusters and tumor regions, with 95% of CE samples falling in cluster 1 (Fig. 7a, black). NE samples were equally distributed between the two clusters, with cluster 2 (Fig. 7a, red) enriched for NE samples (80%). To verify that our clustering analysis was not unbalanced towards individual patients, we visualized how patients segregated into each cluster and found that multiple patients contributed to both clusters without specific bias (Supplementary Fig. 7).

Next, we classified each sample according to the TCGA classification^36^ and the single cell-derived, pathway-based classification^9^ (Supplementary Data 3). Whereas the TCGA subtypes were not significantly associated with spatially-resolved tumor regions (*p* = 0.323, Chi-squared test), the pathway-based subtypes were significantly enriched in a region-specific manner (*p* = 8.3e−5, Chi-squared test). More specifically, 19 out of 26 (73%, *p* = 2.3e−3, Fisher Exact test) NEU samples were found in NE regions. Conversely, we identified most PPR samples in CE regions (45 out of 53, 84%, *p* = 2.7e−12, Fisher Exact test). The significance of the association between the pathway-based subtypes and imaging features also emerged from the inspection of the composition of the unsupervised clusters (*p* = 2.18e−12, Chi-squared test). Cluster 1, enriched in CE biopsies, contained 92% of GPM samples (38 out of 41, *p* = 5.9e−14, Fisher Exact test), whereas cluster 2, enriched in NE biopsies, contained 67% of NEU samples (18 out of 27, *p* = 0.03, Fisher Exact test) (Supplementary Fig. 8a). Conversely, the NE samples falling in cluster 1 were mostly of the GPM subtype (Fig. 7b). Overall, invasive NE samples appeared to belong to two main transcriptional phenotypes, GPM and NEU (Fig. 7b). Consistent with the biological pathways marking each individual subtype^9^, NE samples in cluster 1 (GPM-enriched) exhibited activation of glycolysis/hypoxia-related functions and signatures of myeloid immune cells. Conversely, the NE cluster 2 (NEU-enriched) showed a neuronal functional profile, including neurotransmitter secretion, synaptic plasticity, regulation of membrane potential, markers of mature neurons (*NEFM*, *RBFOX3*, *NEFH*, *SYP,* and *DLG4*), glutamatergic neurons (*SLC17A6*, *GRIN2B*, *GRIN1*, *SLC17A7,* and *GLS*), dopaminergic neurons (*KCNJ6*) and GABAergic neurons (*GAD2*) (Supplementary Data 1)^9^.

### Samples classified as NEU exhibit the highest burden of private mutations in the NE region

Previous reports^45,46^ have demonstrated a positive correlation between physical distance and genetic distance between tumor cells. To capture the relationships between spatially resolved samples, we calculated the Euclidean and genetic distances between any combination of sample pairs from any given patient. Genetic distance (computed as 1 - Jaccard index on the genetic alteration patterns) measures the divergence between two samples by quantifying the amount of shared versus unique genetic features. We observed a correlation between Euclidean and genetic distance across all samples (Supplementary Fig. 8b). Exclusion of mixed CE/NE pairs and stratification into CE only and NE only pairs revealed that only paired CE samples maintained statistical significance upon stratification (*p* = 1.2 × 10^−7^ vs. *p* = 0.078) (Supplementary Fig. 8c).

Given the significance of the pathway-based classification^9^ in defining the transcriptomic clusters, we next examined how genetic and Euclidean distances are associated in different subtypes. In assessing any two samples of the same molecular class, we found that NEU and PPR pairs (NEU to NEU or PPR to PPR) have no correlation between their Euclidean and genetic distances (Fig. 7c). In contrast, the GPM and mitochondrial (MTC) pairs exhibited a statistically significant positive correlation between genetic and Euclidean distance (Fig. 7c).

To understand how genetic and Euclidean distance are related to the two NE phenotypes identified in Fig. 7b, we selected all multiregional pairs that included at least one CE and one NE sample. We found that CE samples paired with a NEU NE sample had a greater genetic distance than CE samples paired with NE samples of the other three pathway-based subtypes, and this trend did not apply to Euclidean distance (Fig. 7d and Supplementary Fig. 8d). This greater genetic distance suggested an increased burden of private mutations in NE NEU samples. In fact, when considering both CE and NE, overall, the samples classified as NEU had the greatest percentage of non-truncal mutations, with 16.34% of all alterations being private (Supplementary Fig. 8e). This disparity between subtypes is driven by samples from the NE region; while there was not a significantly greater proportion of non-truncal mutations in NEU samples from the CE, there was a significant number of non-truncal mutations in NEU samples from the NE (Fisher’s Exact test *p* = 6.13e−27) (Fig. 7e–g and Supplementary Fig. 8f–h).

### Multi-parametric MRI offers biophysical insights to the regional tumor microenvironment and biological signatures of HGG

**Fig. 8:**
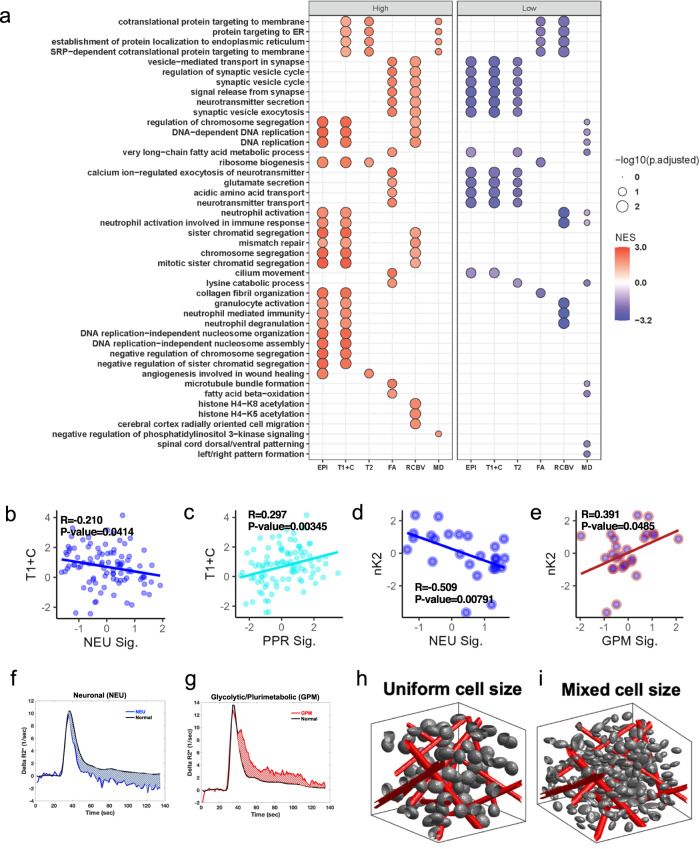
Associations between imaging variables and pathway-based signatures with subsequent phenotypic modeling. **a** Transcriptomic pathway enrichment analysis for samples binned as high vs low for each image feature. Gene Set Enrichment Analysis (GSEA, Kolmogorov–Smirnov-like test as implemented in clusterProfiler1) of supervised differential analysis (Mann–Whitney–Wilcox test) among samples labeled as high vs low for each image feature. Size of the dot represents the adjusted p-value of significant enriched GO BP terms (Benjamini-Hochberg adjustment); color of the dots represents the Normalized Enrichment Score (NES) of the terms. **b**, **c** Scatter plots showing significant correlations between T1 + C and NEU (**b**) or PPR (**c**) signatures across all samples. **d**, **e** Scatter plots showing significant correlations between nK2 and NEU (**d**) or GPM (**e**) in NE samples only (indicated by blue center on data points). **b**–**e** Statistical test: two sample correlation t-test. **f**, **g** Average R2*(t) curves for regions corresponding to a NEU (**f**) and GPM (**g**) sample. R2*(t) is used to derive nK2. **h**, **i** 3D renderings demonstrating conditions illustrative of homogenous uniform cell sizes (**h**) with 0% coefficient of variation and heterogenous mixed cell sizes (**i**) with 6.5% coefficient of variation. **b**–**i** Source data are provided as a Source data file.

To better understand the regional variations in biological phenotypes of HGG, we examined the relationship between spatially matched transcriptomic pathway enrichment and localized MRI features. We binned all samples into high and low groups according to the median for each imaging variable. Differential pathway analysis on transcriptomics profiling revealed that samples with high T1 + C and EPI + C signals were enriched in proliferative pathways, including cell cycle and DNA replication (Fig. 8a, Supplementary Data 4–5). Samples with low T2W signal were enriched with pathways associated with neuron synapses and neurotransmitter release (Fig. 8a, Supplementary Data 5)^47^.

We next examined the relationship between pathway-based classification signatures and imaging metrics. MEMs of imaging features and the categorical variable of pathway-based signature class revealed no significant interactions (Supplementary Data 2). To detect continuous relationships, we then examined all pairs of correlations between imaging features and pathway-based subtype signature values with recorded outputs being Pearson’s correlation, correlation p-value, linear MEM R2, linear MEM effect size (linear model slope), and linear MEM effect size standard error (Supplementary Fig. 9a). Using this combination of outputs, we identified robust relationships between imaging values and pathway-based subtype signatures (see statistical interpretation guide Supplementary Fig. 9b). Each analysis was run on the cohort as a whole and in CE and NE sub-cohorts. On examination of CE and NE distributions of each imaging parameter irrespective of transcriptomic phenotype, several metrics were statistically significantly different with T1 + C, the metric used to designate CE vs NE, reaching the highest level of significance (*p* = 2.21 × 10^−8^) (Supplementary Fig. 10a).

Comprehensive examination of conventional MRI metrics revealed several significant relationships both in the overall cohort (Supplementary Fig. 10b) and in CE specific samples (Supplementary Fig. 11 and Supplementary Data 5). T1 + C and EPI + C were negatively correlated with the NEU signature, but positively correlated with the PPR signature (Fig. 8b, c and Supplementary Fig.12a–e). We confirmed these findings by binning samples into high and low classes based on EPI + C or T1 + C values and noted statistically significant representation of the categorical PPR class for samples with high T1 + C or EPI + C and the categorical NEU class for samples with low T1 + C or EPI + C signal (Supplementary Fig. 12b–e). These findings are consistent with the broader pathway analysis and confirm that T1 + C high signal regions are highly proliferative with significant BBB disruption. Conversely, transcriptionally neuronal areas display less BBB dysfunction. Although the biophysical implications of EPI + C signal are not fully understood, our results indicate that it is predictive of underlying biology including genetics (IDH status) and pathway-based classifications (PPR and NEU). T2W signal demonstrated a positive correlation with the MTC signature and a negative correlation with the NEU signature (Supplementary Fig. 12f, g), suggesting differential water content across transcriptional regions.

rCBV is clinically associated with high-grade pathology and is one of the most thoroughly studied advanced MRI metrics to date^14,15,19^. Across our entire cohort we found some promising rCBV associations, but upon MEM correction these were found to be due to patient effects (Supplementary Fig. 13a, b and Supplementary Fig. 9b). Within the CE region, rCBV positively correlated with NEU and PPR signatures and negatively correlated with MTC even with patient effect correction (Supplementary Fig. 13c, d). Lower rCBV values have been associated with more favorable prognosis^14,15^, and the MTC subtype has separately been linked to more favorable survival outcomes^9^.

### DSC-MRI provides insight into HGG microenvironmental states

We extended the DSC-MRI analysis to include additional metrics with complementary biophysical underpinnings. These include percent signal recovery (PSR), cerebral blood flow (nRCBF/RCBF)^48^, contrast leakage/permeability and subsequent contrast distribution (K2 and nK2) within the extravascular extracellular space, and bolus transit time within the microvasculature (MTT, mean transit time), the macrovasculature (Tmax, time-to-max), or both (RTTP, relative time to peak)^49^. Only nK2 and PSR showed significant differences between the CE and NE samples, but when correlating to pathway-based classifications, we found trends generalizable to all samples and specific to the NE or CE (Supplementary Figs. 14, 15, and Supplementary Data 6). The GPM signature uniquely correlated with Tmax and produced a high effect size on MEM modeling (Supplementary Fig. 16a, b). While Tmax has been primarily used in the assessment of cerebrovascular disease (e.g., stroke), elevated Tmax in the context of the tumor microenvironment reflects slow blood flow through either a collateral network of vessels in parallel or through tortuous microvessels^49,50^. The MTC signature was positively associated with PSR values with a high effect size (Supplementary Fig. 16c, d). Increased PSR in MTC type regions could reflect several possible microenvironmental changes, but in the context of our rCBV finding, a lower microvessel volume is the most probable cause^51^. It is possible that the higher water content with MTC (T2W) was due to intra- or extracellular edema and not due to a higher blood volume in these regions. RCBF and nRCBF showed one significant association each, but these did not meet full criteria for significance (Supplementary Figs. 16e–h and  9).

Examination of contrast distribution and its association with biological signatures revealed several notable associations. Both K2 and K2 normalized against normal white matter (nK2) represent leakage factors which give an indication of blood–brain barrier permeability; however, these measurements are affected by extracellular contrast distribution and thus also convey information about the cellular fraction and cell size heterogeneity^52,53^. Within CE regions, K2 values were positively correlated with PPR signatures but were a false positive on MEM correction (Supplementary Fig. 17a, b). Within NE regions, K2 correlations with the NEU signature demonstrated an effect size of > 0.4, the highest effect we detected amongst all significant MRI-signature models (Supplementary Fig. 17c, d). The GPM signature within NE exhibited an opposite relationship with K2 (compared to NEU signatures), which did not reach statistical significance (Supplementary Data 6). In the NE, nK2 demonstrated a strong negative correlation with the NEU signature and a strong positive correlation with the GPM signature (Fig. 8d, e, Supplementary Fig. 17e). Additionally, the relationship between nK2 and NEU showed a positive correlation in the CE region with a smaller effect size (Supplementary Fig. 17f, g). From these results, we surmise that in the NE region the GPM and NEU phenotypes identified in Fig. 7b exist within diverging biophysical environments. The combination of NEU signature associations (T1 + C, T2W, and nK2) demonstrates that tumor cells with NEU signature reside in a microenvironment enriched in myelin with low water content, homogenous in cell size, and relatively preserved blood–brain barrier integrity.

Our data shows that GPM samples show greater magnitude of T2*W relaxivity changes (delta-R2*) relative to the normal brain, and NEU demonstrates markedly diminished relaxivity changes relative to normal brain (Fig. 8f, g). Thus, to identify the specific microenvironmental underpinnings of this effect, we performed simulations using our previously published digital reference object (DRO) model^54–58^. The DRO model suggests that cell size heterogeneity and overall cell size are the drivers of changes in K2 and nK2 measurements (Fig. 8g–i, Supplementary Fig. 17h). To confirm this, we interrogated the specific composition of the tumor microenvironment (TME) using CIBERSORTx to estimate cell fractions. Transcriptomic analysis of GPM and NEU samples in the NE revealed that GPM samples had a higher proportion of immune cells compared to NEU samples which were more enriched in neurons (Supplementary Fig. 18). Together these results suggest that the microenvironment of GPM subtype in the NE region are made up of overall larger and/or more heterogeneous cell sizes, which could be explained by the enrichment of immune cell signature identified by transcriptomic analysis. In comparison, the nK2 signature of the NEU subtype in the NE region supports a smaller and/or more homogenously sized cell population (Supplementary Fig. 18).

## Discussion

Despite the importance of the NE region in clinical recurrence^59–61^ the molecular and phenotypic features of HGG cells in NE tumor regions remain inadequately understood. In this study, we have integrated multi-parametric MRI with spatially matched molecular sequencing data from HGG to characterize biologically distinct regions comprising invasive unresectable NE tumor to better inform clinical management in diagnosis, prognosis, and treatment. Our results demonstrate an expanded role of advanced MRI to inform regional biology for clinical decision-making.

Regardless of IDH status, we found that NE tumor regions harbored the highest proportion of private mutations, which reflects an increased development of regional genomic complexity in infiltrative tumor and implies that mutational burden in HGG is subject to sample location. We thus propose that both regional genomic instability and tumor infiltration occur early in gliomagenesis^62,63^. The multiregional genomic profiling of our IDH wild-type HGG cohort reveals that *EGFR* and *NF1* somatic alterations occur as mutually exclusive events in 98.7% of tumors. However, we also resolved rare low allele frequency co-alterations of *EGFR* and *NF1* within the NE region. On external validation of other cohorts, we find this co-occurrence enriched in recurrent tumors, thus pointing to the early emergence of *NF1* inactivation in the NE regions^64,65^ and suggesting that these alterations are important in shaping recurrent tumor. Both *NF1* loss^65^ and *EGFR* alterations^66^ have been shown to impact the recruitment of myeloid cells into the tumor microenvironment, thus *NF1* and *EGFR* co-altered populations may play a cooperative role in shaping the cell composition of infiltrative tumor and enabling recurrence. Further single cell studies are required to clarify if *NF1* and *EGFR* co-alteration at recurrence occurs within a single cell. Indeed, our proposed models of molecular evolution inferred from NE and CE regions of IDH wild-type tumors highlight EGFR amplification and NF1 inactivation as parallel temporal events in either gliomagenesis or progression counterbalanced by a dichotomous temporal role for the PI3K pathway. These models clarify expected evolutionary patterns across the otherwise temporal complexity of well-studied canonical driver alterations, further supporting the potential interplay of EGFR and NF1 alterations driving molecular progression at the invasive front of tumor. Moreover, we detailed the spatially unique acquisition of multiple distinct *EGFR* alterations giving rise to intratumoral *EGFR* mosaicism, a challenge in the implementation of EGFR directed therapies^67^. Here, we determined that the integration of localized imaging data enables phenotypic clarity with a translational potential in the face of extensive genomic heterogeneity. Our data attributes decreased regional mean diffusivity (MD) in NE tumor regions to co-occurrence of *EGFR* amplification and *CDKN2A* homozygous deletion, which reflects a high tumor cell density in these regions^2^. Correlating MD to NE tumor regions harboring *EGFR* amplification/*CDKN2A* deletion offers a means to assess regional cell proliferation, and identifying foci of cell proliferation enables a potential translational capability to implement targeted localized therapies.

While the NE region harbors a greater burden of private mutations compared to the CE region, we found no correlation between Euclidean and genetic distance (i.e., accumulated mutations). This may likely be explained by divergent regional patterns of tumor expansion that are not accounted for under Euclidean distance measurements, which solely reflect the linear distance between two points. We postulate that the bulk CE is an outward expansion driven by cell proliferation that is more likely to fit into a linear distance model than the NE, where tumor cells have more potential to take a non-linear path during brain parenchymal invasion. This renders Euclidean distance to be a likely underestimate of the actual distance traveled for invasive NE tumor cells.

Correlation between Euclidean and genetic distance varies when separated by pathway-based classification. For example, GPM subpopulations display a correlation between Euclidean and genetic distance, whereas NEU subpopulations, which harbor more private mutations than the GPM, do not have a correlation. While we find both GPM and NEU predominant populations in the NE, these distance correlations may reflect divergent patterns of cell invasion across subtype. Prior reports have associated tumors harboring a greater burden of private mutations with a distant recurrence pattern relative to tumors with proximal recurrence^68^. We thus hypothesize invasive NEU-predominant populations may travel more distant paths that are less well represented by Euclidean distance measurements than a GPM subtype pattern of cell invasion. This phenotypic dichotomy between GPM and NEU populations supports the growing body of evidence that invasive GBM cells either take on a neuronal phenotype for active invasion or a more metabolic phenotype involving interaction with astrocytes, other glial cells, and infiltrating immune cells^69,70^. Our study extends this observation to include correlations with advanced imaging features that can be used clinically to discern these two phenotypes, specifically through nK2 on DSC-MRI. However, we recognize that imaging informs a phenotype, which could receive additional contribution from further biological associations not yet identified.

Still, our study paves the way for advanced imaging to take a more expansive role in both basic science and clinical evaluation. Advanced MRI parameters may allow us to identify the microenvironmental phenotypes associated with molecularly defined subpopulations in the NE, including metrics that have been less robustly explored (i.e., EPI + C, nK2). Thus, integrated multi-omic analysis using molecular and advanced imaging profiling holds clinical promise for identifying tumor characterized by distinct invasive biology or with potential therapeutic vulnerabilities in the critical NE region, for use in pre-surgical planning and personalized treatment regimens.

## Methods

### Multiregional glioma sample cohort

Our research complies with all relevant ethical regulations. We received Institutional Review Board (IRB) approval from both Barrow Neurological Institute (BNI) and Mayo Clinic. The IRB protocols allowed for data sharing across both institutes. Multiregional tumor frozen samples (*n* = 339) and matched peripheral blood samples (*n* = 63) were collected from 74 glioma patients. DNA and RNA samples have been profiled by Whole Exome Sequencing (WES) and RNA sequencing (RNAseq). Clinical, MRI contrast enhancing, and sequencing information are provided in Supplementary Data 1. All sample sizes for statistical analyses are summarized in Supplementary Data 7.

### Acquisition and processing of clinical MRI

#### Patient recruitment and surgical biopsies

We recruited patients with clinically suspected high-grade glioma undergoing pre-operative stereotactic MRI for surgical resection as previously described^71^. Histologic diagnosis and WHO grade were confirmed by two board-certified neuropathologists ( J.M.E., K.D.). All samples from an individual tumor were uniformly annotated based on histologic diagnosis and WHO grade. Patients were recruited from BNI and Mayo Clinic through IRB approved protocols at each institution. The IRB at Mayo Clinic served as the overarching protocol which coordinated tissue and image data transfer from BNI to Mayo Clinic. Informed consent from each subject was obtained prior to enrollment. All data collection and protocol procedures were carried out following the approved guidelines and regulations outlined in the Mayo Clinic and BNI IRB protocols. Neurosurgeons used pre-operative conventional MRI, including T1-Weighted contrast-enhanced (T1 + C) and T2-Weighted sequences (T2W), to guide multiple stereotactic biopsies as previously described^71–73^. In short, each neurosurgeon collected an average of 4–5 tissue specimens from each tumor using stereotactic surgical localization, following the smallest possible diameter craniotomies to minimize brain shift. Neurosurgeons selected targets generally separated by at least 1 cm from both enhancing core (ENH) and non-enhancing T2/FLAIR abnormality in pseudorandom fashion, and recorded biopsy locations via screen capture to allow subsequent coregistration with multiparametric MRI datasets. Typical volumes of biopsy samples were approximately targeted to be 0.125 cc.

#### Conventional MRI and general acquisition conditions

We performed all imaging at 3 T field strength (Sigma HDx; GE-Healthcare Waukesha Milwaukee; Ingenia, Philips Healthcare, Best, Netherlands; Magnetome Skyra; Siemens Healthcare, Erlangen Germany) generally within 1-3 days prior to stereotactic surgery. Conventional MRI included standard pre- and post-contrast T1-Weighted (T1W , T1 + C, respectively) and pre-contrast T2-Weighted (T2W) sequences as previously described^15^. T1W and T1+C images were acquired using spoiled gradient recalled-echo inversion-recovery prepped (SPGR-IR prepped) (TI/TR/TE = 300/6.8/2.8 ms; matrix = 320 × 224; FOV = 26 cm; thickness = 2 mm). T2W images were acquired using fast-spin-echo (FSE) (TR/TE = 5133/78 ms; matrix = 320 × 192; FOV = 26 cm; thickness = 2 mm). T1 + C images were acquired after completion of dynamic susceptibility contrast (DSC-MRI) as detailed below. Gadolinium based contrast agent (GBCA) was gadobenate dimeglumine for patients recruited at Barrow Neurological Institute (BNI) and either gadobenate dimeglumine or gadobutrol for patients recruited at Mayo Clinic in Arizona (MCA).

#### Diffusion tensor imaging (DTI)

DTI imaging was performed using Spin-Echo Echo-planar imaging (EPI) (TR/TE 10,000/85.2 ms, matrix 256 × 256; FOV 30 cm, 3 mm slice, 30 directions, ASSET, B = 0,1000), and whole-brain maps of mean diffusivity (MD) and fractional anisotrophy (FA) were generated based on previously published methods^71,73,74^.The original DTI image DICOM files were converted to a FSL recognized NIfTI file format, using MRIConvert (v2.1.0), before processing in FSL from semi-automated script. DTI parametric maps were calculated using FSL 5.0 (http://fsl.fmrib.ox.ac.uk/fsl/fslwiki/), to generate whole-brain maps of mean diffusivity (MD) and fractional anisotrophy (FA) based on previously published methods^74^.

#### Dynamic susceptibility contrast MRI (DSC-MRI) acquisition for relative cerebral blood volume (rCBV) calculation

For all patients (BNI and MCA), we administered a preload dose (PLD) of GBCA (0.1 mmol/kg) to minimize T1W leakage effects prior to DSC-MRI acquisition for measurement of relative cerebral blood volume (rCBV). After PLD, we employed a Gradient-echo (GE) EPI (TR/TE/flip angle = 1500 ms/20 ms/60°, matrix 128 × 128, thickness 5 mm) DSC MRI acquisition for 3 min. At approximately 30 s after the start of the DSC-MRI sequence, we administered a second GBCA bolus injection (0.05 mmol/kg at BNI; 0.1 mmol/kg at MCA), which was used to calculate rCBV maps for all patients. Based on prior studies, measurement of rCBV is considered optimal following administration of a PLD, particularly when employing sequences with moderate flip angles^75,76^.

#### Dynamic susceptibility contrast MRI (DSC-MRI) acquisition for non-rCBV metrics

For patients recruited at MCA, we also employed a DSC-MRI acquisition during the contrast bolus administration of the PLD. Prior to PLD injection, we employed a Gradient-echo (GE) EPI (TR/TE/flip angle = 1500 ms/20–30 ms/30°, matrix 128 × 128, thickness 5 mm) DSC MRI acquisition for 3 min. At approximately 30 s after the start of the DSC sequence, we administered the 0.1 mmol/kg i.v. bolus injection of GBCA, which was used to calculate non-rCBV-related DSC-MRI metrics (e.g., nK2, PSR, MTT). Given that many of these metrics relate to measurement of contrast leakage effects, optimal measurement of these metrics should be performed prior to preload dose administration^51^.

#### Acquisition of EPI + C images

The initial source volume of images from the DSC-MRI sequence, acquired ~6 min following PLD administration (i.e., the sequence used to calculate rCBV), was used to represent the EPI + C map, as previously described^71–73^. This image volume contained negative contrast enhancement (i.e., susceptibility effects from the PLD administration). At ~6 min after the time of GBCA injection, which allows for distribution of the agent through the extravascular extracellular space (EES), the T2*W signal loss on EPI + C provides information about tissue cell density and cell size heterogeneity but may also receive contributions from T1W leakage effects^52,72^.

#### Post-processing analysis of DSC-MRI for rCBV and non-rCBV metrics

We generated whole brain parametric maps for rCBV and non-rCBV metrics, using the respective DSC-MRI acquisitions and the post-processing pipelines within the IB Neuro/IB RadTech (v21.12, Imaging Biometrics, LLC) interface. We used leakage correction modeling for calculation of rCBV and relevant non-rCBV metrics (e.g., MTT, RCB). For normalized metrics including rCBV, we used measurements from regions of interest (ROIs) from contralateral normal appearing white matter as previously described^16,72,77^.

#### Normalization of qualitative imaging sequences

We performed N4 normalization of all non-quantitative maps, including T1 + C, T2W, and EPI + C images. The python library (v3.6.2) from SimpleITK (v1.0.1) was employed for all steps of normalization. Image denoising was first performed using sitk.CurvatureFlow, followed by N4 bias correction using sitk.N4BiasFieldCorrection. Intensity normalization was subsequently performed using a brain mask generated with sitk.MaskImageFilter for each image. Normalization was performed based on median signal intensities from a whole brain mask generated from each imaging sequence for each individual patient.

#### Image coregistration

We coregistered all datasets to the relatively high-quality DTI B0 anatomical image volume using tools from ITK (www.itk.org) and IB Delta Suite (v21.12, Imaging Biometrics, LLC) as previously described^71–73^. This offered the additional advantage of minimizing potential distortion errors (from data resampling) that could preferentially impact the mathematically sensitive DTI metrics. Ultimately, the coregistered data exhibited in plane voxel resolution of ~1.17 mm (256 × 256 matrix) and slice thickness of 3 mm.

#### Region of interest (ROI) generation, CE and NE annotation, and image feature extraction

We referenced the stereotactic locations recorded intraoperatively for each biopsy specimen to generate spatially matched regions of interest (ROIs) measuring 8 × 8 × 1 voxels (9.6 × 9.6 × 3 mm). This resulted in regions of interest (ROI) with volumes ~0.28 cc. A board-certified neuroradiologist (L.S.H.) visually inspected all ROIs to ensure accuracy and annotated each biopsy specimen location as either contrast-enhancing (CE) or non-enhancing (NE) rim^71–73^. From each ROI, we employed our in-house image analysis pipeline to extract mean values from each ROI, for each imaging technique map, for correlative analysis. See Supplementary Fig. 2 for image processing workflow.

#### Image quality assessment

Each biopsy location was also assessed for potential image artifacts that could obscure signal intensity values, including artifacts at the interface of bone or air (e.g., floor of the anterior skull base, middle cranial fossa superior to the mastoid air cells), as well as metallic artifacts from prior surgical instrumentation (e.g., craniectomy plate/screws), which were excluded from analysis. We also  identified biopsy locations which were recorded centrally within locations that were expected to yield no viable tissue (e.g., resection cavity and/or central necrosis), for exclusion. We excluded those biopsies meeting these criteria from correlative analysis with imaging techniques. We also excluded biopsy samples in close proximity to large surface vessels (e.g., middle cerebral artery branches), from correlative analysis with DSC-MRI-based image features (e.g., rCBV maps), which are susceptible to artifacts from these large vessels. Biopsy samples at the margin of T1+C enhancement and necrosis, which did not meet exclusion criteria, were also annotated (Supplementary Data 1).

### WES and somatic mutations

WES was performed on 328 multiregional tumor samples and their paired blood DNA samples (*n* = 63) from 72 glioma patients. The glioma cohort included 196 and 123 MRI contrast enhancing and non-enhancing samples, respectively.

DNA/RNA were extracted from frozen surgical specimens. WES was performed at Mayo Clinic (Rochester, MN), Novogene or Translational Genomics Institute (TGen; Phoenix, AZ) using the SureSelect (Agilent) (Mayo Clinic, Novogene) or Strexome V2 capture kits (TGen). WES of patient germline was also performed. Raw sequencing data processing and quality control were performed as previously described^43^. Briefly, sequencing reads were aligned to GRCh37/hg19 human reference genome using Burrows-Wheeler Aligner^78^, and further processed by GATK3^79^ to remove low mapping quality reads and to re-align around the indels. To confirm that multiregional tumor and blood samples derived from the same patient, we performed the fingerprint analysis using NGSCheckMate^80^, a model-based method evaluating the correlation between the variant allele fractions at known SNP sites.

Somatic SNVs and indels were identified by integrating the results from 6 algorithms for variants calling: Freebayes (arXiv:1207.3907) MuTect2^81^, TNhaplotyper^82^, TNscope^82^, TNsnv^82^, and VarScan2^83^.

For tumor samples without matched blood DNA, the somatic status of called mutations was assessed using a virtual normal panel from a set of 433 public samples from healthy, unrelated individuals sequenced to high depth in the context of the 1000 Genomes Project^84^. Putative false positive calls have been removed by applying an in-house filtering pipeline previously described^43^. For each patient, the multiregional mutation profile was built by combining the genetic variants identified in the multiple spatial biopsies from the single case, which have been annotated as private (exclusively occurring in one sample), shared (occurring in two or more samples, but not in all samples) and truncal (occurring in all samples). To validate the sharing profile of mutations, the nucleotide at each mutant position was re-called from the raw sequences within all the multiple samples from a single patient. Using this iterative approach, false negative calls have been retrieved by identifying mutant reads at genetic positions that had been mis-called as wild type.

Somatic variants were annotated using AnnoVar^85^, which aggregates information from genomic and protein resources with cancer and non-cancer variant databases. Variants reported in the non-cancer databases with a minor allele frequency ≥0.05 were classified as germline polymorphisms and excluded.

The functional effect of missense SNVs and in-frame indels was determined by an ensemble of multiple algorithms^86^. Variants predicted as damaging by two or more algorithms were classified as pathogenic mutations.

### DNA copy number

Somatic copy number and tumor purity were estimated from WES by PureCN^87^. GISTIC2^88^ analysis was then applied to integrate results from individual patients and identify genomic regions recurrently amplified or deleted in glioma samples.

### Gene fusion and EGFR variant identification

RNAseq reads were analyzed to identify fusion transcripts using STARfusion (bioRxiv, 120295). Predicted gene fusions were annotated by AGFusion (bioRxiv, 080903), and in-frame chimeras supported by more than 10 reads were selected.

EGFR gene variants (including exons 2-7 deletion EGFRvIII) have been detected from RNAseq reads by CTAT-Splicing (https://github.com/NCIP/CTAT-SPLICING/).

### Phylogenetic analysis and glioma evolution modeling

The intratumor evolution of glioma was predicted by inferring the genetic trajectories across the multi-region specimens within each single patient. The allele frequencies of genetic alterations (including mutations and copy number variations) were compared using the clustering approach implemented in PhyC^30^ to reconstruct a patient-based tree of geographical glioma evolution, reflecting the genetic distances among the multiregional samples.

The evolutionary trajectories across multiple patients have been tested by applying a statistical model implemented in REVOLVER^44^. Multi-region sequencing data from the IDH wild-type cohort were jointly analyzed using the transfer machine-learning approach (TL) to predict hidden evolutionary patterns. The analysis was restricted to a subset of cases in which two or more truncal driver alterations were identified (34 patients). The structural correlation across patients was measured extracting the phylogenetic trajectories of driver alterations from the patients’ trees. A supervised clustering approach has been applied to stratify the patients that share tumor-initiating trajectories of genetic driver alterations, leading to the prediction of four main models of glioma evolution.

### Transcriptomic analysis

Raw reads were aligned to a Human genome (UCSC genome assembly GRCh37) using STAR (v. 2.7.0b)^89^, and the expression was quantified at gene level using featureCounts (v. 1.6.3)^90^, a count-based estimation algorithm. Downstream analysis was performed in the R statistical environment as described below. Raw data from different batches were normalized separately according to sample-specific GC content differences as described in EDAseq R package (v. 2.22.0)^91^. The batch adjustment was performed using a negative binomial regression as implemented in Combat-seq function of sva R package^92^.

Differential expression analysis was performed using EdgeR R package (v. 3.30.3)^93^. Genes with an adjusted *P*-value (Benjamini & Hochberg correction) less than or equal to 0.01 and absolute log2 foldchange greater than or equal to 1 were considered significantly differentially expressed (DEGs). Gene set enrichment analysis (GSEA)^94^ and DEG hypergeometric over-representation test for Biological Processes were computed using the clusterProfiler R package (v. 3.3.6)^95^. The full list of genes ranked according to the Mann–Whitney–Wilcox statistic was considered as input for GSEA.

To classify tumor samples according to Wang et al.^65^ and pathway-based classification^9^, a single sample Gene Set Test based on the Mann–Whitney–Wilcox statistic (mww-GST)^96^ was used. Each tumor sample was classified in a distinct subtype based on the highest significant score (logit(NES) greater than 0.40 and *p*-value less than 0.05).

The tumor microenvironment deconvolution was computed using a curated collection of immune-related and cell type specific signatures retrieved from scTHI R package^97^ and Molecular Signatures Database (MSigDB)^94,98^ (*n* = 389), respectively. For each signature the deconvolution score was calculated using an approach based on the single sample Gene Set Test as previously described^96^. Only the signatures with a score greater than 0.58 and p-adjusted less than 0.01 in at least the 20% of samples have been considered for downstream analysis.

Tumor microenvironment cell fractions were inferred from bulk RNAseq expression matrix using CIBERSORTx webserver^99^.

The single-cell RNAseq signature matrix used as reference to estimate non-tumor cell proportions was created from Darmanis et al.^100^, using the ‘Create Signature Matrix’ module as described in CIBERSORTx webserver manual. In order to compare tumor and TME abundance, tumor subtype enrichment scores were scaled in a range from 0 to 1. Then, both tumor and non-tumor cell proportions were scaled according to the tumor purity inferred from WES.

### Genomic associations with imaging variables

To account for patient effects across samples, MEM was conducted using the lme4 package in R. For all models, patient identity was modeled as a categorical random effect, and for each examined genetic context, the standard MRI features (T2W, rCBV, MD, FA, EPI + C) were assessed in all samples, CE samples, and NE samples for a total of 15 models per examined genotype. The fixed effects for each model were specified as categorical variables in the specified formulas with their corresponding figures (Table 1). From these models, the significance of each term, the percent variance explained by each term, MEM corrected means/ variance for each genotype, and pairwise comparison of MEM corrected means for each genotype were generated.

### Analysis of genetic and euclidean distances

Within this cohort, several samples were procured per patient, and these samples were related in a pairwise fashion within each patient using genetic and euclidean distances. Genetic distances were computed as 1 - Jaccard index on the genetic alteration patterns (including mutations and CNV). Euclidean distances were calculated from spatially resolved MRI coordinates using the following Eq. 1.

1 $$d\left(p,q\right)=\sqrt{{({p}_{1}-{q}_{1})}^{2}+{\left({p}_{2}-{q}_{2}\right)}^{2}+{\left({p}_{3}-{q}_{3}\right)}^{2}}$$

Overall correlation between genetic and euclidean distance was assessed using the entire cohort. Following this samples pairs that were both in the CE region or both in the NE region were reassessed to derive the contrast region-specific correlation. For the subset of samples that had transcriptomic data and therefore an assigned pathway-based classification, samples of the same class (ex. NEU-NEU) were then compared to understand how biological phenotype influences the genetic-euclidean correlation.

Finally, to mirror the two NE region phenotypes found on transcriptomic analysis, we selected all sample pairs with assigned pathway-based classification and that had one sample in CE and one in NE. We then examined the average genetic and euclidean distance between the CE samples to each pathway-based classification.

### Associations between imaging features and pathway-based classification signatures

As outlined in Garofano et al., samples were assigned a pathway-based classification based on four transcriptomic signature scores for each class^9^. For this analysis, the continuous pathway-based signature scores were used instead of the categorical classification. To broadly assess the relationships between pathway-based classifications (GPM = glycolytic/plurimetabolic, MTC = MTC, NEU = NEU, PPR = proliferative/progenitor), traditional MRI features (T2W, RCBV, MD, FA, T1 + C, EPI + C), and advanced MRI features (k2, MTT, nK2, nRCBV, sRCBV), correlation maps were created using GGally in R. MEMs were again used to correct for patient effects with patient specified as a random effect, pathway-based signature specified as a fixed effect, and imaging variable specified as the outcome variable. Both pathway-based signatures and imaging variables were scaled via z-score prior to modeling.

### Visualization

Heatmaps were generated using ComplexHeatmap in R. Bar, scatter, and boxplots were created using ggplot and GGally. Conceptual Schematics were made with Biorender.

### MRI time course and digital reference object modeling for nK2

To evaluate the bases of nK2 associations with GPM and NEU correlations, R2* time curves were plotted for regions spatially matched to NEU or GPM biopsy samples, and normal control curves were plotted using normal appearing white matter adjacent to the frontal horns, within the parietal corona radiata, and the global whole brain non-enhancing voxels for each patient. The measurement of K2 was derived from Boxerman et al. as shown below^53^.

2 $$\Delta {{{{{\rm{R}}}}}}2 * \left(t\right) \, \approx \, {K}_{1}\Delta {{{{{\rm{R}}}}}}2 * \left(t\right)-{K}_{2}{\int }_{0}^{t}\Delta {{{{{\rm{R}}}}}}2 * \left({t}^{{\prime} }\right){{{{{\rm{d}}}}}}{{{{{{\rm{t}}}}}}}^{{\prime} }$$

3 $$\Delta {{{{{\rm{R}}}}}}2 * \left(t\right)=-\left(\frac{1}{{{{{{\rm{TE}}}}}}}\right){{{{\mathrm{ln}}}}}\left(\frac{{{{{{\rm{S}}}}}}\left({{{{{\rm{t}}}}}}\right)}{{{{{{{\rm{S}}}}}}}_{{{{{{\rm{o}}}}}}}}\right)$$

R2*(t) reflects the whole brain average (R2*) in all non-enhancing voxels, and K2 reflects contrast leakage. This is estimated from the post-bolus segment (tail of the curve) of R2* and is proportional to the degree of deviation of the tumor voxel from the whole brain non-enhancing voxels. Normalized K2 (nK2) is derived from the K2 of each voxel (including tumor region of interest) after dividing by the K2 of normal appearing white matter regions contralateral to the identified tumor region.

Digital reference object (DRO) modeling was performed to simulate R2* signal changes with changing cell size and cell size variation as described in Semmineh et al.^56^. The DRO model computes MRI signals for realistic 3D tissue structures modeled using ellipsoids (cells) packed around randomly oriented cylinders (vessels) and accounts for static magnetic field strength, intercompartment susceptibility differences, the water proton diffusion coefficient, and pulse sequence parameters. To ensure that the simulated signals accurately represented the magnitude and distribution of contrast agent induced T1 and T2* changes within typical clinical data, we chose model parameters so that the distribution of percentage signal recovery and the mean SD of signal intensities across the DRO matched those in the patient training dataset. The simulation results presented here represent data from two DROs, the first based on tissue structures with homogenous cellular features designed using ellipsoid with identical aspect ratio and diameters (coefficient of variation = 0), and the second with heterogeneous aspect ratio and diameters (coefficient of variation = 6.5%).

### Reporting summary

Further information on research design is available in the Nature Portfolio Reporting Summary linked to this article.

### Supplementary information

Supplementary Information

Peer Review File

Reporting Summary

Description of additional supplementary files

Supplementary Data 1

Supplementary Data 2

Supplementary Data 3

Supplementary Data 4

Supplementary Data 5

Supplementary Data 6

Supplementary Data 7

### Source data

Source Data

## Data Availability

All datasets analyzed in the current study, including whole exome sequencing and RNA sequencing, are publicly accessible, with the Synapse (https://www.synapse.org/#!Synapse:syn52256644). The publicly available bulk RNA-seq data for TCGA-GBM were obtained from the UCSC Xena browser [https://gdc-hub.s3.us-east-1.amazonaws.com/download/TCGA-GBM.htseq_counts.tsv.gz]. The remaining data are available within the Article, Supplementary Information or Source data file. The input data for the imaging analysis can be found at https://github.com/HuLiLab/Multi-Regional-GBM-Imaging-and-Genetics Source data are provided with this paper.

## Code availability

Code for the imaging analysis can be found at https://github.com/HuLiLab/Multi-Regional-GBM-Imaging-and-Genetics.
