# Supplementary material for: Integrated molecular and multiparametric MRI mapping of high-grade glioma identifies regional biologic signatures
Source: Nat Commun. 2023 Sep 28;14:6066. doi: 10.1038/s41467-023-41559-1 (PMC10539500; doi:10.1038/s41467-023-41559-1)
Supplement: Supplementary file 1 — Supplementary Information [file 41467_2023_41559_MOESM1_ESM.pdf]

Supplementary Figure 1.

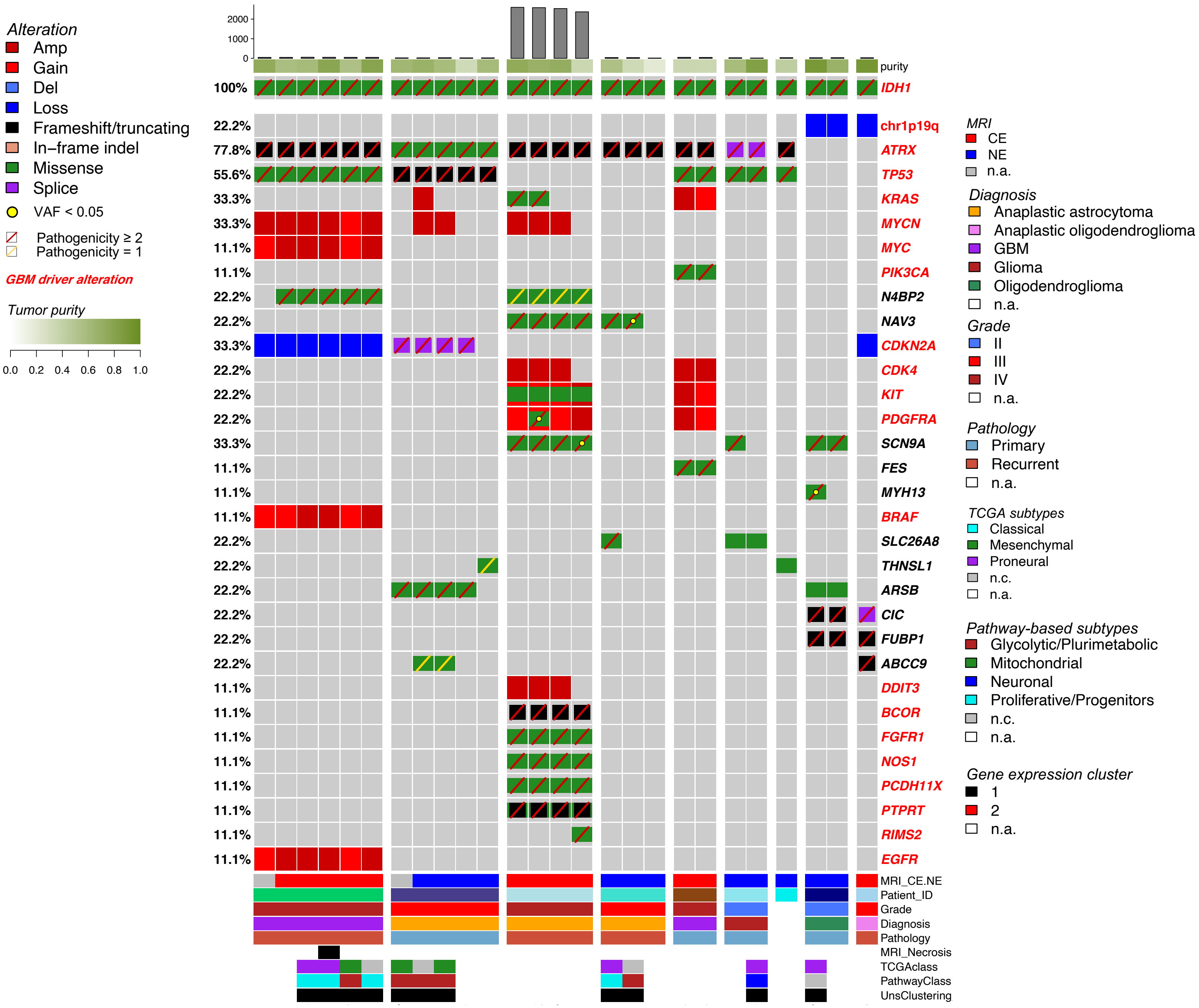

Supplementary Figure 1. Overview of somatic alterations in additional IDH-mutant cases that not include both CE and NE sampling. Mutation load and tumor purity are reported in the top barplot and heatmap track, respectively. Clinical annotation and gene expression classification are indicated in the bottom tracks. Gene alteration frequency in the patient cohort is indicated as percentage on the left, known with driver mutations highlight in red.

Supplementary Figure 2.

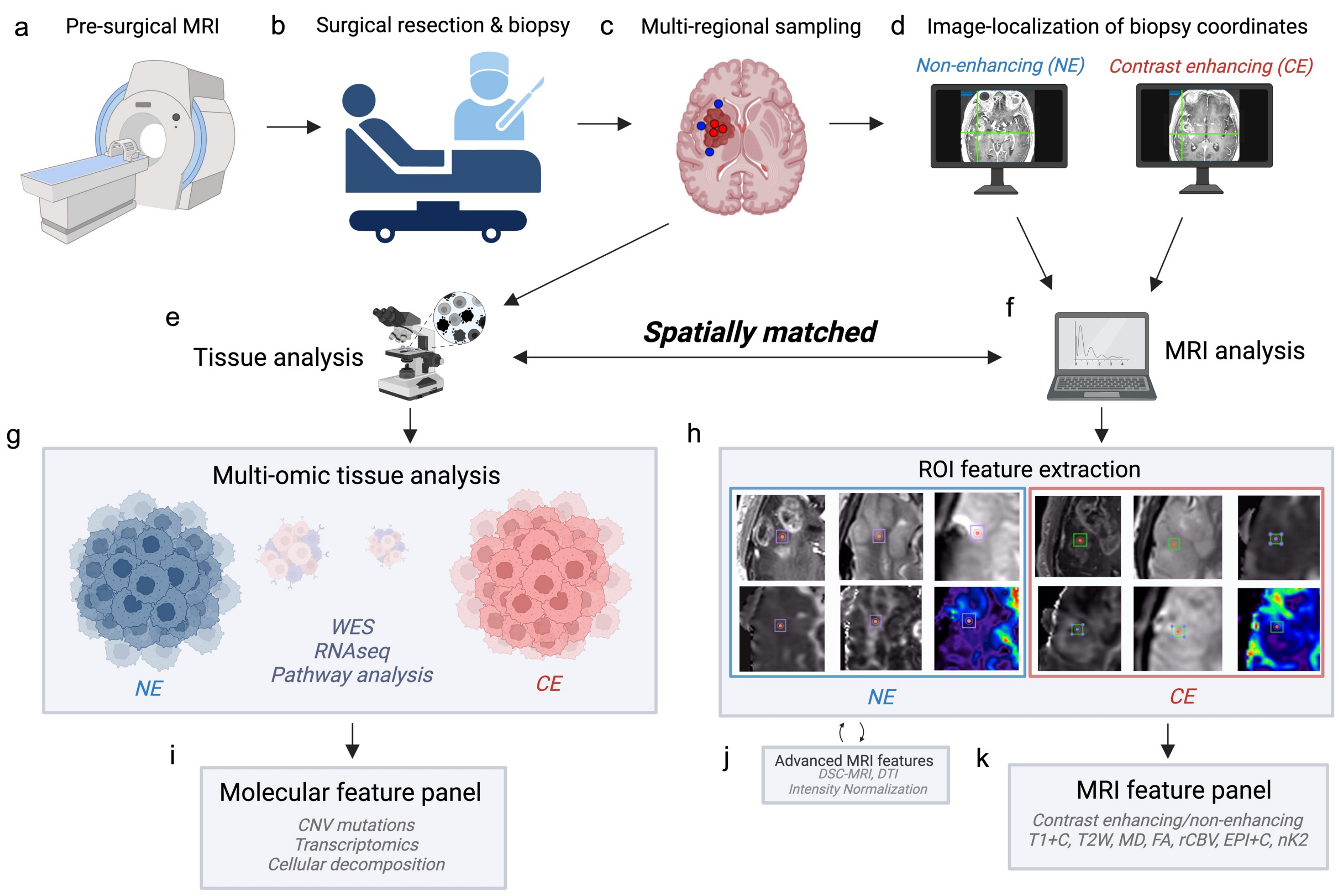

Supplementary Figure 2. Workflow for spatial matching between image-localized biopsy analysis and corresponding regional MRI feature extraction. (a) Each patient undergoes pre-surgical MRI, including stereotactic MRI and advanced multi-parametric sequences. (b) As part of subsequent surgical resection and biopsy, the stereotactic MRI is imported into the clinical neuronavigational platform in the operative room suite, which guides (c) multi-regional tissue sampling from both contrast-enhancing (CE) and non-enhancing (NE) regions throughout each tumor. (d) For each biopsy sample, we record the image-localization of the stereotactic biopsy coordinates. Each biopsy sample undergoes (e) tissue analysis with multi-omic sequencing including (g) whole exome sequencing (WES), RNA sequencing (RNAseq), and pathway analysis. In parallel, (f) spatially matched MRI analysis involves (h) local feature extraction of multi-parametric from the Regions of Interest (ROI) that are coregistered to each set of corresponding biopsy coordinates from (d). Multi-parametric MRI features include those generated from (j) Advanced MRI analysis of Dynamic Susceptibility Contrast (DSC)-MRI and Diffusion Tensor Imaging (DTI). This results in spatial matching between molecular feature panels (i) and the MRI feature panels (k) for each multiregional biopsy sample. CNV = copy number variants; T1+C = T1-Weighted post-contrast signal; T2W = T2-Weigthed signal; MD = Mean Diffusivity; FA = Fractional Anisotropy; rCBV = relative cerebral blood volume; EPI+C = post-contrast T2*-Weighted signal; nK2 = normalized K2.

Supplementary Figure 3.

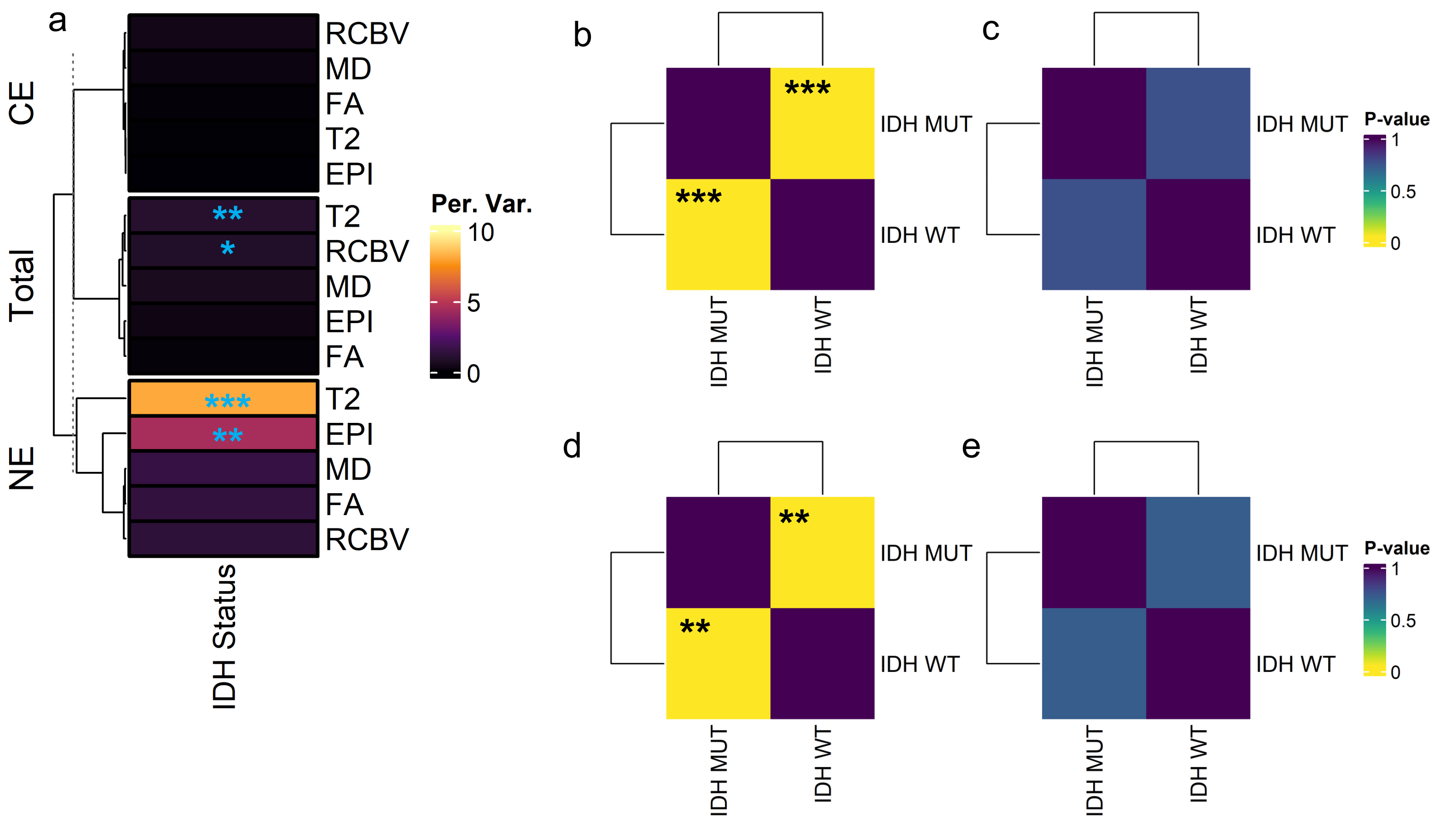

Supplementary Figure 3. a. The percent variance attributed to IDH status (x-axis) in MEMs for each imaging variable (y-axis) separated by region. *p-value < 0.1 **p-value<0.05 ***p-value<0.001 b-e. MEM corrected pairwise p-values for IDH genotypes and their effect on EPI+C in the NE (b) and CE (c) regions and for their effect on T2W in the NE (d) and CE (e). b-e. Statistical test: Two-sided t-test with Tukey correction. a-e. Source data are provided as a Source Data file.

Supplementary Figure 4.

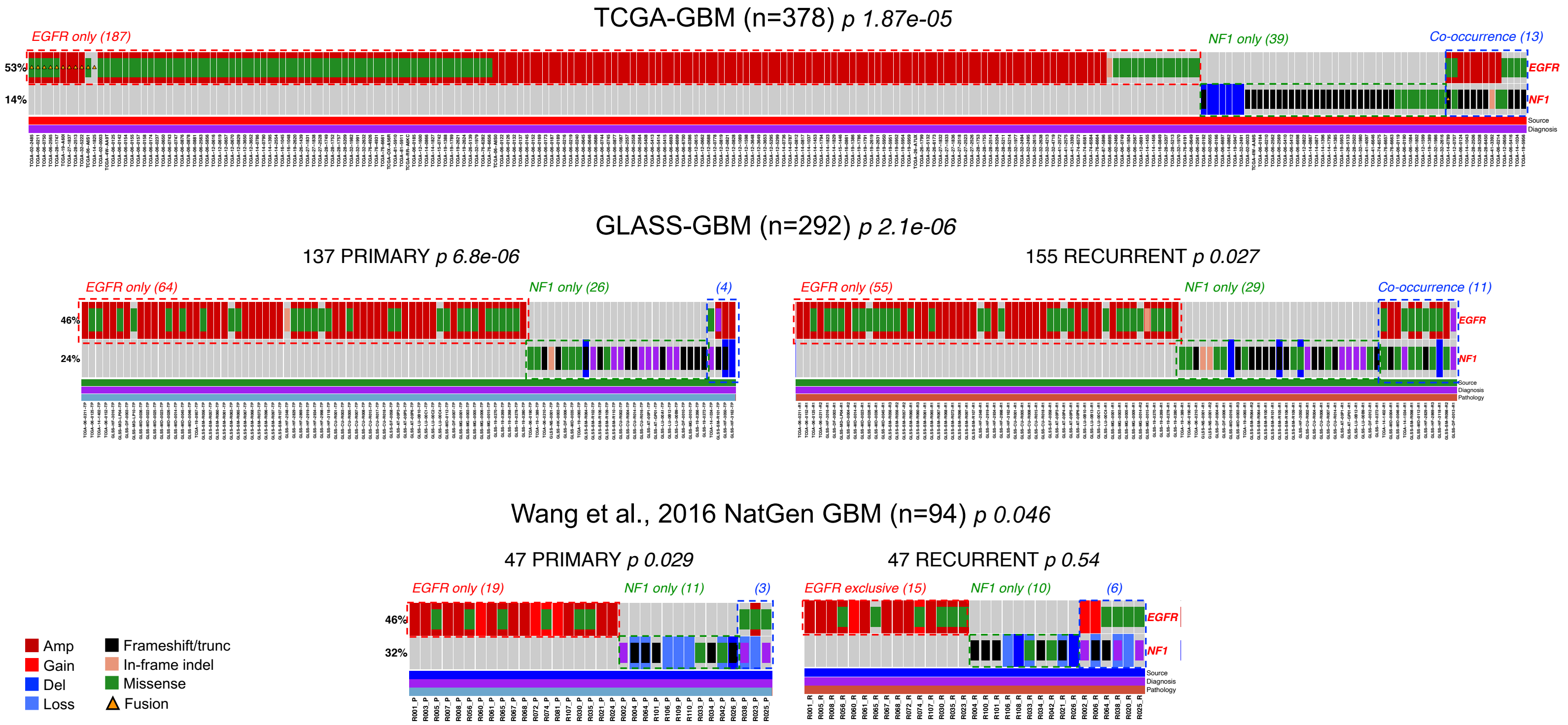

Supplementary Figure 4. The mutual exclusivity of EGFR and NF1 alteration observed in our dataset was validated using the TCGA-GBM cohort (n=378) [42] and two cohorts with data on primary and recurrent tumors: the GLASS consortium cohort (n=292) [47] and the Wang et al. cohort (n=94) [48]. Each column represents an individual sample, with mutation type coded by color according to the key (inset). P-values were calculated using two-sided Fisher's exact test.

Supplementary Figure 5.

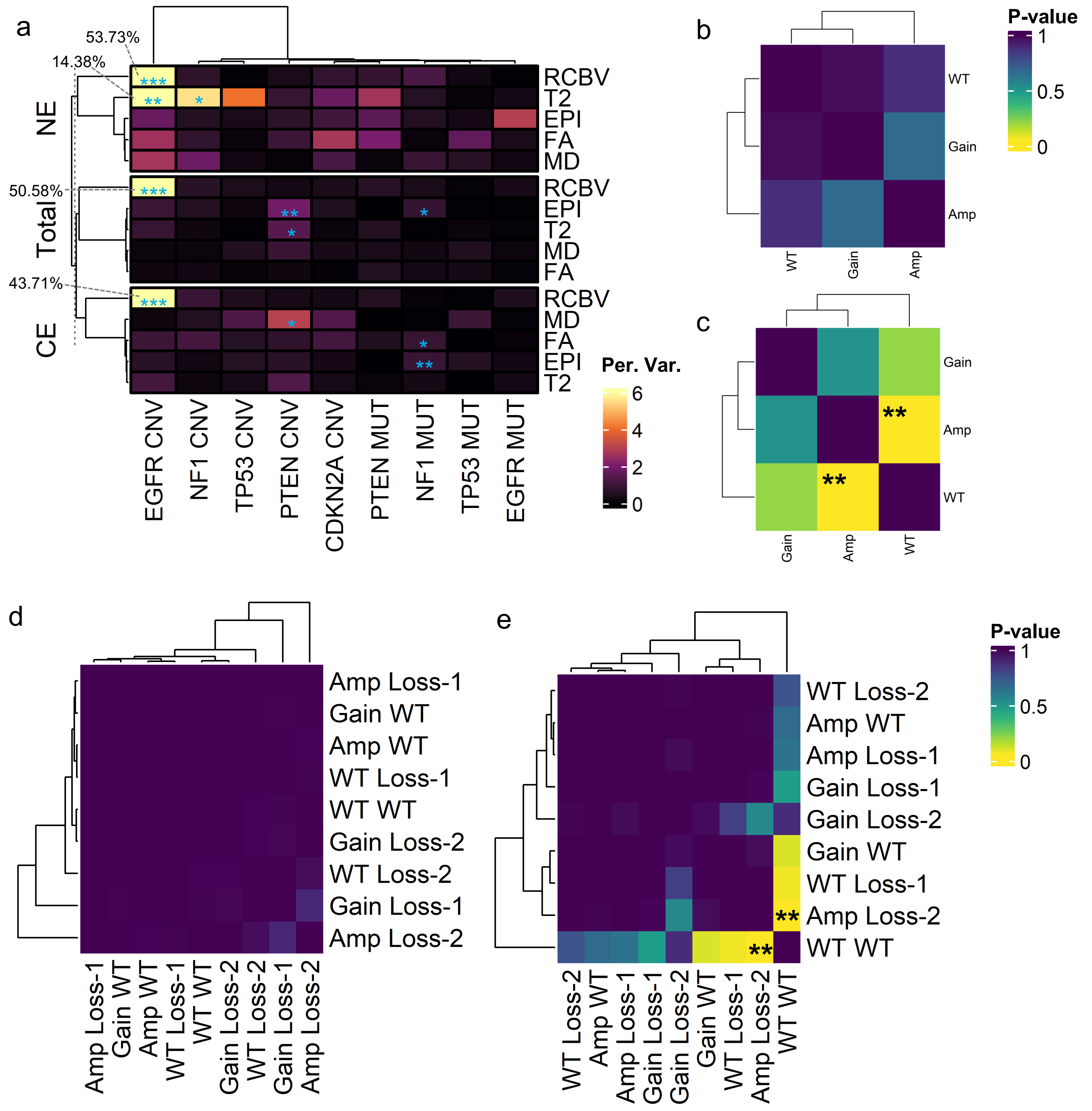

Supplementary Figure 5. a. The percent variance attributed to genotypic markers modeled as fixed effects (x-axis) in MEMs for each imaging variable (y-axis) separated by region. *p-value < 0.1 **p-value<0.05 ***p-value<0.001. Statistical test: ANOVA. Values above 6% are manually annotated with call outs connected to the relevant cell by dashed lines. b-c. MEM corrected pairwise p-values for EGFR CNV and its effect on MD in the CE (b) and NE (c) regions. d-e MEM corrected pairwise p-values for combinatorial EGFR CDKN2A CNV and its effect on MD in the CE (d) and NE (e) regions. b-e. Statistical test: two-sided t-test with Tukey correction. a-e. Source data are provided as a Source Data file.

Supplementary Figure 6.

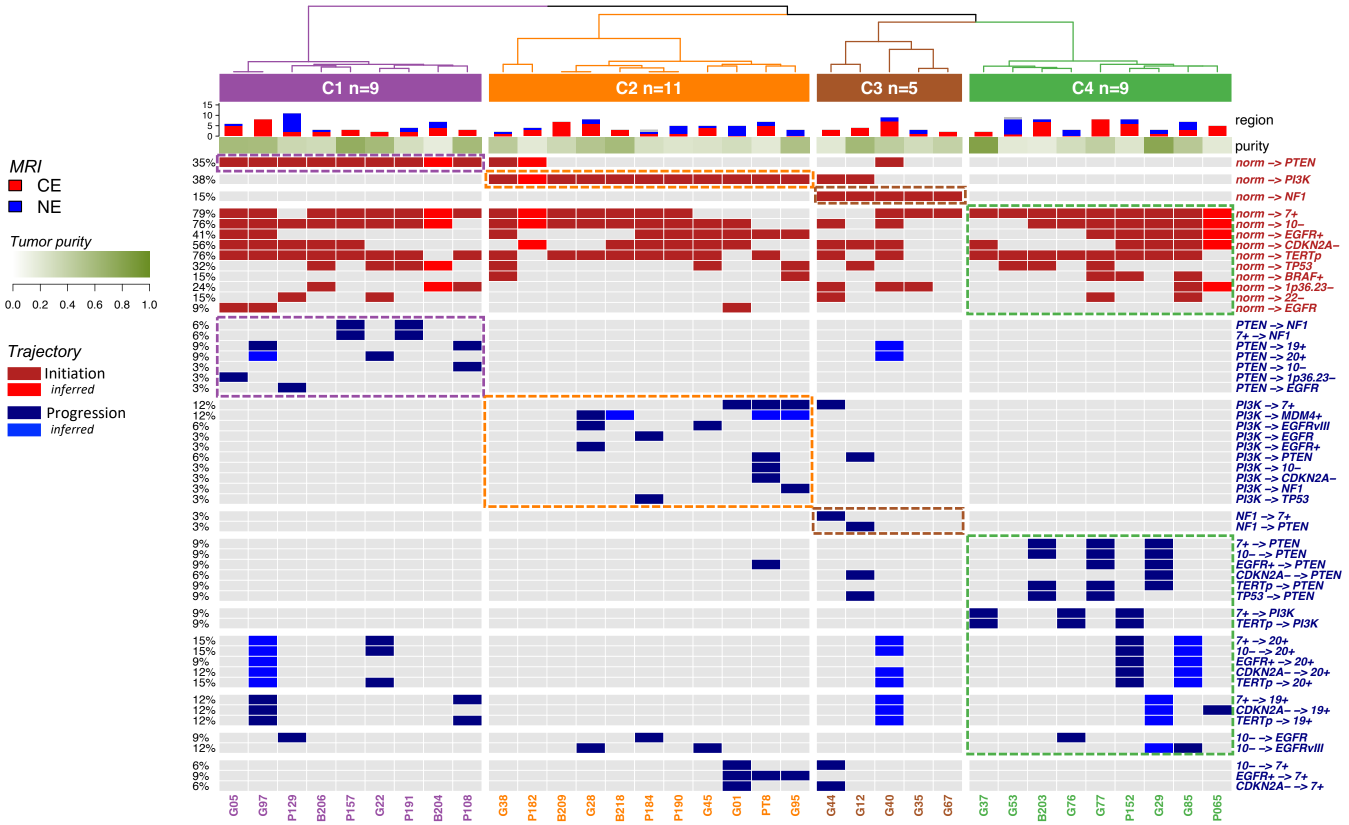

Supplementary Figure 6. Heatmap of molecular evolutionary trajectories across the IDH wild-type multiregional glioma cases (n=34). For each patient MRI contrast enhancing annotation and tumor purity are indicated (top barplot and heatmap track, respectively). Four different subgroups have been identified by supervised clustering of initiating trajectories.

Supplementary Figure 7.

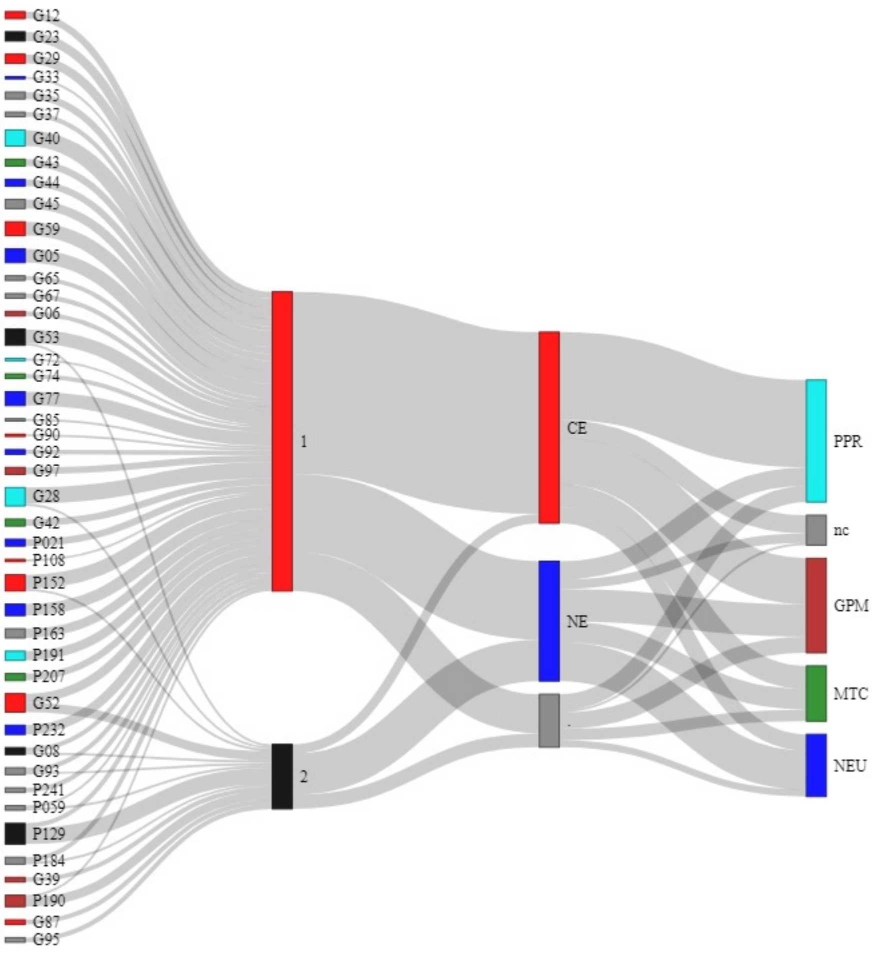

Supplementary Figure 7. Sankey plot showing patient sample classification into transcriptomic clusters, MRI region, MRI region, and pathway-based classification. Source data are provided as a Source Data file.

Supplementary Figure 8.

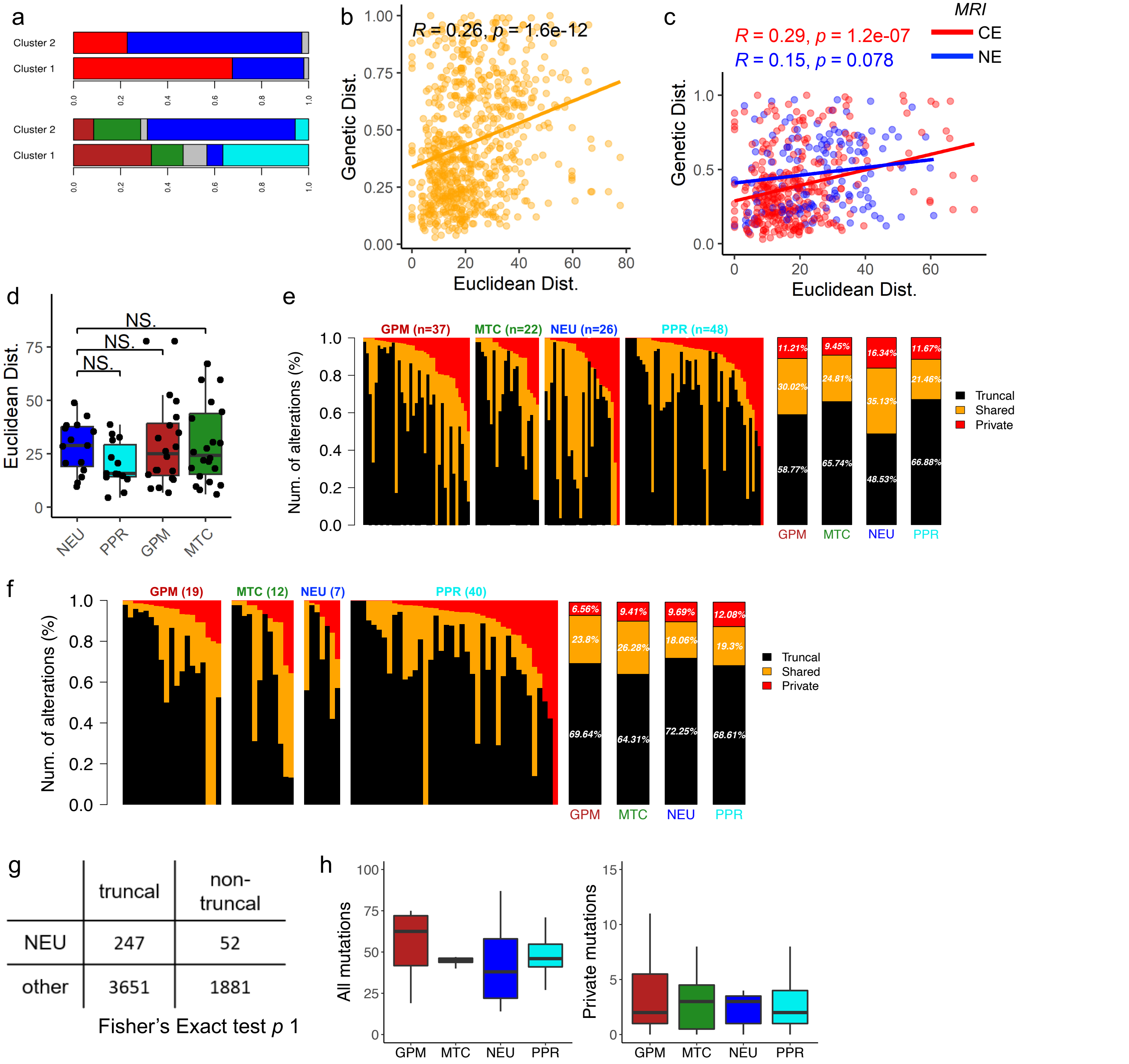

Supplementary Figure 8. a. Distribution of NE vs CE and pathway-based classification in the two detected transcriptomic clusters. b. Overall correlation between genetic and euclidean distance in all pairwise combinations of samples within a single patient (two-sided t-test, no correction). c. Correlation between the genetic and euclidean distance of pairs of samples within the same contrast region (two-sided t-test, no correction). d. Euclidean distance between CE samples and NE samples of a given pathway-based subtype (n=94 biopsy samples). Boxplots represent data minimum, 25th percentile, 50th percentile, 75th percentile, and maximum. Statistical test: two-sample t-test e. Private, shared, and truncal alterations in individual samples in all regions classified as each pathway-based subtype (from left to right: glycolytic/plurimetabolic, mitochondrial, neuronal, and proliferative/progenitor), with the average of private, shared, and truncal mutations for each pathway-based subtype displayed to the right. f. Private, shared, and truncal alterations in individual samples in the CE region classified as each Pathway-based classification subtype (from left to right: glycolytic/plurimetabolic, mitochondrial, neuronal, and proliferative/progenitor), with the average of private, shared, and truncal mutations for each pathway-based subtype displayed to the right. g. The proportion of truncal mutations vs. non-truncal (private and shared) mutations in samples of NEU subtype was not significantly different than the proportion of truncal mutations vs. non-truncal mutations in the other subtypes (one-tailed Fisher's exact test p = 1). h. Box and whisker plots show the absolute number of total (left) and private (right) mutations in each pathway-based subtype and the distribution of mutational burden across samples (n=78 biopsy samples). Boxplots represent data 25th percentile, 50th percentile and 75th percentile. The upper whisker extends from the upper hinge to the largest value no further than 1.5X IQR (inter-quartile range, or distance between the first and third quartiles) and the lower whisker extends from the lower hinge to the smallest value at most 1.5X IQR. b-f, h. Source data are provided as a Source Data file.

Supplementary Figure 9.

a

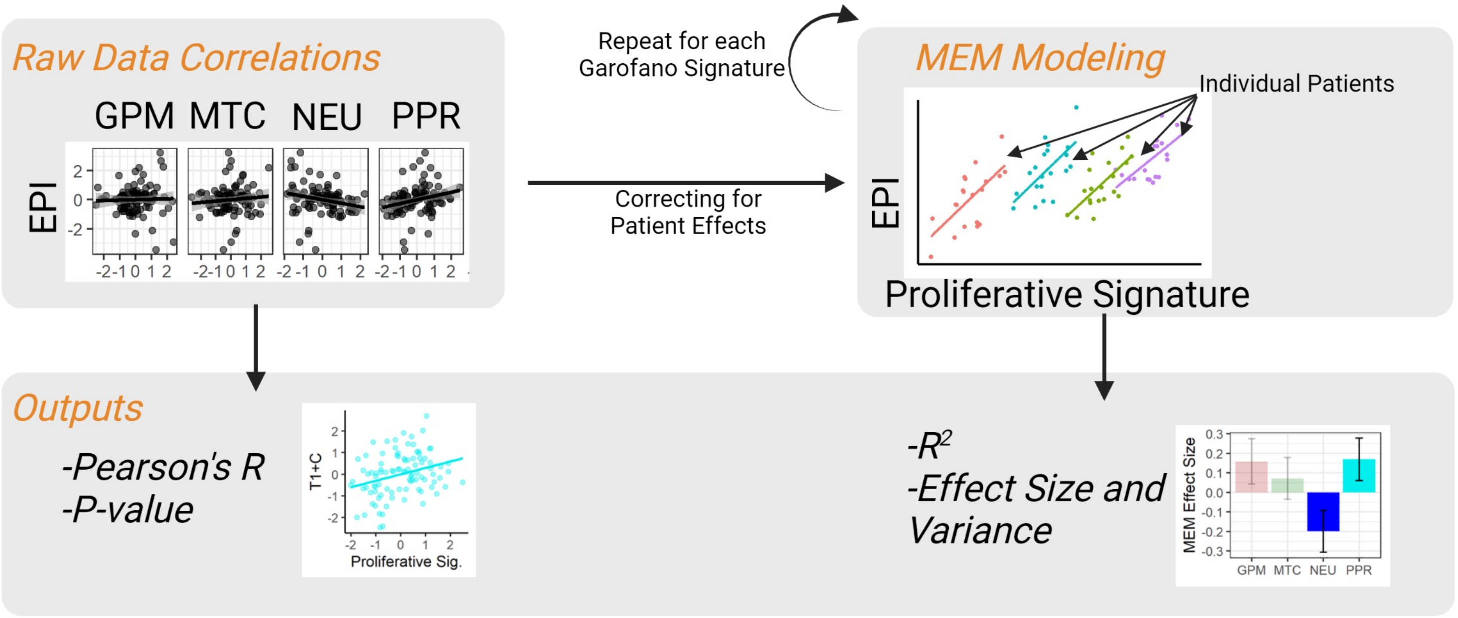

b

High Pearson's R	Low P-value	High R2	High MEM Est. Effect	Low MEM Effect SE	Interpretation
✓					Imaging metric and signature are correlated, but the spread is too random to be significant
✓	✓				Imaging metric and signature are significantly correlated, but the correlation is due to patient effects. The effect disappears when patient effects are corrected for.
✓	✓	✓			Imaging metric and signature are significantly correlated, and the MEM fits well. However, because the MEM effect is small this indicates that the MEM is fitting the data to a straight line meaning the values do not change in relation to one another and are stable at all values of the other
✓	✓	✓	✓		Imaging metric and signature are significantly correlated, the MEM fits well, and the variables affect each other. However, the MEM provides a high variance for the effect size indicating that there could be a large range of possible effect sizes.
✓	✓	✓	✓	✓	Imaging metric and signature are significantly correlated, the MEM fits well, and the variables affect each other reproducibly. The effect is likely significant and has an appreciable magnitude with of a low variance.

Supplementary Figure 9. a. Statistical workflow of accessing relationships between continuous pathway-based classification signature scores and MRI imaging features. Data shown is for example purposes only and is simulated for ease of visibility in the MEM Modeling section. b. Statistical interpretation guide for the outputs derived from S5a.

Supplementary Figure 10.

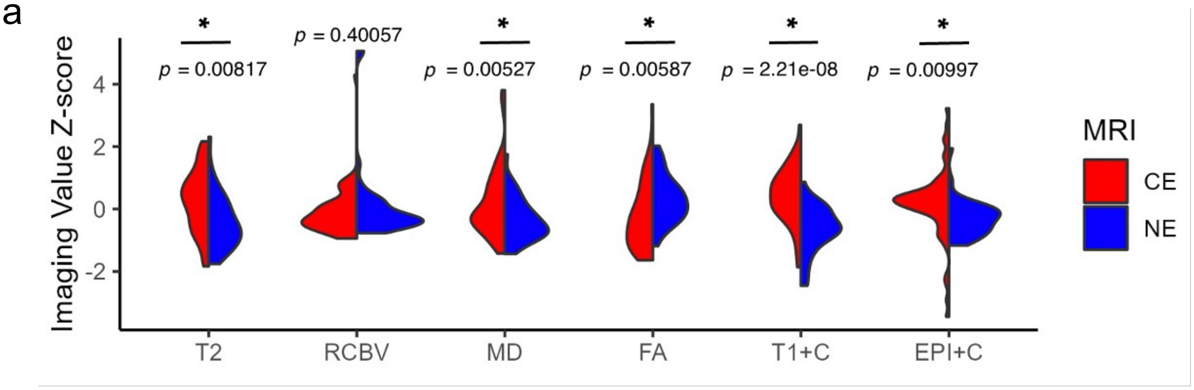

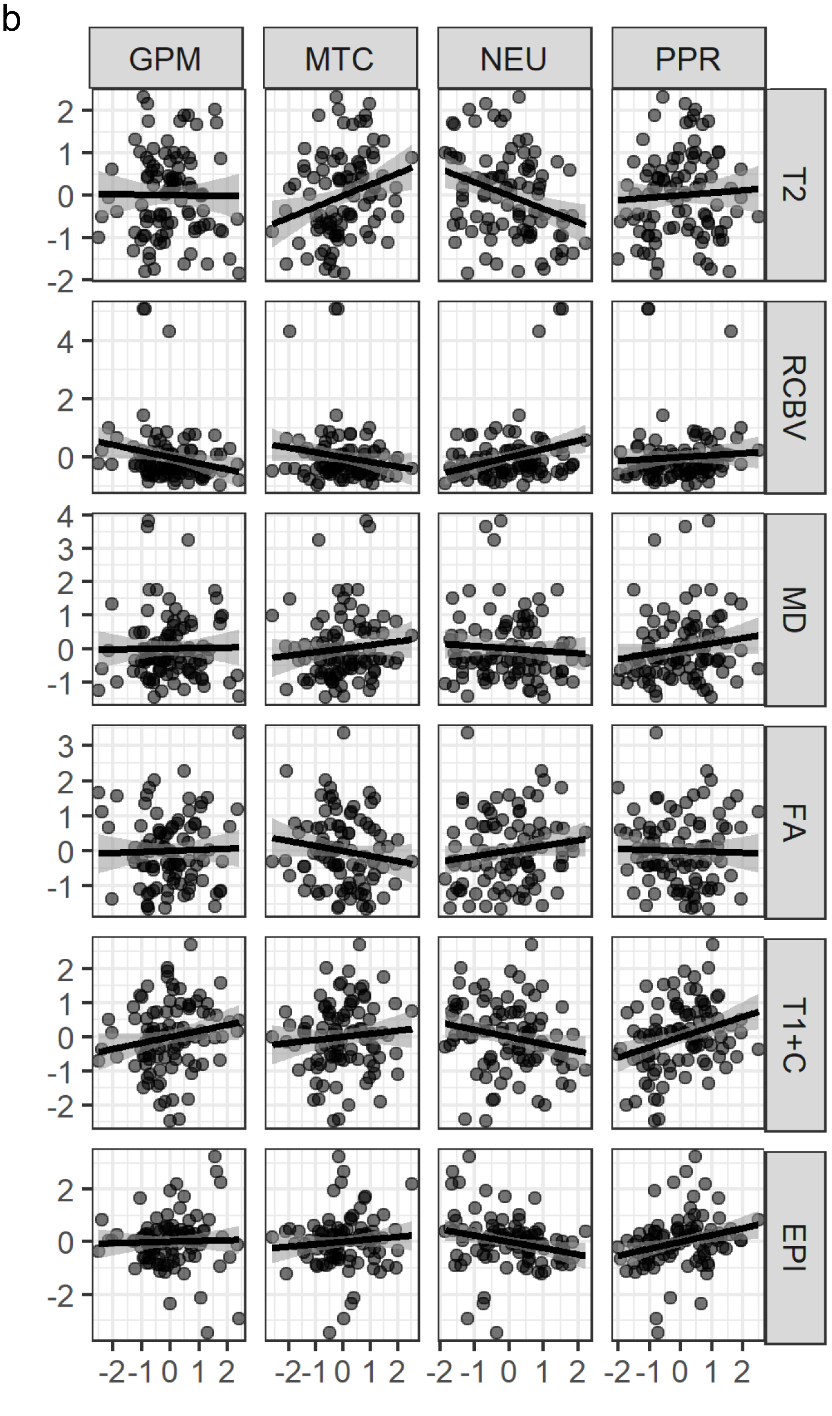

Supplementary Figure 10. a. Split violin plots showing the density distributions of the Z-scored imaging parameters. *=p-value<0.05. Statistical test: two-sided t-test, no correction. b. Correlation map of pathway-based signature (x-axis) and MRI features (y-axis) for all samples. All features are z-scored for improved visibility. Error bars represent 95% confidence intervals. GPM = glycolytic, MTC = MTC, NEU = NEU, PPR = proliferative. a-b. Source data are provided as a Source Data file.

Supplementary Figure 11.

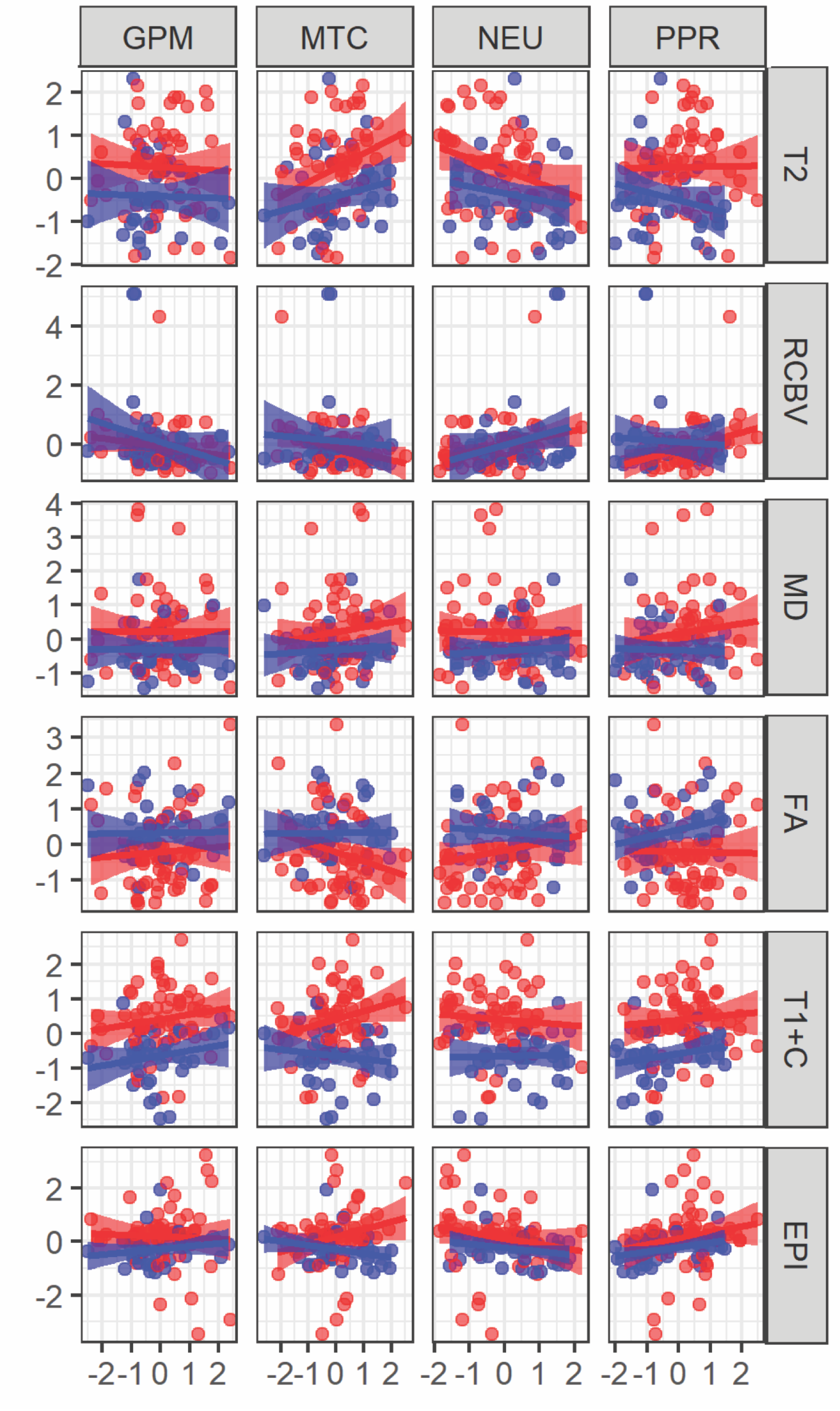

Supplementary Figure 11. Correlation map of pathway-based signature (x-axis) and MRI features (y-axis) stratified by contrast region with NE in blue and CE in red. All features are z-scored for improved visibility. Error bars represent 95% confidence intervals. GPM = glycolytic, MTC = MTC, NEU = NEU, PPR = proliferative. Source data are provided as a Source Data file.

Supplementary Figure 12.

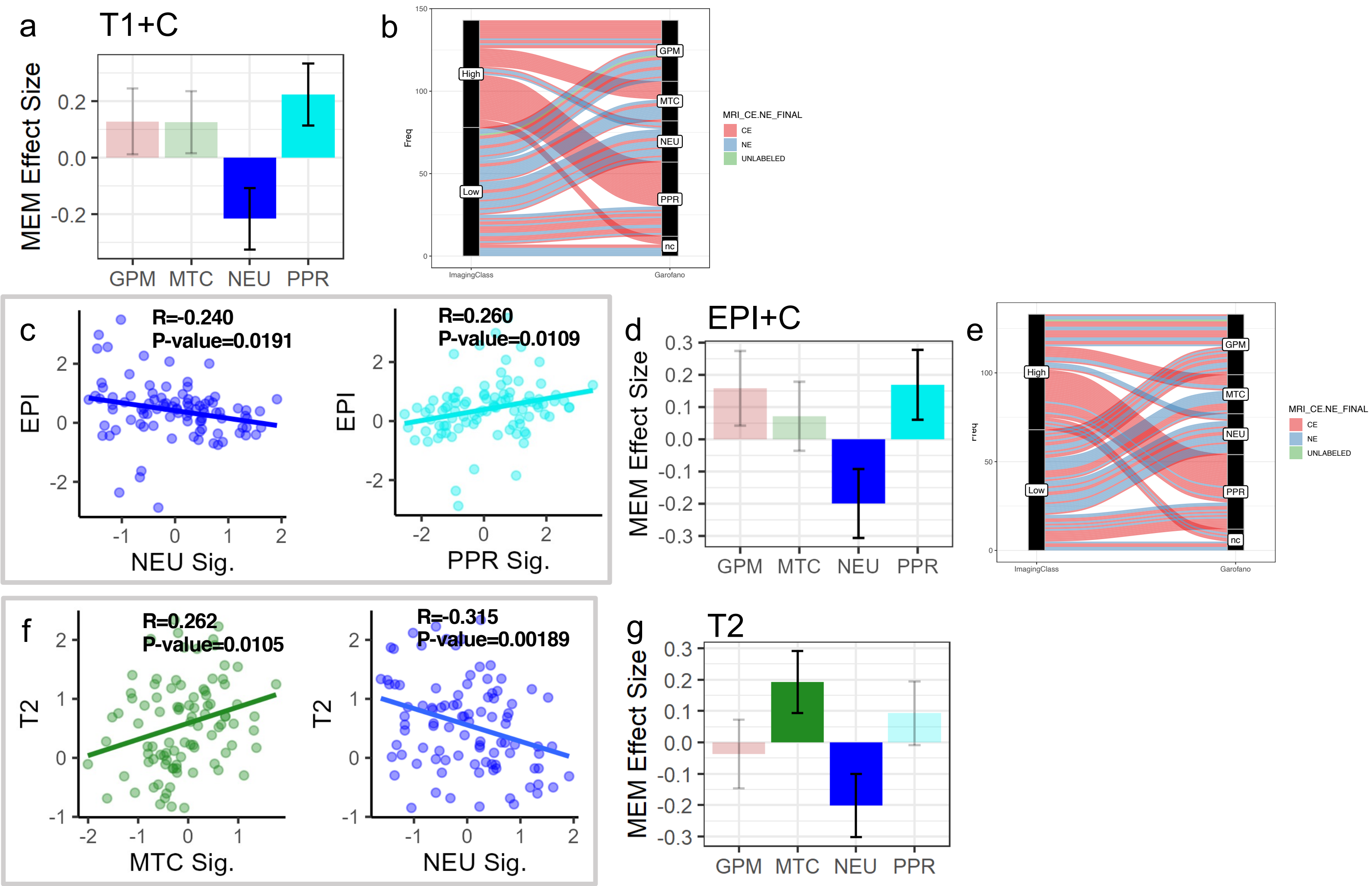

Supplementary Figure 12. a. MEM effect size (linear model slope) for a model describing the relationship between T1+C and each of the four pathway-based signatures. Faded bar color indicates non-statistically significant correlations. Plotted as MEM effect size +/- standard error. b. Sankey plot showing co-occurrence between samples grouped as T1+C high or low and pathway-based classification (as a categorical measure). c. Scatter plots showing significant correlations between EPI+C and NEU or PPR signatures across all samples (two-sided t-test, no correction). d. MEM effect size (linear model slope) for a model describing the relationship between EPI+C and each of the four pathway-based signatures. Faded bar color indicates non-statistically significant correlations. Plotted as MEM effect size +/- standard error. e. Sankey plot showing co-occurrence between samples grouped as EPI+C high or low and pathway-based classification (as a categorical measure). f. Scatter plots showing significant correlations between T2W and MTC or NEU signatures across all samples (two-sided t-test, no correction). g. MEM effect size (linear model slope) for a model describing the relationship between T2W and each of the four pathway-based signatures. Faded bar color indicates non-statistically significant correlations. Plotted as MEM effect size +/- standard error. (a-g, n=95). a, d, g. Data are presented as mean values +/- SD. a-g. Source data are provided as a Source Data file.

Supplementary Figure 13.

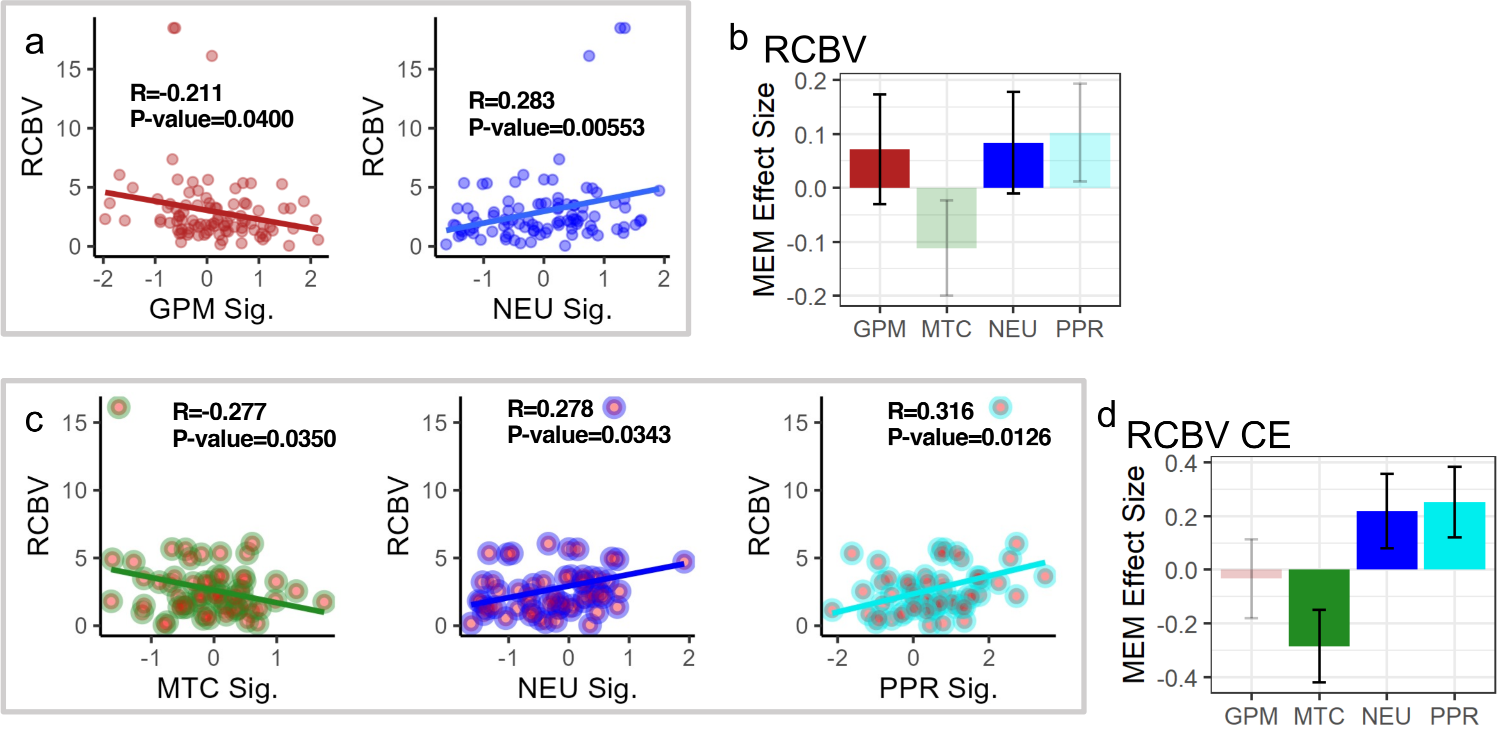

Supplementary Figure 13. a. Scatter plots showing significant correlations between RCBV and GPM or NEU signatures across all samples (two-sided t-test, no correction). b. MEM effect size (linear model slope) for a model describing the relationship between RCBV and each of the four pathway-based signatures. Faded bar color indicates non-statistically significant correlations (n=95). Plotted as MEM effect size +/- standard error. c. Scatter plots showing significant correlations between RCBV and MTC, NEU, or PPR signatures in CE samples only (indicated by red center on data points) (two-sided t-test, no correction). d. MEM effect size (linear model slope) for a model describing the relationship between RCBV and each of the four Pathway-based classification signatures in CE samples only. Faded bar color indicates non-statistically significant correlations (n=58). Plotted as MEM effect size +/- standard error. b, d. Data are presented as mean values +/- SD. a-d. Source data are provided as a Source Data file.

Supplementary Figure 14.

a

b

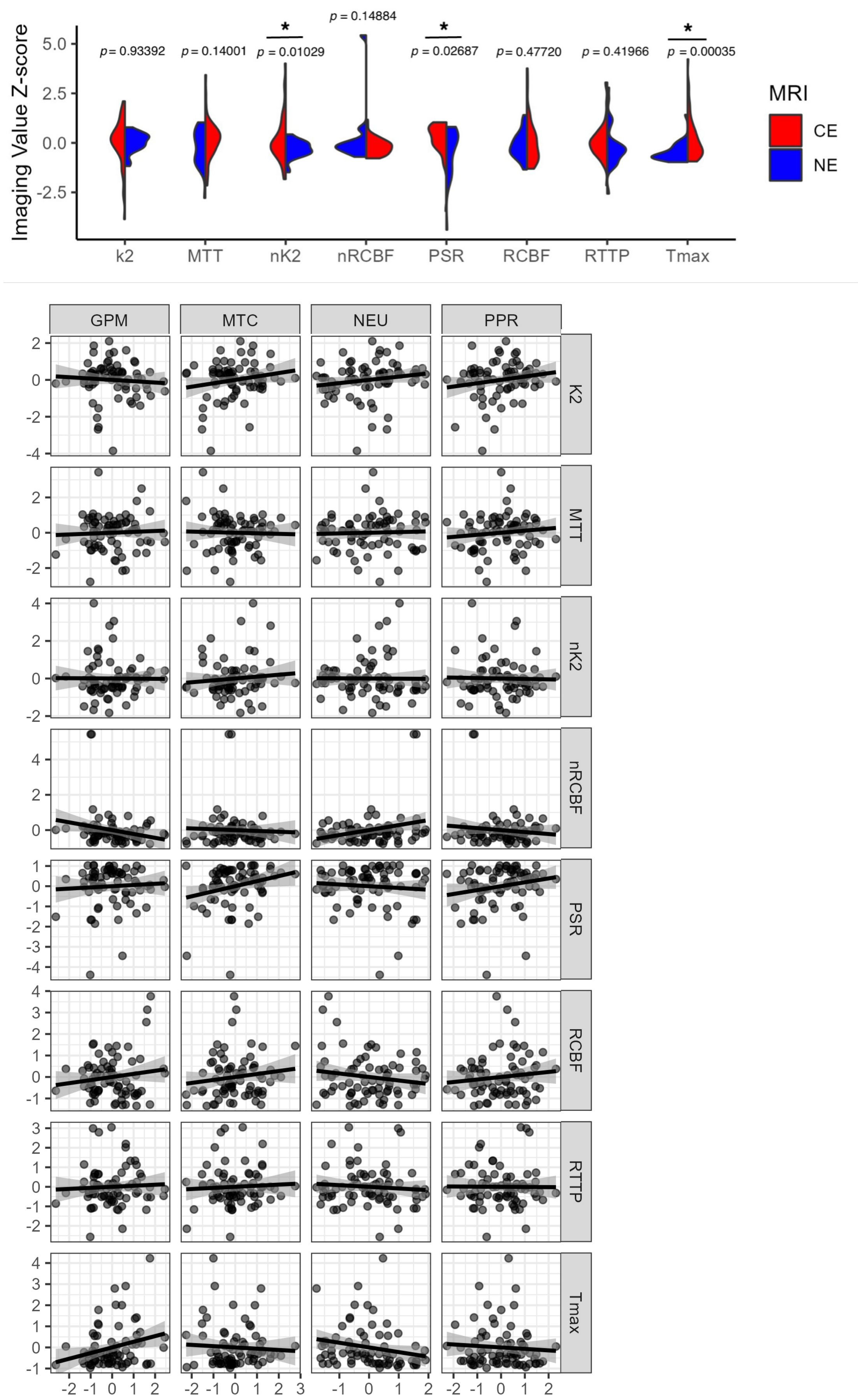

Supplementary Figure 14. a. Split violin plots showing the density distributions of the Z-scored imaging parameters. *=p-value<0.05. Statistical test: two-sided t-test, no correction. b. Correlation map of pathway-based signature (x-axis) and DSC-MRI features (y-axis) for all samples. All features are z-scored for improved visibility. Error bars represent 95% confidence intervals. GPM = glycolytic, MTC = MTC, NEU = NEU, PPR = proliferative. a-b. Source data are provided as a Source Data file.

Supplementary Figure 15.

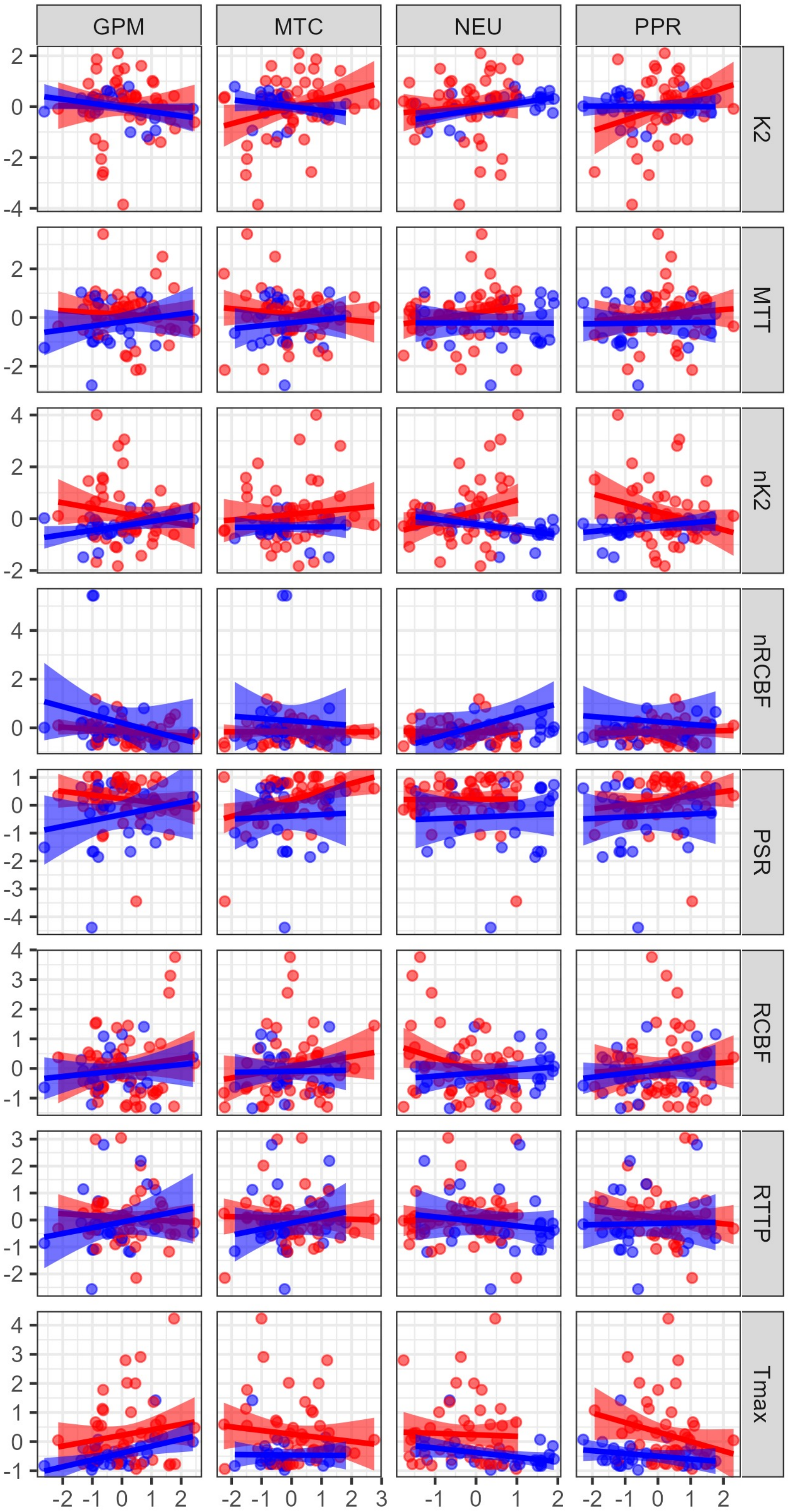

Supplementary Figure 15. Correlation map of pathway-based signature (x-axis) and DSC-MRI features (y-axis) stratified by contrast region with NE in blue and CE in red. All features are z-scored for improved visibility. Error bars represent 95% confidence intervals. GPM = glycolytic, MTC = MTC, NEU = NEU, PPR = proliferative.

Supplementary Figure 16.

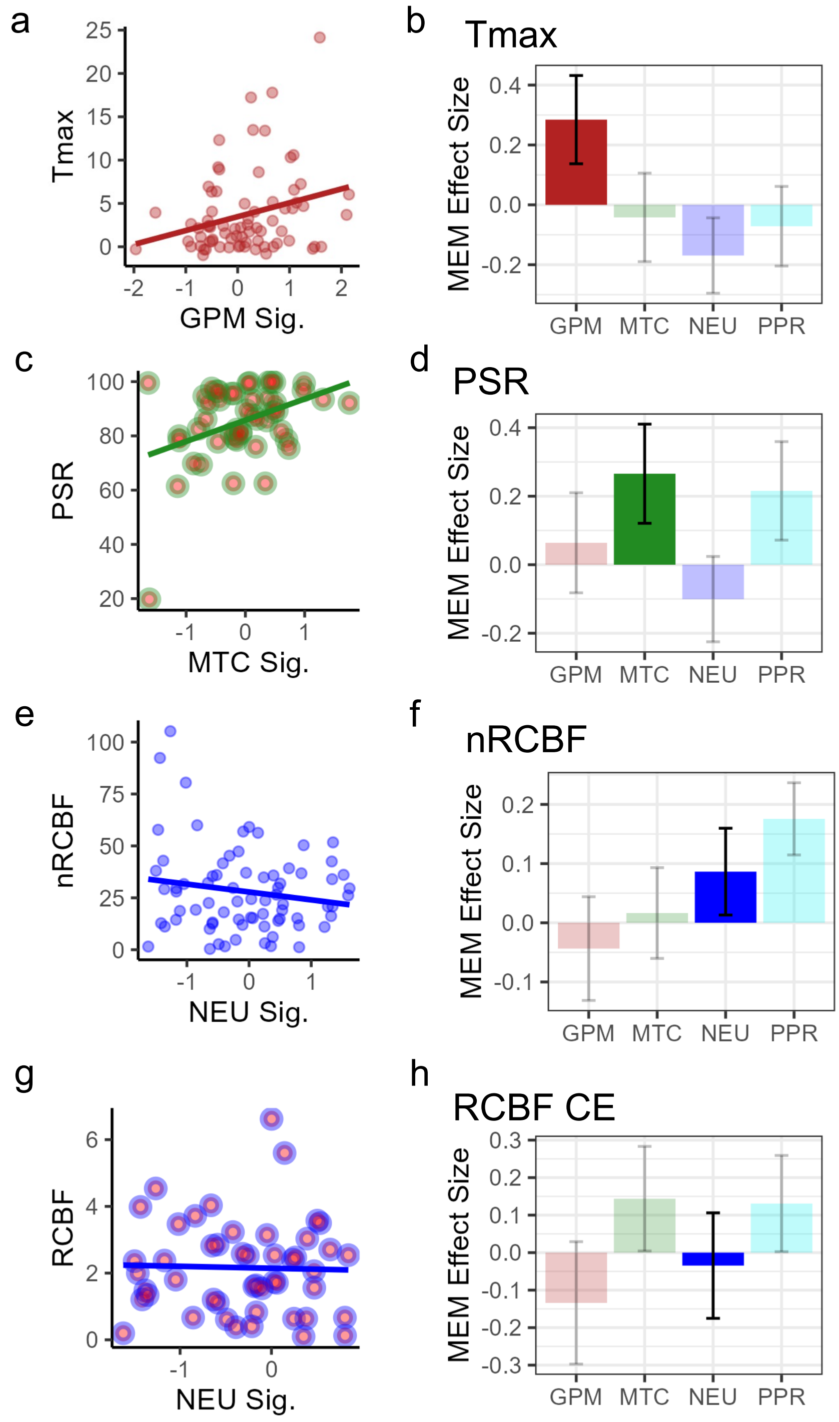

Supplementary Figure 16. a. Scatter plots showing significant correlations between Tmax and GPM signature across all samples (two-sided t-test, no correction). b. MEM effect size (linear model slope) for a model describing the relationship between Tmax and each of the four pathway-based signatures in all samples. Faded bar color indicates non-statistically significant correlations (n=74). Plotted as MEM effect size +/- standard error. c. Scatter plots showing significant correlations between PSR and MTC signature across all samples (two-sided t-test, no correction). d. MEM effect size (linear model slope) for a model describing the relationship between PSR and each of the four Pathway-based classification signatures in CE samples only. Faded bar color indicates nonstatistically significant correlations (n=48). Plotted as MEM effect size +/- standard error. e. Scatter plots showing significant correlations between nRCBF and NEU signature across all samples (two-sided t-test, no correction). f. MEM effect size (linear model slope) for a model describing the relationship between nRCBF and each of the four pathway-based signatures in all samples. Faded bar color indicates non-statistically significant correlations (n=74). Plotted as MEM effect size +/- standard error. g. Scatter plot showing a significant correlation between RCBF and the NEU signature in CE samples only (indicated by red center on data points) (two-sided t-test, no correction). h. MEM effect size (linear model slope) for a model describing the relationship between RCBF and each of the four pathway-based signatures in CE only samples. Faded bar color indicates non-statistically significant correlations (n=48). Plotted as MEM effect size +/- standard error. b, d, f, h. Data are presented as mean values +/- SD. a-h. Source data are provided as a Source Data file.

Supplementary Figure 17.

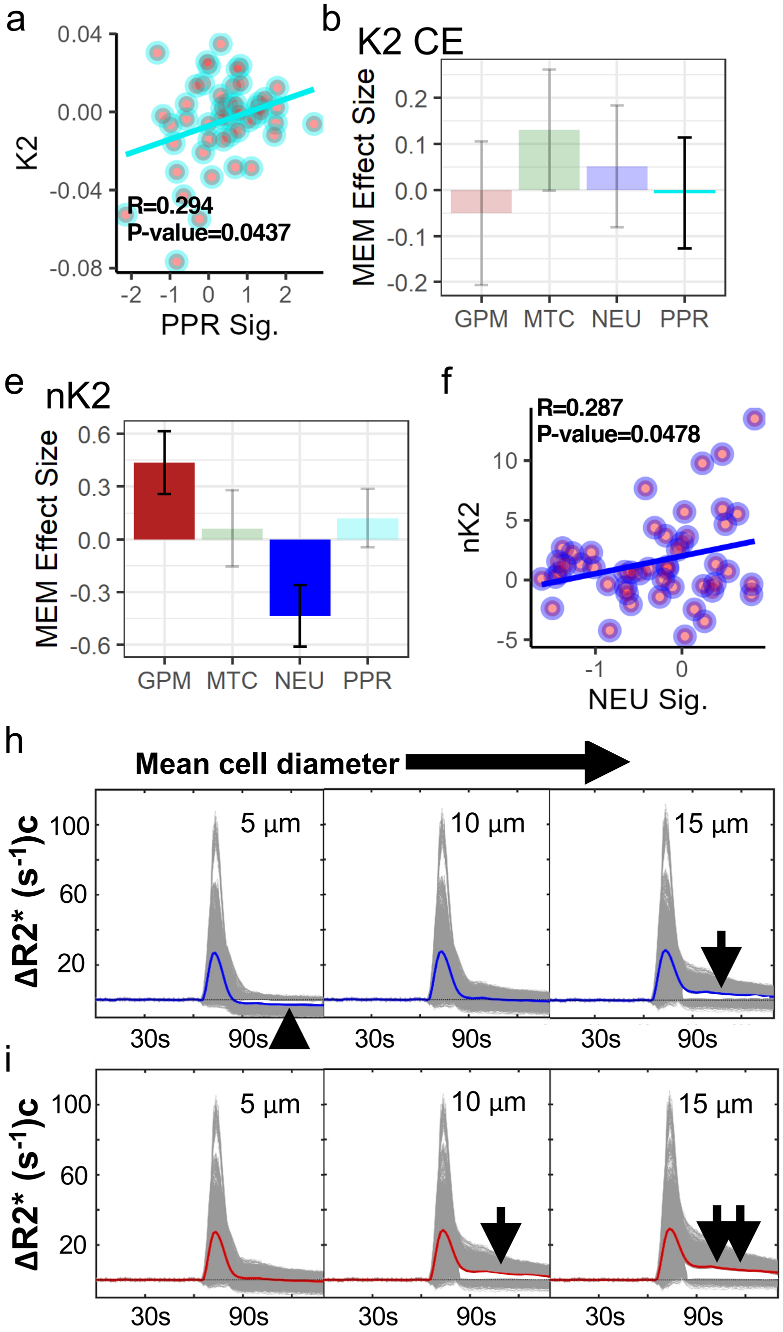

Supplementary Figure 17. a. Scatter plot showing a significant correlation between K2 and the PPR signature in CE samples only (indicated by red center on data points) (statistical test: two-sided t-test, no correction). b. MEM effect size (linear model slope) for a model describing the relationship between K2 and each of the four pathway-based signatures in CE only samples. Faded bar color indicates non-statistically significant correlations (n=48). Plotted as MEM effect size +/- standard error. c. Scatter plot showing a significant correlation between K2 and the NEU signature in NE samples only (indicated by red center on data points) (statistical test: two-sided t-test, no correction). d. MEM effect size (linear model slope) for a model describing the relationship between K2 and each of the four pathway-based signatures in NE only samples. Faded bar color indicates non-statistically significant correlations (n=26). Plotted as MEM effect size +/- standard error. e. MEM effect size (linear model slope) for a model describing the relationship between nK2 and each of the four pathway-based signatures in NE only samples. Faded bar color indicates non-statistically significant correlations (n=26). Plotted as MEM effect size +/- standard error. f. Scatter plot showing a significant correlation between nK2 and the NEU signature in CE samples only (indicated by red center on data points) (statistical test: two-sided t-test, no correction). g. MEM effect size (linear model slope) for a model describing the relationship between nK2 and each of the four pathway-based signatures in CE only samples. Faded bar color indicates non-statistically significant correlations (n=48). Plotted as MEM effect size +/- standard error. h. Relaxivity curves over time for a mean cell diameter of 5, 10, and 15 µm in the setting of homogeneous cell sizes. Arrows indicate where the tail of the signal curve extends below (5 µm) and above (15 µm) the normal tissue baseline. i. Relaxivity curves over time for a mean cell diameter of 5, 10, and 15 µm in the setting of heterogeneous cell sizes. Arrows indicate where the tail of the signal curve extends above (10 and 15 µm) the normal tissue baseline. b, d, e, g. Data are presented as mean values +/- SD. a-g. Source data are provided as a Source Data file.

Supplementary Figure 18.

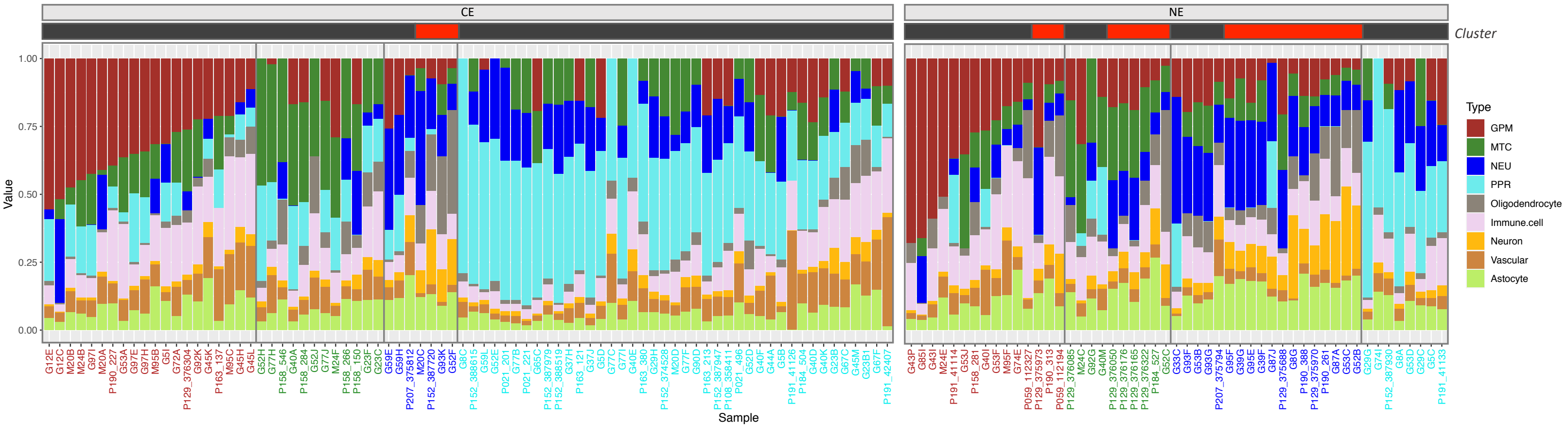

Supplementary Figure 18. Stacked bar plot showing the cell fraction composition of samples with CE (left) and NE (right) localization computed using CIBERSORTx. The top trace indicates the samples that fall into cluster 1 (black) and cluster 2 (red). Sample names are colored according to pathway-based classification.
