# Supplementary material for: Integrated molecular and multiparametric MRI mapping of high-grade glioma identifies regional biologic signatures
Source: Nat Commun. 2023 Sep 28;14:6066. doi: 10.1038/s41467-023-41559-1 (PMC10539500; doi:10.1038/s41467-023-41559-1)
Supplement: Supplementary file 3 — Reporting Summary [file 41467_2023_41559_MOESM3_ESM.pdf]

Corresponding author(s): Hu, D'Angelo, Ceccarelli, Iavarone, Tran

Last updated by author(s): 2023/08/14

Reporting Summary

Nature Portfolio wishes to improve the reproducibility of the work that we publish. This form provides structure for consistency and transparency in reporting. For further information on Nature Portfolio policies, see our Editorial Policies and the Editorial Policy Checklist.

Statistics

For all statistical analyses, confirm that the following items are present in the figure legend, table legend, main text, or Methods section.

n/a	Confirmed	
☐	☒	The exact sample size (*n*) for each experimental group/condition, given as a discrete number and unit of measurement
☐	☒	A statement on whether measurements were taken from distinct samples or whether the same sample was measured repeatedly
☐	☒	The statistical test(s) used AND whether they are one- or two-sided<br>*Only common tests should be described solely by name; describe more complex techniques in the Methods section.*
☐	☒	A description of all covariates tested
☐	☒	A description of any assumptions or corrections, such as tests of normality and adjustment for multiple comparisons
☐	☒	A full description of the statistical parameters including central tendency (e.g. means) or other basic estimates (e.g. regression coefficient) AND variation (e.g. standard deviation) or associated estimates of uncertainty (e.g. confidence intervals)
☐	☒	For null hypothesis testing, the test statistic (e.g. *F*, *t*, *r*) with confidence intervals, effect sizes, degrees of freedom and *P* value noted<br>*Give P values as exact values whenever suitable.*
☒	☐	For Bayesian analysis, information on the choice of priors and Markov chain Monte Carlo settings
☒	☐	For hierarchical and complex designs, identification of the appropriate level for tests and full reporting of outcomes
☐	☒	Estimates of effect sizes (e.g. Cohen's *d*, Pearson's *r*), indicating how they were calculated

Our web collection on statistics for biologists contains articles on many of the points above.

Software and code

Policy information about availability of computer code

Data collection	3T MRI (Sigma HDx; GE-Healthcare Waukesha Milwaukee; Ingenia, Philips Healthcare, Best, Netherlands; Magnetome Skyra; Siemens Healthcare, Erlangen Germany), Whole Exome Sequence (WES) was performed using SureSelect (Agilent) or Strexome V2 capture kits (TGen)
Data analysis	MRIConvert v2.1.0; FSL v5.0; SimpleITK v1.0.1; Python v3.6.2; IB Neuro, IB Delta Suite, IB Rad Tech (v21.12); Freebayes (arXiv:1207.3907) MuTect2, TNhaplotyper, TNscope, TNsnv, VarScan2, AnnoVar, PureCN, GISTIC2, STARfusion, AGFusion, CTAT-Splicing, PhyC, REVOLVER, UCSC genome assembly GRCh37, STAR (v. 2.7.0b). Batch normalization - EDaseq R package (v. 2.22.0). Batch adjustment - sva R package. Differential expression - EdgeR R package (v. 3.30.3). Gene set enrichment analysis - ClusterProfiler R package (v. 3.3.6). Deconvolution - scTHI R package and Molecular Signature Database (MSigDB), CIBERSORTx.

For manuscripts utilizing custom algorithms or software that are central to the research but not yet described in published literature, software must be made available to editors and reviewers. We strongly encourage code deposition in a community repository (e.g. GitHub). See the Nature Portfolio guidelines for submitting code & software for further information.

Data

Policy information about availability of data

All manuscripts must include a data availability statement. This statement should provide the following information, where applicable:

- Accession codes, unique identifiers, or web links for publicly available datasets
- A description of any restrictions on data availability
- For clinical datasets or third party data, please ensure that the statement adheres to our policy

All datasets analyzed in the current study, including whole exome sequencing and RNA sequencing, are publicly accessible, with the Synapse (https://www.synapse.org/#!Synapse:syn52256644). The publicly available bulk RNA-seq data for TCGA-GBM were obtained from the UCSC Xena browser [https://gdc-hub.s3.us-east-1.amazonaws.com/download/TCGA-GBM.htseq_counts.tsv.gz]. The remaining data are available within the Article, Supplementary Information or Source Data file. Source data are provided with this paper. The input data for the imaging analysis can be found at https://github.com/HuLiLab/Multi-Regional-GBM-Imaging-and-Genetics

Research involving human participants, their data, or biological material

Policy information about studies with human participants or human data. See also policy information about sex, gender (identity/presentation), and sexual orientation and race, ethnicity and racism.

Reporting on sex and gender	This study focused on the multi-regional molecular/genomic heterogeneity across a cohort of 68 glioma patients, with particular attention to the subsets of contrast-enhancing (CE) and non-enhancing (NE) biopsy samples. Tissue samples were further subdivided based on IDH mutation status (e.g., wildtype vs. mutant). The resultant reduction in sample sizes within these subgroups prevented us from further separating biopsy samples based on patient sex, given the diminished power of correlative analyses within this unique dataset. Future studies with expanded cohorts should provide opportunities to separate correlative analyses based on sex.
Reporting on race, ethnicity, or other socially relevant groupings	This study focused on the multi-regional molecular/genomic heterogeneity across a cohort of 68 glioma patients, with particular attention to the subsets of contrast-enhancing (CE) and non-enhancing (NE) biopsy samples. Tissue samples were further subdivided based on IDH mutation status (e.g., wildtype vs. mutant). The resultant reduction in sample sizes within these subgroups prevented us from further separating biopsy samples based on patient ethnicity given the diminished power of correlative analyses within this unique dataset. Future studies with expanded cohorts should provide opportunities to separate correlative analyses based on ethnicity.
Population characteristics	We limited this patient cohort to those adult patients (18 years or older) with the histological diagnosis of glioma, including both IDH-wildtype and IDH-mutant populations.
Recruitment	Patient recruitment was performed at two neighboring academic institutions (BNI, MCA), which involved identification of adult patients undergoing pre-operative MRI for surgical biopsy/resection of suspected glioma. Given the rarity of this disease, we did not limit recruitment based on demographics (e.g., sex, ethnicity).
Ethics oversight	Institutional Review Boards at Barrow Neurological Institute (BNI) and Mayo Clinic, Arizona (MCA)

Note that full information on the approval of the study protocol must also be provided in the manuscript.

Field-specific reporting

Please select the one below that is the best fit for your research. If you are not sure, read the appropriate sections before making your selection.

☒ Life sciences ☐ Behavioural & social sciences ☐ Ecological, evolutionary & environmental sciences

For a reference copy of the document with all sections, see nature.com/documents/nr-reporting-summary-flat.pdf

Life sciences study design

All studies must disclose on these points even when the disclosure is negative.

Sample size	Sample size for each experiment was determined by using all patients that had the needed data available. For example, to be considered for genetic and imaging modeling patients had to have both imaging features and genetics data collected.
Data exclusions	Image Quality Assessment: Each biopsy location was also assessed for potential image artifacts that could obscure signal intensity values, including artifacts at the interface of bone or air (e.g., floor of the anterior skull base, middle cranial fossa superior to the mastoid air cells), as well as metallic artifacts from prior surgical instrumentation (e.g., craniectomy plate/screws). We also denoted biopsy locations which were recorded centrally within locations that were expected to yield no tissue (e.g., resection cavity and/or central necrosis). We excluded those biopsies meeting these criteria from correlative analysis with all imaging techniques. We also excluded biopsy samples in close proximity to large surface vessels (e.g., middle cerebral artery branches), from correlative analysis with DSC-MRI based image features (e.g., rCBV maps), which are susceptible to artifacts from these large vessels.
Replication	Replication of experiments does not apply to our study. This is a one of a kind cohort due to the depth of information available on a per

Replication	patient basis. We ensure the validity of our findings using stringent statistical thresholds and validate against literature where possible.
Randomization	This study entailed descriptive and correlative studies of multi-regional genomic, transcriptomic, and imaging features in a cohort of glioma patients. The correlative studies included subgroup analyses based on several established clinical factors, including IDH status, as well as distinction between biopsy samples from the contrast-enhancing (CE) core and the invasive non-enhancing (NE) regions, which were determined based on standard clinical criteria for MRI-guided surgical biopsy/resection.
Blinding	Surgical tissue sampling was performed based on standard clinical protocol, from contrast-enhancing (CE) and non-enhancing (NE) regions on MRI-guided neuronavigational platforms. Sampling was performed blinded to advanced MRI features. Genomic, transcriptomic, and pathway-based analyses were performed only in the context of clinical information and the NE/CE annotation for each biopsy, but otherwise blinded to MRI features.

Reporting for specific materials, systems and methods

We require information from authors about some types of materials, experimental systems and methods used in many studies. Here, indicate whether each material, system or method listed is relevant to your study. If you are not sure if a list item applies to your research, read the appropriate section before selecting a response.

Materials & experimental systems

n/a	Involved in the study
☒	☐ Antibodies
☒	☐ Eukaryotic cell lines
☒	☐ Palaeontology and archaeology
☒	☐ Animals and other organisms
☒	☐ Clinical data
☒	☐ Dual use research of concern
☒	☐ Plants

Methods

n/a	Involved in the study
☒	☐ ChIP-seq
☒	☐ Flow cytometry
☐	☒ MRI-based neuroimaging

Magnetic resonance imaging

Experimental design

Design type	no functional MRI was performed
Design specifications	no functional MRI was performed
Behavioral performance measures	no functional MRI was performed

Acquisition

Imaging type(s)	Conventional sequences: T1W, T2W, T1W post-contrast, DSC perfusion MRI, Diffusion Tensor Imaging (DTI); EPI+C consisted of the T2*W image from the DSC-MRI sequence approximately 6 minutes following administration of single dose of gadolinium based contrast agent
Field strength	all scans performed at 3T field strength
Sequence & imaging parameters	T1W images were acquired using spoiled gradient recalled-echo inversion-recovery prepped (SPGR-IR prepped) (TI/TR/TE=300/6.8/2.8 ms; matrix=320×224; FOV=26 cm; thickness=2 mm). T2W images were acquired using fast-spin-echo (FSE) (TR/TE=5133/78 ms; matrix=320×192; FOV=26 cm; thickness=2 mm). DTI imaging was performed using Spin-Echo Echo-planar imaging (EPI) (TR/TE 10,000/85.2 ms, matrix 256×256; FOV 30 cm, 3 mm slice, 30 directions, ASSET, B =0,1000). For all patients (BNI and MCA), we administered a preload dose (PLD) of GBCA (0.1 mmol/kg) to minimize T1W leakage effects prior to DSC-MRI acquisition for measurement of relative cerebral blood volume (rCBV). After PLD, we employed a Gradient-echo (GE) EPI (TR/TE/flip angle=1500 ms/20 ms/60°, matrix 128×128, thickness 5 mm) DSC MRI acquisition for 3 min. At 45 s after the start of the DSC-MRI sequence, we administered a second GBCA bolus injection (0.05 mmol/kg at BNI; 0.1 mmol/kg at MCA), which was used to calculate rCBV maps for all patients. For patients recruited at MCA, we also employed a DSC-MRI acquisition during the contrast bolus administration of the PLD. Prior to PLD injection, we employed a Gradient-echo (GE) EPI (TR/TE/flip angle=1500 ms/20-30 ms/30°, matrix 128×128, thickness 5 mm) DSC MRI acquisition for 3 min. At 45 s after the start of the DSC sequence, we administered the 0.1 mmol/kg i.v. bolus injection of GBCA, which was used to calculate non-rCBV related DSC-MRI metrics (e.g., nK2, PSR, MTT).
Area of acquisition	whole brain or near-whole brain coverage

Diffusion MRI ☒ Used ☐ Not used

Parameters *Specify # of directions, b-values, whether single shell or multi-shell, and if cardiac gating was used.*

Preprocessing

Preprocessing software	We coregistered all datasets to the relatively high-quality DTI B0 anatomical image volume using tools from ITK and IB Delta Suite (Imaging Biometrics, LLC). Ultimately, the coregistered data exhibited in plane voxel resolution of~1.17 mm (256×256 matrix) and slice thickness of 3 mm.
Normalization	We performed N4 normalization of all non-quantitative maps, including T1+C, T2W, and EPI+C images. The python library from SimpleITK (www.simpleitk.com) was employed for all steps of normalization. Image denoising was first performed using sitk. CurvatureFlow, followed by N4 bias correction using sitk. N4BiasFieldCorrection. Intensity normalization was subsequently performed using a brain mask generated with sitk. MaskImageFilter for each image. Normalization was performed based on median signal intensities from a whole brain mask generated from each imaging sequence for each individual patient.
Normalization template	no standardized template was used
Noise and artifact removal	Image Quality Assessment: Each biopsy location was also assessed for potential image artifacts that could obscure signal intensity values, including artifacts at the interface of bone or air (e.g., floor of the anterior skull base, middle cranial fossa superior to the mastoid air cells), as well as metallic artifacts from prior surgical instrumentation (e.g., craniectomy plate/ screws). We also denoted biopsy locations which were recorded centrally within locations that were expected to yield no tissue (e.g., resection cavity and/or central necrosis). We excluded those biopsies meeting these criteria from correlative analysis with all imaging techniques. We also excluded biopsy samples in close proximity to large surface vessels (e.g., middle cerebral artery branches), from correlative analysis with DSC-MRI based image features (e.g., rCBV maps), which are susceptible to artifacts from these large vessels.
Volume censoring	no volume censoring was employed

Statistical modeling & inference

Model type and settings	Mass univariate mixed effect models with random effects to control for inter patient variability
Effect(s) tested	No tasks or stimuli tested. ANOVA design

Specify type of analysis: ☐ Whole brain ☒ ROI-based ☐ Both

Anatomical location(s)	ROI locations were determined based on the stereotactic locations of each corresponding biopsy sample, as described in supplemental figure 2 and methods in the manuscript
Statistic type for inference (See Eklund et al. 2016)	mean values obtained from each ROI.
Correction	only corrections using mixed effect models for inter patient variability as described above

Models & analysis

n/a	Involved in the study
☒	☐ Functional and/or effective connectivity
☒	☐ Graph analysis
☒	☐ Multivariate modeling or predictive analysis
