# Supplementary material for: Integrated molecular and multiparametric MRI mapping of high-grade glioma identifies regional biologic signatures
Source: Nat Commun. 2023 Sep 28;14:6066. doi: 10.1038/s41467-023-41559-1 (PMC10539500; doi:10.1038/s41467-023-41559-1)
Supplement: Supplementary file 4 — Description of additional supplementary files [file 41467_2023_41559_MOESM4_ESM.pdf]

Description of Additional Supplementary Files Document

Supplementary Data 1 – GENOMICS -- Multiregional cohort information, Case Assay, Somatic Mutations, Gene Level Copy Number

Supplementary Data 2 - Statistical values for additional MEM models.

Supplementary Data 3 – TRANSCRIPTOMICS -- Combat counts, pathway-based classifications, transcriptomic classifications

Supplementary Data 4 – GENE ONTOLOGY – differentially expressed genes, tumor microenvironment signatures

Supplementary Data 5 – Correlation between pathway-based classifications and conventional MRI features

Supplementary Data 6 – Correlation between pathway-based classifications and advanced imaging features

Supplementary Data 7 - Sample Sizes for Correlative Analyses
