# Supplementary material for: Integrated molecular and multiparametric MRI mapping of high-grade glioma identifies regional biologic signatures
Source: Nat Commun. 2023 Sep 28;14:6066. doi: 10.1038/s41467-023-41559-1 (PMC10539500; doi:10.1038/s41467-023-41559-1)
Supplement: Supplementary file 2 — Peer Review File [file 41467_2023_41559_MOESM2_ESM.pdf]

nature portfolio

Peer Review File

Integrated molecular and multiparametric MRI mapping of high-grade glioma identifies regional biologic signatures

Open Access This file is licensed under a Creative Commons Attribution 4.0 International License, which permits use, sharing, adaptation, distribution and reproduction in any medium or format, as long as you give appropriate credit to the original author(s) and the source, provide a link to the Creative Commons license, and indicate if changes were made. In the cases where the authors are anonymous, such as is the case for the reports of anonymous peer reviewers, author attribution should be to 'Anonymous Referee' followed by a clear attribution to the source work. The images or other third party material in this file are included in the article's Creative Commons license, unless indicated otherwise in a credit line to the material. If material is not included in the article's Creative Commons license and your intended use is not permitted by statutory regulation or exceeds the permitted use, you will need to obtain permission directly from the copyright holder. To view a copy of this license, visit http://creativecommons.org/licenses/by/4.0/.

REVIEWER COMMENTS

Reviewer #1 (Remarks to the Author): expertise in GBM clinical pathology

Review of NCOMMS-23-06819-T

In their manuscript, “Integrated molecular and multiparametric MRI mapping of high-grade glioma identifies regional biologic signatures,” Hu et al. analyze spatially correlated tumor molecular features and radiologic imaging data from a relatively large cohort to characterize infiltrative areas of tumor that often are the basis of tumor recurrence, compare these areas to the center of the tumor, and to add some understanding to glioma molecular evolution. The study is well-done, particularly given the stereotactic biopsy technique and extensive molecular studies, and adds valuable data to the field.

A few questions arise:

Was the designation "high-grade" determined by imaging (contrast enhancement), or based on histologic verification? What were the criteria for calling a glioma high-grade?

In figure 2, only four of the eleven tumors demonstrate both CE and NE samples. Does this have an impact on the interpretation of the comparison of molecular findings in CE and NE areas? Can findings in entirely NE tumors be extrapolated to NE areas of tumors with CE? Do the tumors without sampling of CE have unsampled areas of CE? Could tumors that are entirely non-CE have different properties (more heterogeneity) than those that have CE? In other words, are these different kinds of tumors, rather than different kinds of areas in a single type of tumor?

For recurrent tumors, for example in figures 2 and 3, is the CE due to inherent properties of the tumor or due to treatment/prior surgery, or both? The interpretation suggests that CE is strictly an indication of tumor properties, and molecular features seem to be interpreted in this light. Please clarify.

The NE oligodendroglioma in figure 2, second from right, appears to be graded "IV." Oligodendrogliomas are graded 2 or 3; please clarify the meaning of a grade IV oligodendroglioma, particularly in the absence of CE.

Could there be shift or other reasons for imperfect localization occurring that prevents perfect correlation of the biopsy based on the pre-op MRI? As the authors describe, CE is believed to result from breakdown of the blood-brain-barrier. For cases where the grade is II (no histologic evidence of BBB

breakdown) but the imaging is CE, there is a lack of correlation of histology and radiology. Similarly, many cases in the IDH-wt cohort show grade IV histology in NE areas. That leads to the question of with how much confidence claims can be about the "periphery" of the tumors.

In Figure 4A, for the 6th case from the left, the diagnosis is GBM, but the grade is III. In general, it seems "GBM" is equivalent to grade “IV” histology; how is the GBM designation made for this tumor that is grade III?

It probably cannot be determined whether the reduction in exclusivity of EGFR and NF1 changes in samples taken from recurrent tumors indicate co-occurring changes within individual cells, or a greater mixing of cells of exclusive subclones on a geographic basis.

In figure 5B (right), the TCGA class is indicated as light blue for most samples, but there is no light blue in the key. What should these be?

Please expand on the last sentence of the results section with an explanation (what transcriptomic findings indicate cell size/homogeneity):

Together these results indicate that NEU tumors are populated by smaller more homogenous cells and GPM tumors are made up of larger more heterogeneous cells which is consistent with the cell type signatures we identified on transcriptomic analysis.

It would be helpful for the authors to put their molecular evolution results in the context of additional studies previously published:

Wang et al., Cancer Cell. 2017 Jul 10;32(1):42-56.e6

Acta Neuropathol. 2018; 135(5): 649–670. Barthel, Wesseling, Verhaak

Korber et al., Cancer Cell Volume 35, Issue 4, 15 April 2019

(Verhaak) Kim et al., Genome Res. 2015 Mar; 25(3): 316–327

(Verhaak, GLASS) Varn et al. Nature. 2019 Dec; 576(7785): 112–120

Ann Oncol. Spiteri et al...Sottoriva, 2019 Mar; 30(3): 456–463.

Neuro Oncol. Abou-El-Ardat et al. (Barbara Klink) 2017 Apr; 19(4): 546–557.

Minor:

line 150: typo "mitochondrial" is misspelled

Figure 2: typo: C and D descriptions are switched relative to figure

Reviewer #2 (Remarks to the Author): expertise in evolution modelling and mixed effects models

In "Integrated molecular and multiparametric MRI mapping of high-grade glioma identifies regional biologic signatures", Hu et al. examine high grade glioma (HGG), conducting a series of analyses on conventional & advanced MRI technologies, spatially matched multi-region whole exome sequencing (WES), and the relationship between the two. After a well written review of the different MRI technologies (much appreciated by this MRI non-expert), the following analyses were conducted:

1. Comparison of WES and MRI feature intra-tumoral heterogeneity in IDH wild-type (IDHwt) compared to IDH mutant (IDHmut).

2. Comparison WES and MRI feature intra-tumoral heterogeneity and phylogenies in the contrast enhancing (CE), non-enhancing (NE), and combined regions in IDHwt samples.

3. Examination of the alteration profiles of EGFR and NF1 in IDHwt samples, with an extended analysis of a tumor (P065) that showed the unusual pattern of having heterogeneous patterns of mutual exclusivity of EGFR & NF1 mutations across sampled regions.

4. Examination of spatial and molecular heterogeneity of EGFR in a single treatment naïve tumor (P129).

5. Construction and analysis of a mixed effects model (MEM) to determine how driver mutations manifest as alterations in MRI features.

6. Using 34 IDHwt tumors to construct of an evolutionary model (i.e. phylogenetic tree) that recapitulates the order of mutations, followed by a deeper exploration of the NE regions.

7. Using molecular data to cluster of tumors into groups using a variety of approaches (most variable genes, TCGA classification, pathway based classification), and then determining if those groups vary across NE and CE regions

8. Examination of the relationship between genetic distance and physical distance in CE vs NE, and among the different molecular subtypes (i.e NEU, PPR, GPM, MTC).

9. Examination of the effect of pathway enrichment on spatially matched MRI features/metrics.

10. A through comparison of NE vs CE using DSC-MRI, grouped by molecular subtype (e.g. GPM and NEU)

While the results of the individual analyses are interesting, and the approach of combining multi-region WES with MRI appears to be novel, it's not entirely clear what the key take away is. The individual

results are never quite tied together into a cohesive “story”, other than NE are CE are different, and HGG exhibits molecular ITH (consistent with citations 42-45) which can manifest itself on MRI. Instead, it seems the goal is to conduct a suite of descriptive analyses that combine MRI image features/regions and spatially matched WES, but without a “bigger picture” to keep the analyses focused. The paper could likely be strengthened and more readable if the authors would provide hypotheses to help explain their findings. For example, there are several evolutionary analyses, often comparing CE to NE. However, there aren’t many attempts to explain what evolutionary processes (selection, competition, invasion, drift, etc…) are behind the observations. For example, could the authors speculate on what evolutionary processes are responsible for the observed increased burden of private mutations in NE compared to CE? Or, what can we learn from the four evolutionary models of HGG (Figure 6) other than there is more than one mutational route to HGG? Similarly, can the authors offer any hypotheses about why genetic distance and Euclidean distance are not significantly correlated in NE, when one might expect that they would be (as observed in CE)? Or, why does having extra copies of EGFR have a strong effect on T2W signals, i.e. what associated cellular/microenvironmental changes are occurring and how/why do they show up as different T2W signals? There are several such tantalizing findings, but they are not clearly brought together. If the authors could develop some hypotheses, they could then be used to help explain why NE and CE are different, not just that they are.

In addition to the above, it appears that some of the statements being made are not clearly supported:

• “These findings indicate that the high degree of regional heterogeneity of driver mutations in IDH-mutant glioma is especially pronounced in the NE.” (line 208). This statement follows a discussion of 3 IDH-mut tumors with EGFR alterations, but that is only 3 out of 11 (27.3%), so is it too much to say that heterogeneity in diver mutations is “especially pronounced”?

• “IDHwt tumors harbored a significantly higher proportion of private alterations in the NE (66.7%) compared to CE (49.3%)” (line 225), but there is not a test of significance.

• “IDH-mutant tumors demonstrated an increased burden of private mutations in the NE (41.43%) compared to the CE (13.14%) (Figure 2A)” (line 197). Again, no statistical comparisons were performed, so it isn’t clear how meaningful this difference is.

• “only paired CE samples maintained statistical significance upon stratification (Figure S5C)” (line 382). In this case the p-value is found in the supplemental figure, but it should also be in the main text.

• The section “Spatial and molecular heterogeneity of EGFR in GBM” (line 259) describes the molecular heterogeneity (e.g. EGFR variants), but there doesn’t appear to be quantification of spatial heterogeneity except for a reference to the example image. This could be quantified, maybe as the “patchiness” of EGFR mutations. However, quantification then invites statistical comparisons, but this analysis was only performed on 1 sample (P129). Would it be possible to conduct this analysis on more samples, or is P129 the only treatment naïve tumor in the cohort?

There are also a few suggestions. First, the integration of MRI and spatially matched multi-region WES is a highlight of this work. However, the process of “spatially matching” the two datasets isn’t clearly

described in the main text. It appears the "spatial matching" is referring to the fact that the position of the stereotactic biopsies was recorded via screen capture, and so the authors could use those biopsy locations to look at corresponding location in the in the MRI. The term "spatially matched" doesn't immediately imply that this is how the multiple samples and MRI were co-registered, and the process isn't described until the "Experimental Design and Methods/Multiregional glioma sample cohort" subsection (occurring after the results and discussion). As the integration of spatially matched MRI and multi-region WES is central to this work, perhaps it would be worth briefly describing this process before getting into the results, perhaps with a figure (maybe similar to the 3D images in Figure 5A).

This is a minor point, but the authors repeatedly describe the construction of phylogenetic trees from multi-region sampling (a common practice) as creating/inferring a "spatial evolutionary model". "Spatial evolutionary model" would seem to indicate that the authors used spatial measures to help construct the tree (e.g. distance between sampling points), or even simulated the spatial evolution of the tumor, but neither appear to be the case, as the authors used REVOLVER to construct the trees. Even the abstract for REVOLVER states "Multi-region sequencing allows inference of some temporal orderings of genomic changes within a tumour", but says nothing about space. "Spinning" phylogenetic trees as something else/more is a bit off-putting, as if the authors are attempting to make the work seem more novel than it is, i.e. trying to make it appear to be a spatial analysis/model (e.g. cellular neighborhood analysis, agent based model, etc…). The work is already good, and the trees informative, so I would suggest removing "spatial" from these descriptions, and simply refer to them as phylogenetic trees or evolutionary models.

In addition to the above, there are a few additional comments regarding specific lines and figures:

• Figure 1B: "number of samples per patient" doesn't have a color bar, presumably because the discrete colormap being used has too many colors. Maybe a continuous linear colormap, like viridis (or similar), would be better, as it would make it easier to determine if the number of samples is high, low, or somewhere in between.

• "We observed regional heterogeneity in genes associated with poor clinical prognosis in IDH-mutant glioma" (line 200). Can the heterogeneity be quantified (maybe Shannon or Simpson's Index?) and compared to wild-type IDH glioma samples?

• "predicted across 34 IDH wild-type informative patients" (line 324). What makes a patient "informative"?

• The legend for Figure S5C says each color is a different contrast region, but there is no color legend in the plot. From the main text it appears that CE is red. Relatedly, the p-value is in the figure, but not the main text. Please add a legend to the figure and the p-value in the main text.

• "we selected multiregional pairs including one CE and one NE samples" (line 391). How was the pair picked? Why only 1 pair per sample? Could the authors compare all CE and NE pairs from the tumor, just as they had done in the previous analyses. Do the results hold up?

• In the section “Samples classified as NEU exhibit the highest burden of private mutations in the NE region” it might be worth mentioning that the genetic distance is 1 – Jaccard index on the genetic alteration patterns. There’s quite a bit of evolutionary analyses here, so any (tumor) evolutionary biologists might be interested to know without having to search for it in the methods section.

• Instead of stating that they “applied a recently reported computational method using transfer machine learning to identify repeated molecular alteration trajectories” (line 317), they could explicitly state that they used REVOLVER for this analysis, for the same reasons as described in the point above.

Reviewer #3 (Remarks to the Author): expertise in glioma imaging

In this study the authors have taken multiple image-guided biopsies and performed multi-omic analysis of these samples as well as correlating this biological data with multi-parametric imaging. The authorship is extensive, but this reflects the multiple elements of this paper – the authorship includes several leading authorities in their field.

The authors sample both enhancing and non-enhancing tumour. The focus on non-enhancing tumour is important. As you would usually expect the contrast enhancing component to be removed the key aspect of this study is the understanding of the ‘residual tumour’ left in the non-enhancing tumour regions. In addition, they study both IDH-wild type and IDH-mutant tumour and develop a genotype for poor prognosis IDH-mutant tumours.

Methodologically it involves a good tumour sample size with a number of biopsies per patient. The number of samples in the non-enhancing tumour is less than samples from the enhancing tumour but still must be the largest study of its type. The image-guided biopsy is a standard method and would provide accurate correlations. The only minor point worth clarifying is if they recruited cystic tumours. These can lead to marked brain shifts that can make analysis difficult.

Overall, this is an important study. It covers a lot of different areas, and this does make it difficult to read and follow. I wonder if the use of more sub-sections would improve clarity. I do have specific issues:

1. The abstract reads of a study that is looking only at non-enhancing tumour, and that the main thrust of the study is looking at correlations with imaging. It implies that there are 313 multi-regional biopsies from the non-enhancing region (this is incorrect, there are 111 biopsies from this region). This isn’t what the paper contains. The paper covers both contrast enhancing and non-enhancing tumour regions, and the focus of the results is the multi-omics data from these regions. I understand the word limit issues, but the abstract should reflect the main study.

2. The issue of studying non-enhancing/invasive tumour is that this region is likely to include both tumour cells and normal cells. There is no description on how they can differentiate these cell types (this is actually very difficult). You could argue that this may reflect the mosaicism that is found. Even

histological analysis showing this is gross tumour or invasive tumour might be useful. There is a need to say how they dealt with this 'dilutional' effect of mixed tumour and normal cells.

3. The methods describe analysis of DTI data. There is mention of mean diffusivity results in the conclusion, but I can't find any analysis of the DTI data (mean diffusivity or fractional anisotropy) anywhere in the results or in the supplementary results. Is this reported? If not, does it need to be there?

Reviewer #4 (Remarks to the Author): expertise in glioma multi-omics and evolution

This is a very important study that will represent a great resource for the community. It is relevant both for the multi-omic characterization of a large cohort of HGG in multiple regions as well as for the imaging features/metadata. I have some minor comments and some concerns I'd like to see addressed:

- Line 190: the sentence that IDH-mutation confers good prognosis is unfortunate. As these authors know, it simply identifies a different type of glioma. The prognostic comparator is "not having a glioma at all". It is an important point, because too often in the field IDH is thought as a "good" event, which is of course incorrect. Please rephrase.

- Lines 264 and 333: EGFR should be in italics

- Line 300: I think the authors meant "genotypes" rather than "phenotypes".

- The cohort would appear too small to construct 4 groups of molecular evolution of gliomas as done in Fig6. I would suggest that the authors focus the figure on describing the NE regions genetics (the strength of their study) and diminish the emphasis on the 4 molecular groups of IDH wildtype, which would require a larger cohort for validation.

- It is unclear to this reviewer how one can train an imaging model on a set of molecular features in a given dataset, identify imaging-molecular correlates and then claim specific associations without having an independent/external dataset for validation, on which the model was not trained. I also have concerns regarding identifying molecular states of cancer cells based on imaging: the imaging may well capture a specific tissue feature (e.g. hypoxia or normal brain parenchyma), the cancer cell states may be associated with such features, but that does not mean that imaging may capture cell states. The authors may want to be more cautious in their assumptions/statements.

NCOMMS-23-06819-T

We thank the reviewers for their careful review of this study. Our detailed responses to the reviewer's comments are listed below (**blue text: Note – text lines refer to cleaned version of the manuscript**):

Reviewer 1

A few questions arise:

1. Was the designation "high-grade" determined by imaging (contrast enhancement), or based on histologic verification? What were the criteria for calling a glioma high-grade?

   High-grade designation was based on histology. High grade gliomas are classified as any grade 3 or grade 4 gliomas by pathologic diagnosis, which we have made clearer in the methods section (lines 577-581). In figure 2 (IDH mutant cohort), the grade 2 tumor (G33) has been moved to Supplementary Figure 1.

2. In figure 2, only four of the eleven tumors demonstrate both CE and NE samples. Does this have an impact on the interpretation of the comparison of molecular findings in CE and NE areas? Can findings in entirely NE tumors be extrapolated to NE areas of tumors with CE? Do the tumors without sampling of CE have unsampled areas of CE? Could tumors that are entirely non-CE have different properties (more heterogeneity) than those that have CE? In other words, are these different kinds of tumors, rather than different kinds of areas in a single type of tumor?

   We have revised Figure 2 to include only grade 3 and 4 IDH-mutant tumors that have both CE and NE samples available. We believe the strength of Figure 2 is the description of the intra- and inter-tumor heterogeneity of alterations in IDH-mutant tumors. Reviewer 1 raises a great question; we agree that there are limitations on the interpretation of the biology of the NE and CE in the IDH mutant cohort shown in Figure 2. It is known that IDH mutant tumors accumulate contrast enhancement as they become more aggressive (Jonsson *et al.* Clin Cancer Res. 2019; PMID 31263031), but it is not clear which parts of the NE could become more aggressive / invasive as the tumor progresses. Thus, we profiled all NE samples regardless of CE co-occurring in the same tumor, considering NE in the IDH-mutant tumors as potential invasive populations. We have moved tumors without both CE and NE samples from Figure 2 to supplemental figure 2 to improve the focus and readability of the results text.

3. For recurrent tumors, for example in figures 2 and 3, is the CE due to inherent properties of the tumor or due to treatment/prior surgery, or both? The interpretation suggests that CE is strictly an indication of tumor properties, and molecular features seem to be interpreted in this light. Please clarify.

The authors acknowledge the possibility that contrast enhancement in recurrent tumors may be a result of post-treatment changes. Samples collected for this study were all reviewed for presence of tumor by a neuro-pathologist (K.D., J.E.) prior to submission for sample processing (lines 577-580). We only examined imaging data from biopsy-proven sites of tumor.

4. The NE oligodendroglioma in figure 2, second from right, appears to be graded "IV." Oligodendrogliomas are graded 2 or 3; please clarify the meaning of a grade IV oligodendroglioma, particularly in the absence of CE.

   This tumor is a grade 2, we have amended the figure accordingly with this tumor moved to the Supplementary Figure 2.

5. Could there be shift or other reasons for imperfect localization occurring that prevents perfect correlation of the biopsy based on the pre-op MRI?

   We recognize the potential for brain shifts to occur during open craniotomy, which could contribute to misregistration errors in MRI-guided neuronavigation and biopsy sample localization. To offset these potential misregistration differences, the neurosurgeons at BNI and Mayo Clinic leverage their clinical experience by employing techniques such as small craniotomy sizes to minimize brain shift and visual validation of intracranial neuroanatomic landmarks to confirm stereotactic image localization. During image analysis and MRI feature extraction, we took further measures to minimize any potential effects of brain shift and misregistration. Specifically, we designed the region of interest (ROI) sizes (~ 0.28cc) to be larger than the typical volumes of biopsy samples (~0.125 cc; see Experimenal Designs and Methods, line 594). This size difference allows the ROI size to accommodate for potential shifts of the biopsy sample within the ROI, which minimizes the effects of potential misregistration on subsequent spatial correlative analyses.

6. As the authors describe, CE is believed to result from breakdown of the blood-brain-barrier. For cases where the grade is II (no histologic evidence of BBB breakdown) but the imaging is CE, there is a lack of correlation of histology and radiology.

   There is one tumor with NE and CE samples (P158, IDH mutant) which was originally labeled as grade 2-3. This tumor has been appropriately re-labeled to be a grade 3.

   Similarly, many cases in the IDH-wt cohort show grade IV histology in NE areas. That leads to the question of with how much confidence claims can be about the "periphery" of the tumors.

Regardless of histologic features within individual biopsies, tumors were labeled according to their clinical diagnosis, *i.e.* all research samples from a grade 4 tumor were considered grade 4 histology. We acknowledge that there can be regions of NE and CE within the bulk of the tumor. All our samples designated NE and CE were reviewed by a board-certified neuro-radiologist to determine that the NE samples were indeed localized outside of the enhancing components of the tumor.

7. In Figure 4A, for the 6th case from the left, the diagnosis is GBM, but the grade is III. In general, it seems "GBM" is equivalent to grade “IV” histology; how is the GBM designation made for this tumor that is grade III?

   We thank the reviewer for catching this error. This tumor is IDH wild-type, thus we have re-labeled this tumor as a grade 4 in Figure 4A.

8. It probably cannot be determined whether the reduction in exclusivity of EGFR and NF1 changes in samples taken from recurrent tumors indicate co-occurring changes within individual cells, or a greater mixing of cells of exclusive subclones on a geographic basis.

   The authors are in complete agreement with this point and will clarify the interpretation of Figure 4 in both results and discussion. We have communicated that we cannot make the conclusion that *EGFR* and *NF1* alterations co-occur in the same cell (line 521-523). We aim to further clarify this point with single cell and spatial transcriptomic studies in future cohorts.

9. In figure 5B (right), the TCGA class is indicated as light blue for most samples, but there is no light blue in the key. What should these be?

   This error has been corrected and the light blue bar is now labeled as TCGA Classical.

10. Please expand on the last sentence of the results section with an explanation (what transcriptomic findings indicate cell size/homogeneity): Together these results indicate that NEU tumors are populated by smaller more homogenous cells and GPM tumors are made up of larger more heterogeneous cells which is consistent with the cell type signatures we identified on transcriptomic analysis.

    We have clarified within the results text and added a supplementary figure 18 to the transcriptomic analysis that supports this explanation (lines 497-501).

11. It would be helpful for the authors to put their molecular evolution results in the context of additional studies previously published:

We thank the reviewer for bringing these important studies to our attention. We have included the following citations if they were missing previously, and expanded upon our discussion of the citations below that were already incorporated in our results or discussion.

Emphasize these citations where they are relevant in the results (ie the labels, inclusion in analysis)

- Wang et al., Cancer Cell. 2017 Jul 10;32(1):42-56.e6 [line 517; reference 65]

o Acta Neuropathol. 2018; 135(5): 649–670. Barthel, Wesseling, Verhaak [line 513; reference 63]

o Korber et al., Cancer Cell Volume 35, Issue 4, 15 April 2019 [line 232, reference 31]

o (Verhaak) Kim et al., Genome Res. 2015 Mar; 25(3): 316–327 [line 382, reference 45], also GLASS here, [reference 6]

o Varn et al. Nature. 2019 Dec; 576(7785): 112–120 [line 558, reference 69]

o Ann Oncol. Spiteri et al...Sottoriva, 2019 Mar; 30(3): 456–463. [line 513, reference 62]

o Neuro Oncol. Abou-El-Ardat et al. (Barbara Klink) 2017 Apr; 19(4): 546–557. [line 134, reference 7]

12. Minor: line 150: typo "mitochondrial" is misspelled. This has been corrected.
13. Figure 2: typo: C and D descriptions are switched relative to figure. This has been corrected.

<u>Reviewer 2</u>

14. While the results of the individual analyses are interesting, and the approach of combining multi-region WES with MRI appears to be novel, it's not entirely clear what the key take away is. The individual results are never quite tied together into a cohesive "story", other than NE are CE are different, and HGG exhibits molecular ITH (consistent with citations 42- 45) which can manifest itself on MRI. Instead, it seems the goal is to conduct a suite of descriptive analyses that combine MRI image features/regions and spatially matched WES, but without a "bigger picture" to keep the analyses focused.

    We thank the reviewer for this comment and have revised the manuscript to improve emphasis on main findings for a more cohesive story. The focus of this work is the comprehensive study of the invasive NE region, an enigmatic and poorly studied component of high-grade gliomas. We have revised the manuscript according to highlight cohesive story.

15. The paper could likely be strengthened and more readable if the authors would provide hypotheses to help explain their findings. For example, there are several evolutionary analyses, often comparing CE to NE. However, there aren't many attempts to explain what evolutionary processes (selection, competition, invasion, drift, etc...) are behind the observations. For example, could the authors speculate on what evolutionary processes are responsible for the observed increased burden of private mutations in NE compared to CE?

    We have revised the discussion to improve clarity on our hypotheses regarding our conclusions. Please see lines [538-545] in the Discussion.

    Or, what can we learn from the four evolutionary models of HGG (Figure 6) other than there is more than one mutational route to HGG?

    We have revised the description of the four evolutionary models and proposed the hypothesis of a dichotomous activation of PI3K and RTK/RAS pathways along two main alternative longitudinal branches of tumor evolution (Result Section, lines 344-352; Discussion Section, 523-526)

16. Similarly, can the authors offer any hypotheses about why genetic distance and Euclidean distance are not significantly correlated in NE, when one might expect that they would be (as observed in CE)?

    We have more clearly articulated our hypothesis regarding the lack of correlation in the NE at lines 538-545. In brief, the Euclidean distance as a straight-line distance measure is not as reflective of non-linear tumor cells growth paths. Because the bulk CE is an outward expansion driven by cell proliferation, it is likely better represented by linear distance than the NE. In the NE, tumors likely take a more tortuous path where Euclidean distance is an underestimate of the actual distance traveled.

17. Or, why does having extra copies of EGFR have a strong effect on T2W signals, i.e., what associated cellular/microenvironmental changes are occurring and how/why do they show up as different T2W signals?

    This result was found during our screening MEM model which incorporated many genetic factors. Unfortunately, due to sample size, MEM models with this many terms are hard to investigate for specific genotypic imaging signatures because the cohort is subdivided into so many buckets. On examination of EGFR alone or in pairwise combinations we did not see significance of T2W or rCBV again. We take this to mean that EGFR does have an effect, but the specifics of this effect are due to combinatorial genotypes that cross >2 genes or other unassayed covariates. We have elaborated on this further on lines 293-299.

18. There are several such tantalizing findings, but they are not clearly brought together. If the authors could develop some hypotheses, they could then be used to help explain why NE and CE are different, not just that they are.

    We have expanded our discussion to include reasons for the differences between NE vs CE and have added relevant references as pointed out by Reviewer 1, point 11.

19. In addition to the above, it appears that some of the statements being made are not clearly supported:
    "These findings indicate that the high degree of regional heterogeneity of driver mutations in IDH-mutant glioma is especially pronounced in the NE." (line 208). This statement follows a discussion of 3 IDH-mut tumors with EGFR alterations, but that is only 3 out of 11 (27.3%), so is it too much to say that heterogeneity in diver mutations is "especially pronounced"?

    We agree with the reviewer on this language, we have deleted this sentence.

    "IDHwt tumors harbored a significantly higher proportion of private alterations in the NE (66.7%) compared to CE (49.3%)" (line 225), but there is not a test of significance.

    The proportion of private alterations in IDHwt glioma is significantly different between CE and NE (two-sided Fisher's exact test p = 4.85e-43) as now indicated in the figure legend.

    "IDH-mutant tumors demonstrated an increased burden of private mutations in the NE (41.43%) compared to the CE (13.14%) (Figure 2A)" (line 197). Again, no statistical comparisons were performed, so it isn't clear how meaningful this difference is

    The proportion of private mutations in IDH-mutant glioma is significantly different between CE and NE (two-sided Fisher's exact test p = 3.43e-67), as now indicated in the figure legend.

20. The section "Spatial and molecular heterogeneity of EGFR in GBM" (line 259) describes the molecular heterogeneity (e.g. EGFR variants), but there doesn't appear to be quantification of spatial heterogeneity except for a reference to the example image. This could be quantified, maybe as the "patchiness" of EGFR mutations. However, quantification then invites statistical comparisons, but this analysis was only performed on 1 sample (P129). Would it be possible to conduct this analysis on more samples, or is P129 the only treatment naïve tumor in the cohort?

The purpose of Figure 5 is to highlight the intratumor heterogeneity in EGFR in one patient, P129. We chose P129 due to the abundance of biopsies; other tumors did not have as many samples available. In our cohort, EGFR harbored somatic genetic alterations in 26 out of 50 multiregional IDH wt cases, with 92% of mutant cases (23 out of 26) showing molecular intratumor heterogeneity in the EGFR locus across the multiregional samples (two-sided Fisher's exact test p = 2.43e-11). See line 270.

There are also a few suggestions.

21. First, the integration of MRI and spatially matched multi- region WES is a highlight of this work. However, the process of "spatially matching" the two datasets isn't clearly described in the main text. It appears the "spatial matching" is referring to the fact that the position of the stereotactic biopsies was recorded via screen capture, and so the authors could use those biopsy locations to look at corresponding location in the in the MRI. The term "spatially matched" doesn't immediately imply that this is how the multiple samples and MRI were co-registered, and the process isn't described until the "Experimental Design and Methods/Multiregional glioma sample cohort" subsection (occurring after the results and discussion). As the integration of spatially matched MRI and multi-region WES is central to this work, perhaps it would be worth briefly describing this process before getting into the results, perhaps with a figure (maybe similar to the 3D images in Figure 5A).

    We have provided a new supplementary figure 2 describing the process of spatially matching the localized MRI feature extraction with the stereotactic biopsy locations.

22. This is a minor point, but the authors repeatedly describe the construction of phylogenetic trees from multi-region sampling (a common practice) as creating/inferring a "spatial evolutionary model". "Spatial evolutionary model" would seem to indicate that the authors used spatial measures to help construct the tree (e.g. distance between sampling points), or even simulated the spatial evolution of the tumor, but neither appear to be the case, as the authors used REVOLVER to construct the trees. Even the abstract for REVOLVER states "Multi-region sequencing allows inference of some temporal orderings of genomic changes within a tumour", but says nothing about space. "Spinning" phylogenetic trees as something else/more is a bit off-putting, as if the authors are attempting to make the work seem more novel than it is, i.e. trying to make it appear to be a spatial analysis/model(e.g. cellular neighborhood analysis, agent based model, etc...). The work is already good, and the trees informative, so I would suggest removing "spatial" from these descriptions, and simply refer to them as phylogenetic trees or evolutionary models.

We thank the reviewer for this comment, and we have revised the manuscript and figure legends, removing the word "spatial" from any reference to spatial evolution from the description of the evolutionary model analysis.

In addition to the above, there are a few additional comments regarding specific lines and figures:

23. “Figure 1B: “number of samples per patient” doesn’t have a color bar, presumably because the discrete colormap being used has too many colors. Maybe a continuous linear colormap, like viridis (or similar), would be better, as it would make it easier to determine if the number of samples is high, low, or somewhere in between.

    We have modified the figure accordingly.

24. “We observed regional heterogeneity in genes associated with poor clinical prognosis in IDH-mutant glioma” (line 200). Can the heterogeneity be quantified (maybe Shannon or Simpson’s Index?) and compared to wild-type IDH glioma samples?

    Due to the limited number of IDH mutant samples, we have emphasized that the heterogeneity within these samples is descriptive only.

25. “predicted across 34 IDH wild-type informative patients” (line 324). What makes a patient “informative”?

    We have removed the word “informative” and clarified more specifically that the patients used in this analysis are those with multiregional samples available and with more than one truncal driver event identified.

26. The legend for Figure S5C says each color is a different contrast region, but there is no color legend in the plot. From the main text it appears that CE is red. Relatedly, the p- value is in the figure, but not the main text. Please add a legend to the figure and the p- value in the main text.

    These changes have been implemented.

27. “we selected multiregional pairs including one CE and one NE samples” (line 391). How was the pair picked? Why only 1 pair per sample? Could the authors compare all CE and NE pairs from the tumor, just as they had done in the previous analyses. Do the results hold up?

We apologize for the confusion. All CE and NE pairs were used for this analysis. We have clarified this in the text (line 398):
*"we selected **all** multiregional pairs **that included at least** one CE and one NE sample."*.

28. In the section "Samples classified as NEU exhibit the highest burden of private mutations in the NE region" it might be worth mentioning that the genetic distance is 1 – Jaccard index on the genetic alteration patterns. There's quite a bit of evolutionary analyses here, so any (tumor) evolutionary biologists might be interested to know without having to search for it in the methods section.

    We thank the reviewer for this comment, and we have added the genetic distance description in the manuscript. See line 385.

29. Instead of stating that they "applied a recently reported computational method using transfer machine learning to identify repeated molecular alteration trajectories" (line 317), they could explicitly state that they used REVOLVER for this analysis, for the same reasons as described in the point above.

    We thank the reviewer for this suggestion, and we have explicitly stated in the revised version of the manuscript that REVOLVER has been used for our analysis (line 335).

<u>Reviewer 3</u>

Overall, this is an important study. It covers a lot of different areas, and this does make it difficult to read and follow. I wonder if the use of more sub-sections would improve clarity. I do have specific issues:

30. The abstract reads of a study that is looking only at non-enhancing tumour, and that the main thrust of the study is looking at correlations with imaging. It implies that there are 313 multi-regional biopsies from the non-enhancing region (this is incorrect, there are 111 biopsies from this region). This isn't what the paper contains. The paper covers both contrast enhancing and non-enhancing tumour regions, and the focus of the results is the multi-omics data from these regions. I understand the word limit issues, but the abstract should reflect the main study.

    We have modified the abstract to reflect that we profile 111 biopsies from the NE region.

31. The issue of studying non-enhancing/invasive tumour is that this region is likely to include both tumour cells and normal cells. There is no description on how they can differentiate these cell types (this is actually very difficult). You could argue that this may reflect the

mosaicism that is found. Even histological analysis showing this is gross tumour or invasive tumour might be useful. There is a need to say how they dealt with this 'dilutional' effect of mixed tumour and normal cells.

In agreement with the reviewer's comment, we carefully considered the aspect of tumor and non-tumor composition of the bulk samples we analyzed. We estimated the tumor purity of each sample by the analysis of WES copy number (as stated in the method section) and reported it as a track in the molecular profiling figures. Moreover, we generated the digital cytometry profiles by performing the gene expression deconvolution analysis of tumor and non-tumor cell types using CybersortX (Figure S18).

32. The methods describe analysis of DTI data. There is mention of mean diffusivity results in the conclusion, but I can't find any analysis of the DTI data (mean diffusivity or fractional anisotropy) anywhere in the results or in the supplementary results. Is this reported? If not, does it need to be there?

    Please refer to the subsection entitled "EGFR and CDKN2A alterations drive variance in advanced imaging features in the NE region". In that subsection, we describe how the NE tumor populations predicted to display increased cellular proliferation by mean diffusivity (MD) MRI metrics were uniquely associated with EGFR amplification and CDKN2A homozygous deletion. FA did not show a significant association with our genotypes or pathway-based classifications (See Figure S3, S5, S10, S11).

Reviewer 4

This is a very important study that will represent a great resource for the community. It is relevant both for the multi-omic characterization of a large cohort of HGG in multiple regions as well as for the imaging features/metadata. I have some minor comments and some concerns I'd like to see addressed:

33. Line 190: the sentence that IDH-mutation confers good prognosis is unfortunate. As these authors know, it simply identifies a different type of glioma. The prognostic comparator is "not having a glioma at all". It is an important point, because too often in the field IDH is thought as a "good" event, which is of course incorrect. Please rephrase.

    We have rephrased to indicate that we are referring to the comparison of prognoses of two gliomas: IDH wild type and IDH mutant.

34. Lines 264 and 333: EGFR should be in italics

    We have made this correction.

35. Line 300: I think the authors meant “genotypes” rather than “phenotypes”.

We have made the suggested changes.

36. The cohort would appear too small to construct 4 groups of molecular evolution of gliomas as done in Fig6. I would suggest that the authors focus the figure on describing the NE regions genetics (the strength of their study) and diminish the emphasis on the 4 molecular groups of IDH wildtype, which would require a larger cohort for validation.

In agreement with the reviewer’s suggestion, we modified the figure 6 to emphasize the functional consequences of NE specific genetic alterations (panel A), and we downplayed the results of evolutionary models (with the supervised clustering moved to supplementary figure S6).

37. It is unclear to this reviewer how one can train an imaging model on a set of molecular features in a given dataset, identify imaging-molecular correlates and then claim specific associations without having an independent/external dataset for validation, on which the model was not trained.

We appreciate the reviewer’s attention to our methods and the important issue of reproducibility. We would like to clarify that our analysis does not “train” as mixed-effect modeling is a type of regression analysis which can be used in machine learning, but our use of it is purely mathematical. As clarification, this cohort is not optimized for training predictive models and is only to quantify associations. In this way, our analysis is more similar to describing -omic features with statistical measures or quantifying relationships with microscopy than it is to machine learning where the need for a “test” set is crucial because the algorithm will be deployed for predictive purposes and directly affect decision making. Unfortunately, this dataset is one of a kind in the types of MRI features measured, the regions sampled, and the -omic data collected, so we are unable to externally validate. We are continuing to collect samples and look forward to use of this cohort as a validation set for future studies.

38. I also have concerns regarding identifying molecular states of cancer cells based on imaging: the imaging may well capture a specific tissue feature (e.g. hypoxia or normal brain parenchyma), the cancer cell states may be associated with such features, but that does not mean that imaging may capture cell states. The authors may want to be more cautious in their assumptions/statements.

We agree with this comment and have addressed this shortcoming of interpretation of imaging associations in the discussion. Of the associations identified in this study, we are

not able to establish a causal relationship, but we do pose plausible hypotheses informed by existing literature (lines 558-561).

REVIEWERS' COMMENTS

Reviewer #1 (Remarks to the Author):

Thank you for addressing my comments.

Reviewer #2 (Remarks to the Author):

I would like to thank the authors for all the work they've put into addressing my comments. While the paper remains quite dense and highly descriptive, this revision feels more cohesive, due to the expanded discussion and extra comments throughout. Combined, these revisions help tie together the many interesting finds into a comprehensive story. I do have a few remaining minor comments/suggestions:

• The authors mention there are "median = 4 samples per tumor, range = 1-13 samples per tumor" (line 183), but could they provide such a description per region, i.e. median CE samples per tumor and median NE samples per tumor?

• I'd suggest the text describing the detailed analysis of patient P129 belongs in its own paragraph (starting at the end of line 270), possibly with a statement about why it was singled out for a more detailed analysis.

• The authors state: "Due to sample size limitations MEM models with over two genes could not be specifically interrogated for effect directionality and gene-gene interactions" (line 297), but then go on to say "mixed effect models were generated examining EGFR copy number in combination with NF1, TP53, and CDKN2A copy number" (line 305). Could the authors please clarify how they overcame the sample size limitations in the second analysis?

• I appreciate the authors sharing the code and data. However, I noticed that an author field is missing in the scripts and README files. I might suggest adding such a field to the scripts and/or README files to more specifically credit the individuals who wrote the code (in addition to the general attributions in the "Software" part of the Contributions section).

Reviewer #3 (Remarks to the Author):

The authors have incorporated all the comments I had made to their first draft.

This is still a challenging paper to read but it now reads better. The authors have done a better job incorporating the imaging into the article rather than as a 'after thought' as it was. DTI is not used as much as it should be, but does now (barely) feature.

Reviewer #4 (Remarks to the Author):

The authors have addressed all my concerns.

NCOMMS-23-06819-T

We thank Reviewer 2 for the careful review of this study. Our detailed responses to the reviewer's comments are listed below:

Reviewer 2

1. The authors mention there are "median = 4 samples per tumor, range = 1-13 samples per tumor" (line 183), but could they provide such a description per region, i.e. median CE samples per tumor and median NE samples per tumor?

We have added the updated information (lines 181-184).

2. I'd suggest the text describing the detailed analysis of patient P129 belongs in its own paragraph (starting at the end of line 270), possibly with a statement about why it was singled out for a more detailed analysis.

We have updated the text to reflect the reviewer's suggestion (lines 272-274)

3. The authors state: "Due to sample size limitations MEM models with over two genes could not be specifically interrogated for effect directionality and gene-gene interactions" (line 297), but then go on to say "mixed effect models were generated examining EGFR copy number in combination with NF1, TP53, and CDKN2A copy number" (line 305). Could the authors please clarify how they overcame the sample size limitations in the second analysis?

We have clarified this accordingly (lines 305-309).

4. I appreciate the authors sharing the code and data. However, I noticed that an author field is missing in the scripts and README files. I might suggest adding such a field to the scripts and/or README files to more specifically credit the individuals who wrote the code (in addition to the general attributions in the "Software" part of the Contributions section).

We have credited the individuals for the code scripts and README files.
